# Supplementary material for: Unleashing phosphorus mononitride
Source: Nat Commun. 2025 Jul 1;16:5596. doi: 10.1038/s41467-025-60669-6 (PMC12215955; doi:10.1038/s41467-025-60669-6)
Supplement: Supplementary file 1 — Supplementary Information [file 41467_2025_60669_MOESM1_ESM.pdf]

Supplementary Information

For

Unleashing Phosphorus Mononitride

Simon Edin,[1] Christian Sandoval-Pauker,[2] Nathan J. Yutronkie,[3] Zoltan Takacs,[1] Fabrice Wilhelm,[3] Andrei Rogalev,[3] Balazs Pinter,[2,4] Kasper S. Pedersen,[5,*] Anders Reinholdt[1,*]

1 Centre for Analysis and Synthesis
Department of Chemistry
Lund University
Naturvetarvägen 22, 22100 Lund, Sweden
anders.reinholdt@chem.lu.se

2 Department of Chemistry and Biochemistry
University of Texas at El Paso
El Paso, TX 79968, USA

3 ESRF- The European Synchrotron Radiation Facility
CS 40220, 38043 Grenoble Cedex 9, France

4 Current affiliation: European Research Council Executive Agency[#]

5 Department of Chemistry
Technical University of Denmark
Kemitorvet 207, DK-2800 Kgs. Lyngby, Denmark
kastp@kemi.dtu.dk

\# Disclaimer: The views expressed are purely those of the authors and may not in any circumstances be regarded as stating an official position of the ERCEA and the European Commission.

1 Contents

1 Contents **2**

2 Materials and Methods **4**

3 Syntheses **7**

3.1 Synthesis of [(salNdipp)$_2$(Cl)OsN] (**1**) 7

3.2 Synthesis of [(salNdipp)$_2$(OTf)OsN] (**2**) 8

3.3 Reaction of [(salNdipp)$_2$(OTf)OsN] (**2**) with Na(OCP) 8

3.4 Synthesis of [(salNdipp)$_2$(DMAP)Os(NP)] (**3**) 9

Method A (one pot from (Bu_4N)[Os(N)Cl_4]. 9

Method B (from (Bu_4N)[Os(^{15}N)Cl_4]. 10

3.5 Synthesis of [(salNdipp)$_2$(DMAP)Os(NPS_2)] (**4**) 12

3.6 Synthesis of [(salNdipp)$_2$(DMAP)Os(NPCl)] (**5**) 13

3.7 Synthesis of [(salNdipp)$_2$(DMAP)Os(N_4P)] (**6**) 14

3.8 Reduction of [(salNdipp)$_2$(DMAP)Os(N_4P)] (**6**) with KC_8 15

3.9 Thermal decomposition of [(salNdipp)$_2$(DMAP)Os(N_4P)] (**6**) to form [(salNdipp)$_2$(DMAP)Os(NP)] (**3**) and [(salNdipp)$_2$(DMAP)Os(N_2)] (**7**). 15

4 NMR Spectroscopy **16**

4.1 NMR Spectral Data for [(salNdipp)$_2$(Cl)OsN] (**1**) 16

4.2 NMR Spectral Data for [(salNdipp)$_2$(OTf)OsN] (**2**) 21

4.3 NMR Spectral Data for [(salNdipp)$_2$(DMAP)Os(NP)] (**3**) 27

4.4 NMR Spectral Data for [(salNdipp)$_2$(DMAP)Os(NPS_2)] (**4**) 35

4.5 NMR Spectral Data for [(salNdipp)$_2$(DMAP)Os(NPCl)] (**5**) 43

4.6 NMR Spectral Data for [(salNdipp)$_2$(DMAP)Os(N_4P)] (**6**) 44

5 IR Spectroscopy **45**

5.1 IR Spectral Data for [(salNdipp)$_2$(DMAP)Os(NP)] (**3**) 45

5.2 IR Spectrum of [(salNdipp)$_2$(DMAP)Os(N_4P)] (**6**) treated with KC_8 47

5.3 IR Spectrum of [(salNdipp)$_2$(OTf)Os(N)] (**2**) treated with Na(OCP). 48

5.4 IR Spectrum of insoluble product from thermal decomposition of [(salNdipp)$_2$(DMAP)Os(N_4P)] (**6**). 49

5.5 IR Spectrum of products from thermal decomposition of [(salNdipp)$_2$(DMAP)Os(N_4P)] (**6**).50

6 UV-vis Spectroscopy **51**

6.1 UV-vis Spectral Data for [(salNdipp)$_2$(OTf)Os(N)] (**2**) 51

6.2 UV-vis Spectral Data for [(salNdipp)$_2$(DMAP)Os(NP)] (**3**) 52

6.3 UV-vis Spectral Data for [(salNdipp)$_2$(DMAP)Os(NPS$_2$)] (**4**) 53

6.4 UV-vis Spectral Data for [(salNdipp)$_2$(DMAP)Os(NPCl)] (**5**) 54

6.5 UV-vis Spectral Data for [(salNdipp)$_2$(DMAP)Os(N$_4$P)] (**6**) 55

7 Reactivity Studies 56

7.1 Reaction between [(salNdipp)$_2$(OTf)Os(N)] (**2**) and Na(OCP). 56

7.2 Sulfur atom transfer between [(salNdipp)$_2$(DMAP)Os(^{15}NP)] (**3-^{15}N**) and [(salNdipp)$_2$(DMAP)Os(NPS$_2$)] (**4**). 57

7.3 Desulfurization of [(salNdipp)$_2$(DMAP)Os(NPS$_2$)] (**4**) using PPh$_3$ 59

7.4 Thermal decomposition of [(salNdipp)$_2$(DMAP)Os(N$_4$P)] (**6**) 61

8 Crystallographic Data 62

8.1 Crystallographic Tables 62

8.2 Thermal Ellipsoid Plot of [(salNdipp)$_2$(Cl)Os≡N] (**1**) 64

8.3 Thermal Ellipsoid Plot of [(salNdipp)$_2$(OTf)Os≡N] (**2**) 65

8.4 Thermal Ellipsoid Plot of [(salNdipp)$_2$(DMAP)Os(NP)] (**3**) 66

8.5 Thermal Ellipsoid Plot of [(salNdipp)$_2$(DMAP)Os(NPS$_2$)] (**4**) 67

8.6 Thermal Ellipsoid Plot of [(salNdipp)$_2$(DMAP)Os(NPCl)] (**5**) 68

8.7 Thermal Ellipsoid Plot of [(salNdipp)$_2$(DMAP)Os(η^1-N$_4$P)] (**6**) 69

8.8 Thermal Ellipsoid Plot of [(salNdipp)$_2$(DMAP)Os(N$_2$)] (**7**) 70

8.9 Notes on A- and B-Level Alerts in CheckCif Reports 71

9 Computational Studies 74

9.1. Computational methodology 74

9.2 Computational data 75

10 References 91

2 Materials and Methods

All synthetic operations were performed in Vigor glove boxes under a purified nitrogen atmosphere. Hexane (Fisher Scientific), and toluene (Fisher Scientific), were purified with a SPS system (MBraun SPS 800). Tetrahydrofuran (Sigma Aldrich), and diethyl ether (Sigma Aldrich) were stored over sodium with benzophenone as indicator, distilled by trap-to-trap transfer *in vacuo*, and degassed by freeze-pump-thaw cycles. The anhydrous solvents were stored over 4 Å molecular sieves (Fisher Scientific). Anhydrous 1,4-dioxane (99.8%, Thermo Scientific, Stab. 1-3 ppm BHT), was used as received. Benzene-d_6 and THF-d_8 (Sigma Aldrich) were stored over a potassium mirror overnight, sublimed/distilled by trap-to-trap transfer *in vacuo*, and degassed by freeze-pump-thaw cycles. Celite and 4 Å molecular sieves were activated *in vacuo* overnight at 175 °C. $(Bu_4N)[Os(N)Cl_4]$,[1] H(salNdipp),[2] Na(OCP) · 2.5 dioxane,[3] and KC_8,[4] were prepared according to published procedures. NaH (90%, Sigma Aldrich), AgOTf (99%, Sigma Aldrich), trityl chloride (97%, Sigma Aldrich), Me_3SiN_3, (95%, Sigma Aldrich) and $(Bu_4N)(N_3)$ (Sigma Aldrich) were purchased from commercial vendors and used as received. Sulfur (Fisher Scientific) was recrystallized from hot toluene and dried *in vacuo* at 50 °C for 18 hours before use. DMAP (98%, Fluka), H(salNdipp), $(Bu_4N)[Os(N)Cl_4]$, were dried *in vacuo* at 80 °C for at least 18 hours before use. Alumina (pH 7, Sigma Aldrich) was dried *in vacuo* at 150 °C for 36 hours.

Crystallographic studies were carried out on single crystals, which were coated with NVH oil, mounted at the end of a MiTeGen Dual-Thickness Micromount, and placed in the nitrogen cold stream of the diffractometer. Data were collected and processed using Oxford Diffraction Xcalibur Eos diffractometer (Mo K_α radiation), operated *via* CrysAlisPro software.[5] The crystal structures were solved using SHELXT (intrinsic phasing) and refined using SHELXL-2018 (least squares),[6, 7] with data processing carried out in Olex2.[8] Non-hydrogen atoms were refined anisotropically. Hydrogen atoms were placed at calculated positions and refined as riding atoms with isotropic displacement parameters (U_{iso} = 1.2 U_{eq} of the parent atom for CH groups, and U_{iso} = 1.5 U_{eq} of the parent atom for CH_3 groups). Disordered solvent was refined isotropically (toluene in **3** and in **4**), if necessary. Severely disordered solvent regions had diffuse electron density modelled with the *use solvent mask* option in Olex2 (SQUEEZE).[9] Complex **2** displays solvent-accessible voids of 493.7 $Å^3$ with an estimated electron count of 57.6 (hexane, 50 electrons). Complex **3** displays solvent-accessible voids of 714.0 $Å^3$ with an estimated electron

count of 117.6 (toluene, 56 electron). Complex **5** displays solvent-accessible voids of 1085.4 Å^3 with an estimated electron count of 226.2 (hexane, 50 electrons). Complex **6** displays solvent-accessible voids of 305.4 Å^3 with an estimated electron count of 64.6 (Et_2O, 42 electrons). Complex **7** displays solvent-accessible voids of 864.1 Å^3 with an estimated electron count of 45.4 (toluene, 56 electrons). For atoms demonstrating high disorder ISOR restraints were applied.

Elemental analyses were carried out by Mikroanalytisches Laboratorium Kolbe (Oberhausen, Germany).

IR spectroscopic studies were carried out using a Bruker ALPHA II spectrometer with solid samples measured between KBr windows or using ATR.

NMR spectroscopic studies were carried out using Bruker 400 MHz, 500 MHz, 600 MHz and 800 MHz spectrometers equipped with J. Young NMR tubes. ^{1}H and ^{13}C NMR chemical shifts are referenced to residual solvent signals (C_6D_6: ^{1}H: 7.16 ppm, ^{13}C: 128.06 ppm, THF-d_8: ^{1}H: 3.580, 1.730 ppm, ^{13}C: 67.570, 25.470 ppm). 85% H_3PO_4 in H_2O defines 0 ppm for ^{31}P, and NH_3 defines 0 ppm for ^{15}N; these frequencies are based on indirect referencing to ^{1}H.

UV-vis spectroscopic studies were carried out using an Agilent Technologies Cary 60 Spectrometer equipped with 1.00 cm quartz cuvettes sealed with Teflon stoppers and electrical tape.

Solution state magnetic susceptibility was measured by Evans' method on samples dissolved in THF-d_8 with deuterated solvent being also used as external standard inside a capillary. Correction for diamagnetism were made using tabulated Pascal constants.[10]

Direct-current (DC) magnetization measurements were conducted on polycrystalline samples, which were sealed in QuantumDesign powder capsules and analyzed using a QuantumDesign Dynacool Physical Property Measurement System (PPMS) located at the Technical University of Denmark. The magnetic susceptibility was recorded from 3 K to 300 K at 1 T using the vibrating sample magnetometry (VSM) option, while magnetization versus magnetic field measurements were carried out up to 9 T. Sample masses were determined with a Mettler-Toledo WXTSDU microbalance, and the experimental data were corrected for diamagnetic contributions.

X-Ray absorption spectroscopic studies were carried out at the ID12 beamline of the European Synchrotron (ESRF) in Grenoble, France. The X-ray absorption near edge structure (XANES) spectra of **2–5** were collected at the phosphorus K-edge and the osmium L_3- and L_2-edges at room temperature in total fluorescence yield detection mode using a Si photodiode. The incident beam was monochromatized using a Si<111> double crystal monochromator. For the osmium $L_{3,2}$-edge experiments, the second harmonics of Helios-II type undulator were used in a circular polarization mode. The intensity of the incident X-ray beam was carefully adjusted to avoid radiation damage of the sample through the insertion of attenuating Al foils. For the phosphorus K-edge experiments, the fundamental harmonics of Helios-II type undulator were used in a circular polarization mode with no attenuation. Higher-order harmonics were suppressed by a pair of B_4C mirrors installed downstream with respect to the monochromator. Additionally, energy-resolved detectors were exploited to eliminate the recording of X-ray diffraction peaks by selecting only phosphorus Kα emission lines. All spectra were normalized between zero before the absorption edge and one above the edge. The position of the step function describing transitions into the continuum was defined by following the same procedure as previously reported.[11] The osmium $L_{3,2}$-edges were corrected for reabsorption effects in order to calculate accurately the 5d-orbital populations (n_e) upon the application of spin-orbit sum rules. Transmission detection was additionally used for **5** to check the validity of the self-absorption correction used for all samples, which took into account the chemical composition, the geometry of the experiment and the solid angle of the X-ray fluorescence detector.

3 Syntheses

3.1 Synthesis of [(salNdipp)$_2$(Cl)OsN] (1)

Under a N_2 atmosphere, a solution of H(salNdipp) (98.7 mg, 0.351 mmol) in 3 ml THF was added over 5 minutes to a suspension of NaH (14.0 mg, 0.583 mmol, 1.7 eq) in 1 ml THF and stirred vigorously over 90 minutes, resulting in heavy gas evolution (for several minutes) and a slight darkening of the yellow suspension. Residual NaH was removed by filtration through celite, and the filtrate was transferred to a vial containing (Bu_4N)[Os(N)Cl_4] (103.4 mg, 0.176 mmol) and left to stir overnight, resulting in a slow color change from dark purple to orange. The solvent was removed under reduced pressure, and the residue was redissolved in a mixture of Et_2O and toluene (4.5 ml, 1:2, Et_2O:tol), filtered through celite (removing (Bu_4N)Cl and NaCl) and washed with 2x2 ml toluene. The solvent was removed under reduced pressure, leaving [(salNdipp)$_2$(Cl)OsN] (**1**) as an orange solid. Yield of [(salNdipp)$_2$(Cl)OsN] (**1**): 129.0 mg, 0.161 mmol, 91.7% based on (Bu_4N)[Os(N)Cl_4]. Crystals suitable for X-ray crystallography separated from a hexane solution of **1**, which was concentrated at –35 °C using toluene as a sorbent. **^{1}H NMR**, 400 MHz, C_6D_6 δ(ppm); 7.90 (s, 1H, Ar-C**H**-NAr), 7.86 (s, 1H, Ar-C**H**-NAr), 7.27 – 7.19 (overlapped m, 2H, Ar-C**H**), 7.19 – 7.16 (m, 1H, Ar-C**H**), 7.15 – 7.09 (overlapped m, 2H, Ar-C**H**), 7.09 – 7.00 (overlapped m, 3H, Ar-C**H**), 6.98 (ddd, *J* = 8.7, 7.0, 1.8 Hz, 1H, Ar-C**H**), 6.84 (dd, *J* = 7.9, 1.8 Hz, 1H, Ar-C**H**), 6.75 (d, *J* = 7.2 Hz, 1H, Ar-C**H**), 6.70 (d, *J* = 8.5 Hz, 1H, Ar-C**H**), 6.47 (t, *J* = 7.4 Hz, 1H, Ar-C**H**), 6.43 – 6.33 (m, 1H, Ar-C**H**), 4.63 (hept, *J* = 6.9 Hz, 1H, ^{i}Pr-C**H**(CH_3)$_2$), 4.44 (hept, *J* = 6.7 Hz, 1H, ^{i}Pr-C**H**(CH_3)$_2$), 4.16 (hept, *J* = 6.8 Hz, 1H, ^{i}Pr-C**H**(CH_3)$_2$), 3.15 (hept, *J* = 6.6 Hz, 1H, ^{i}Pr-C**H**(CH_3)$_2$), 1.49 (d, *J* = 6.7 Hz, 3H, ^{i}Pr-CH(C**H**$_3$)$_2$), 1.46 (d, *J* = 6.7 Hz, 3H, ^{i}Pr-CH(C**H**$_3$)$_2$), 1.40 (d, *J* = 6.7 Hz, 3H, ^{i}Pr-CH(C**H**$_3$)$_2$), 1.20 (d, *J* = 7.2 Hz, 3H, ^{i}Pr-CH(C**H**$_3$)$_2$), 1.19 (d, *J* = 7.2 Hz, 3H, ^{i}Pr-CH(C**H**$_3$)$_2$), 1.15 (d, *J* = 6.7 Hz, 3H, ^{i}Pr-CH(C**H**$_3$)$_2$), 1.00 (d, *J* = 6.8 Hz, 3H, ^{i}Pr-CH(C**H**$_3$)$_2$), 0.90 (d, *J* = 6.6 Hz, 3H, ^{i}Pr-CH(C**H**$_3$)$_2$). **^{13}C{^{1}H} NMR**, 126 MHz, C_6D_6 δ(ppm): 172.19, 171.78, 168.67, 166.27, 156.35, 148.13, 144.92, 142.64, 142.64 (*HMBC reveals 2 overlapping peaks at 142.64 ppm*), 141.83, 138.76, 137.58, 137.04, 136.01, 128.65, 128.64 (*HSQC reveals 2 overlapping peaks at 128.65 and 128.64 ppm*), 124.92, 124.29, 124.18, 123.93, 122.11, 121.24, 120.02, 118.48, 117.97, 116.13, 28.21, 27.85, 27.85 (*HSQC reveals 2 overlapping peaks at 27.85 ppm)* 27.57, 26.41, 25.96, 25.64, 24.84, 24.12, 23.92, 23.64, 23.45. **Elemental analysis**, calculated for $C_{38}H_{44}N_3O_2OsCl$: C: 57.02%, H: 5.54%, N: 5.25%; found: C: 56.87%, H: 5.45%, N: 5.21%.

*Note: If $(Bu_4N)Cl$ remains in the sample of **1**, redissolving the complex in a 1:1 mixture of hexane and Et_2O and stirring with alumina (10:1 by mass, pH 7) for 1 hour and filtration through alumina yields pure [(salNdipp)$_2$(Cl)OsN].*

*Note on stability: **1** is stable when dissolved in a sealed tube (J Young) in C_6D_6 at 80 °C for 18 hours under N_2. **1** is stable in air at room temperature.*

3.2 Synthesis of [(salNdipp)$_2$(OTf)OsN] (2)

Under a N_2 atmosphere, a solution of AgOTf (53.8 mg, 0.209 mmol) in 1 ml toluene was added to a solution of [(salNdipp)$_2$(Cl)OsN] (**1,** 153 mg, 0.191 mmol) in 2 ml THF under heavy stirring. After 30 minutes the suspension was filtered through celite (removing AgCl), and the solvents were removed under reduced pressure, leaving [(salNdipp)$_2$(OTf)OsN] (**2**) as an orange crystalline material. Yield of [(salNdipp)$_2$(OTf)OsN] · THF (**7**): 180 mg, 0.183 mmol, 95.5% based on **1**. Crystals suitable for X-ray crystallography separated from a hexane solution of **2**, which was concentrated at –35 °C using toluene as a sorbent. **^{1}H NMR**, 500 MHz, C_6D_6, δ (ppm): 8.00 (s, 2H, Ar-C**H**-NAr), 7.43 (dd, J = 7.8, 1.5 Hz, 2H, Ar-C**H**), 7.30 (t, J = 7.8 Hz, 2H, Ar-C**H**), 7.10 – 7.03 (overlapped m, 4H, Ar-C**H**), 7.03 – 6.96 (m, 2H, Ar-C**H**), 6.77 (dd, J = 7.9, 1.7 Hz, 2H, Ar-C**H**), 6.38 (ddd, J = 8.0, 6.7, 1.4 Hz, 2H, Ar-C**H**), 4.00 (hept, J = 6.6 Hz, 2H, ^{i}Pr-C**H**(CH$_3$)$_2$), 2.77 (hept, J = 6.7 Hz, 2H, ^{i}Pr-C**H**(CH$_3$)$_2$), 1.72 (d, J = 6.6 Hz, 6H, ^{i}Pr-CH(C**H**$_3$)$_2$), 1.31 (d, J = 6.6 Hz, 6H, ^{i}Pr-CH(C**H**$_3$)$_2$), 1.02 (d, J = 6.7 Hz, 6H, ^{i}Pr-CH(C**H**$_3$)$_2$), 0.81 (d, J = 6.7 Hz, 6H, ^{i}Pr-CH(C**H**$_3$)$_2$). **^{13}C{^{1}H} NMR**, 101 MHz, C_6D_6, δ (ppm): 173.46, 167.40, 146.38, 146.19, 143.30, 139.20, 136.96, 129.34, 124.52, 123.76, 122.26, 119.86, 118.83, 28.79, 27.89, 27.26, 26.02, 22.71, 22.59. **^{19}F NMR**, 376 MHz, C_6D_6, δ (ppm): –78.17. **UV/Vis**, THF, λ [nm, ε (max/sh, M^{-1} cm^{-1})]: 299 (max, 18700), 391 (max, 4300). **Elemental analysis**, calculated for $C_{39}H_{44}F_3N_3O_5OsS$: C: 51.25%, H: 4.85%, N: 4.60%; found: C: 51.00%, H: 4.81%, N: 4.54%.

*Note on stability: **2** is stable when dissolved in a sealed tube (J Young) in C_6D_6 at 80 °C for 18 hours under N_2. **2** decomposes in air.*

3.3 Reaction of [(salNdipp)$_2$(OTf)OsN] (2) with Na(OCP)

Under a N_2 atmosphere, [(salNdipp)$_2$(OTf)OsN] (**2**, 10.6 mg, 11.6 µmol) and Na(OCP) · 2.5 dioxane (3.6 mg, 12 µmol) were dissolved in 1 ml THF, resulting in an instantaneous darkening

of the orange solution. The reaction mixture was left standing for 1 hour and filtered through celite. The solvent was removed under reduced pressure and the products were analyzed by $^{31}P\{^1H\}$ NMR (**Supplementary Fig. 41**) and IR (**Supplementary Fig. 33**). Spectroscopic data: **^{31}P {^{1}H}NMR** (162 MHz, C_6D_6) δ (ppm): 232.76, 219.53. **IR** (cm^{-1}): 2202, 2151, 2112, 2024, 1952, 1909, 1897.

3.4 Synthesis of [(salNdipp)$_2$(DMAP)Os(NP)] (3)

Method A (one pot from (Bu_4N)[Os(N)Cl_4].

Under a N_2 atmosphere, a solution of H(salNdipp) (956.3 mg, 3.40 mmol) in 9 ml THF was added over 5 minutes to a suspension of NaH (122.3 mg, 5.10 mmol, 1.5 eq) in 1 ml THF and stirred vigorously over 3 hours, resulting in heavy gas evolution (for several minutes) and a slight darkening of the yellow suspension. Residual NaH was removed by filtration through celite, and the filtrate was transferred to a flask containing (Bu_4N)[Os(N)Cl_4] (1.00 g, 1.70 mmol) and left to stir for 46 hours, resulting in a slow color change from dark purple to orange. The solvent was removed under reduced pressure, and the residue was redissolved in a mixture of Et_2O and toluene (30 ml, 1:2, Et_2O:tol), filtered through celite (removing (Bu_4N)Cl and NaCl) and washed through the celite with 2x10 ml toluene. The solvent was removed under reduced pressure, leaving [(salNdipp)$_2$(Cl)OsN] (**1**) as an orange solid. The solid was redissolved in 10 ml THF and a solution of AgOTf (436.5 mg, 1.70 mmol) in 6 ml toluene was added under heavy stirring. After 2.5 hours, the suspension was filtered through celite (removing AgCl), and the solvents were removed under reduced pressure, leaving [(salNdipp)$_2$(OTf)OsN] (**2**) as an orange crystalline material. The resulting solid and 4-(dimethylamino)pyridine (DMAP, 206.0 mg, 1.69 mmol) were dissolved in 30 ml toluene, and the reaction mixture was left to stir for 40 hours, resulting in an orange suspension. The solvent was removed under reduced pressure. To the solid, Na(OCP) · 2.5 dioxane (562.0 mg, 1.86 mmol, 1.1 eq.) in 50 ml THF was added, resulting in gas evolution (CO, for several minutes) and a color change from light orange to dark orange. The reaction mixture was left overnight before being filtered through celite and the solvent was removed under reduced pressure. The solid was then redissolved in a minimal amount of toluene (ca. 400 ml), and Al_2O_3 (pH 7, 17 g) was added to adsorb NaOTf (this the byproduct forms a THF adduct with similar solubility to **3**). The mixture was stirred overnight, the solution was filtered through celite, and the solvent was removed under reduced pressure. The solid residue was then redissolved in minimal THF

(ca. 40 ml) and diluted with hexane (ca. 460 ml), until the solution started to turn cloudy. The solution was then swirled and left at -35 °C overnight to grow dark orange crystals of [(salNdipp)$_2$(DMAP)Os(NP)] (**3**). The mother liquor was removed by decanting, and the very dark orange crystals were washed with cold hexane 3x5 ml and dried under reduced pressure. Yield of [(salNdipp)$_2$(DMAP)Os(NP)] (**3**): 1072 mg, 1.17 mmol, 68.7% based on (Bu$_4$N)[Os(N)Cl$_4$].

Method B (from (Bu$_4$N)[Os(^{15}N)Cl$_4$].

Under a N_2 atmosphere, a solution of H(salNdipp) (116.2 mg, 0.413 mmol) in 3 ml THF was added over 5 minutes to a suspension of NaH (14.0 mg, 0.583 mmol, 1.4 eq) in 1 ml THF and stirred vigorously over 90 minutes, resulting in heavy gas evolution (for several minutes) and a slight darkening of the yellow suspension. Residual NaH was removed by filtration through celite, and the filtrate was transferred to a vial containing (Bu$_4$N)[Os(^{15}N)Cl$_4$] (121.0 mg, 0.205 mmol) and left to stir overnight, resulting in a slow color change from dark purple to orange. The solvent was removed under reduced pressure, and the residue was redissolved in a mixture of Et$_2$O and toluene (15 ml, 1:2, Et$_2$O:tol), filtered through celite (removing (Bu$_4$N)Cl and NaCl) and washed through with 2x2 ml toluene. The solvent was removed under reduced pressure, leaving [(salNdipp)$_2$(Cl)Os^{15}N] (**1**-^{15}N) as an orange solid. The solid was redissolved in 5 ml THF and a solution of AgOTf (53.8 mg, 0.209 mmol) in 3 ml toluene was added under heavy stirring. After 30 minutes the suspension was filtered through celite (removing AgCl), and the solvents were removed under reduced pressure, leaving [(salNdipp)$_2$(OTf)Os^{15}N] (**2**-^{15}N) as an orange crystalline material. The resulting solid and 4-(dimethylamino)pyridine (DMAP, 25.0 mg, 0.205 mmol) were dissolved in 15 ml toluene, and the reaction mixture was left to stir overnight, resulting in an orange suspension. The solvent was removed under reduced pressure. To the solid Na(OCP) · 2.5 dioxane (68.9 mg, 0.228 mmol, 1.1 eq) in 5 ml THF was added, resulting in gas evolution (CO, for several minutes) and a color change from light orange to dark orange. After 30 minutes the reaction mixture was filtered through celite and the solvent was removed under reduced pressure. The solid was then redissolved in a minimal amount of toluene (ca. 30 ml), and Al_2O_3 (pH 7, 2 g) was added to adsorb NaOTf (this byproduct forms a THF adduct with similar solubility to **3**). The mixture was stirred for 2 hours, the solution was filtered through celite, and the solvent was removed under reduced pressure. The solid residue was then redissolved in minimal THF (ca. 5 ml) and diluted with hexane (ca 50 ml), until the solution started to turn cloudy. The solution was then swirled and left at -35 °C overnight to

grow dark orange crystals of [(salNdipp)$_2$(DMAP)Os(^{15}NP)] (**3**-^{15}N). The mother liquor was removed by decanting, and the very dark orange crystals were washed with cold hexane 3x2 ml and dried under reduced pressure. Yield of [(salNdipp)$_2$(DMAP)Os(^{15}NP)] (**3-^{15}N**): 177.6 mg, 0.193 mmol, 94.1% based on (Bu_4N)[Os(^{15}N)Cl_4].

Characterization data for 3. Crystals suitable for X-ray crystallography separated from a hexane solution of **3**, which was concentrated at –35 °C using toluene as a sorbent. **^{1}H NMR**, (500 MHz, C_6D_6) δ: 8.22 (s, 1H, Ar-C**H**-NAr), 8.21 (s, 1H, Ar-C**H**-NAr), 7.78 (dd, *J* = 6.9, 1.2 Hz, 1H, DMAP-C**H**), 7.52 (dd, *J* = 6.9, 1.1 Hz, 1H, DMAP-C**H**), 7.42 (dd, *J* = 7.7, 1.5 Hz, 1H, Ar-C**H**), 7.22-7.16 (overlapped m, 3H, Ar-C**H**), 7.16-09 (overlapped m, 2H, Ar-C**H**), 7.09-7.06 (overlapped m, 3H, Ar-C**H**), 7.03 (dd, *J* = 8.5, 1.1 Hz, 1H, Ar-C**H**), 6.96 (t, *J* = 7.7 Hz, 1H, Ar-C**H**), 6.76 (dd, *J* = 7.7, 1.5 Hz, 1H, Ar-C**H**), 6.58 (ddd, *J* = 8.0, 6.9, 1.2 Hz, 1H, Ar-C**H**), 6.45 (ddd, *J* = 7.9, 6.7, 1.2 Hz, 1H, Ar-C**H**), 5.42 (dd, *J* = 7.0, 3.2 Hz, 1H, DMAP-C**H**), 5.13 (dd, *J* = 7.0, 3.2 Hz, 1H, DMAP-C**H**), 4.96 (hept, *J* = 6.8 Hz, 1H, ^{i}Pr-C**H**(CH_3)$_2$), 4.43 (hept, *J* = 6.7 Hz, 1H, ^{i}Pr-C**H**(CH_3)$_2$), 4.07 (hept, *J* = 6.7 Hz, 1H, ^{i}Pr-C**H**(CH_3)$_2$), 2.00 (s, 6H, DMAP-C**H**$_3$), 1.94 (hept, *J* = 6.6 Hz, 1H, ^{i}Pr-C**H**(CH_3)$_2$), 1.68 (d, *J* = 6.6 Hz, 3H, ^{i}Pr-CH(C**H**$_3$)$_2$), 1.53 (d, *J* = 6.7 Hz, 3H, ^{i}Pr-CH(C**H**$_3$)$_2$), 1.38 (d, *J* = 6.9 Hz, 3H, ^{i}Pr-CH(C**H**$_3$)$_2$), 1.18 (d, *J* = 6.7 Hz, 3H, ^{i}Pr-CH(C**H**$_3$)$_2$), 0.94 (two overlapped d, *J* = 6.7, 1.3 Hz, 6H, ^{i}Pr-CH(C**H**$_3$)$_2$), 0.86 (d, *J* = 6.8 Hz, 3H, ^{i}Pr-CH(C**H**$_3$)$_2$), 0.59 (d, *J* = 6.8 Hz, 3H, ^{i}Pr-CH(C**H**$_3$)$_2$). **^{13}C{^{1}H} NMR**, 126 MHz, C_6D_6, δ (ppm): 168.85, 166.49, 164.96, 164.84, 152.94, 152.13, 151.92, 150.66, 148.11, 144.85, 143.91, 143.35, 140.58, 135.53, 135.30, 133.88, 133.43, 126.77, 126.57, 124.58, 123.57, 123.41, 123.35, 122.97, 122.71, 121.94, 120.45, 115.22, 114.40, 107.27, 107.02, 38.30, 27.82, 27.74, 26.94, 26.56, 26.30, 26.26, 26.10, 25.95, 25.39, 25.24, 23.16, 22.77. **^{31}P{^{1}H} NMR**, C_6D_6, δ (ppm): 249.31 (**3**, 162 MHz), 249.02 (**3-^{15}N**, *J* = 61.8 Hz, 243 MHz). **^{15}N{^{1}H} NMR**, 81.11 MHz, C_6D_6, δ (ppm): 395.97 (*J* = 62.0 Hz). **IR**, solid between KBr windows, *ν* (cm^{-1}): 1258/1221 (P≡N) for **3/3-^{15}N**. **UV/Vis**, THF, *λ* [nm, *ε* (max/sh, M^{-1} cm^{-1})]: 258 (max, 44900), 318 (max, 25500), 348 (max, 26500), 401 (max, 18000), 572 (sh, 2000). **Elemental analysis**, calculated for $C_{45}H_{54}N_5O_2OsP$: C: 58.87%, H: 5.93%, N: 7.63%; found: C: 58.86%, H: 5.91%, N: 7.61%.

*Note on stability: **3** is stable when dissolved in a sealed tube (J Young) in C_6D_6 at 120 °C for 24 hours under N_2. **3** hydrolyses completely in 18 hours under N_2 at room temperature. **3** decomposes in air.*

3.5 Synthesis of [(salNdipp)$_2$(DMAP)Os(NPS$_2$)] (4)

Under a N_2 atmosphere, [(salNdipp)$_2$(DMAP)Os(NP)] (**3,** 25.0 mg, 27.2 µmol) and S_8 (1.74 mg, 54.3 µmol of S, 2.0 eq.)* were dissolved in 7.5 ml toluene, resulting in a slow color change from dark orange to dark green. The reaction mixture was cooled to -35 °C overnight, resulting in crystallization of [(salNdipp)$_2$(DMAP)Os(NPS$_2$)] (**4**) as dark orange crystals. The solution was decanted, and the dark orange crystals were washed with cold toluene (3x1 ml), and dried under reduced pressure. Yield of [(salNdipp)$_2$(DMAP)Os(NPS$_2$)] (**4**), 19.5 mg, 19.9 µmol 72.9% based on **3**. Crystals suitable for X-ray crystallography separated from the reaction mixture of [(salNdipp)$_2$(DMAP)Os(NPS$_2$)] (**4**). **^{1}H NMR**, 600 MHz, THF-d$_8$, δ (ppm): 7.50 (dd, *J* = 7.8, 1.8 Hz, 1H, Ar-C**H**), 7.38 (t, *J* = 7.7 Hz, 1H, Ar-C**H**), 7.33 (dd, *J* = 7.8, 1.5 Hz, 1H, Ar-C**H**), 7.21 (dd, *J* = 7.9, 1.8 Hz, 1H, Ar-C**H**), 7.20 (dd, *J* = 7.7, 1.6 Hz, 1H, Ar-C**H**), 7.09 (s, 1H, Ar-C**H**-NAr), 7.08 (t, *J* = 7.8 Hz, 1H, Ar-C**H**), 7.02 (ddd, *J* = 8.6, 7.0, 1.8 Hz, 1H, Ar-C**H**), 6.94 (dd, *J* = 7.8, 1.5 Hz, 1H, Ar-C**H**), 6.92 (dd, *J* = 8.6, 1.1 Hz, 1H, Ar-C**H**), 6.89 (dd, *J* = 7.1, 1.3 Hz, 1H, DMAP-C**H**), 6.86 (ddd, *J* = 7.9, 6.8, 1.1 Hz, 1H, Ar-C**H**), 6.73 (dd, *J* = 7.8, 1.5 Hz, 1H, Ar-C**H**), 6.65 (dd, *J* = 7.1, 3.2 Hz, 1H, DMAP-C**H**), 6.53 (dd, *J* = 7.1, 1.3 Hz, 1H, DMAP-C**H**), 6.46 (ddd, *J* = 8.6, 6.9, 1.8 Hz, 1H, Ar-C**H**), 6.22 (ddd, *J* = 7.9, 7.0, 1.1 Hz, 1H, Ar-C**H**), 6.18 (dd, *J* = 7.1, 3.2 Hz, 1H, DMAP-C**H**), 6.06 (dd, *J* = 8.4, 1.0 Hz, 1H, Ar-C**H**), 4.93 (s, 1H, Ar-C**H**-NAr), 4.49 (hept, *J* = 6.7 Hz, 1H, ^{i}Pr-C**H**(CH$_3$)$_2$), 3.90 (hept, *J* = 6.7 Hz, 1H, ^{i}Pr-C**H**(CH$_3$)$_2$), 3.87 (hept, *J* = 6.7 Hz, 1H, ^{i}Pr-C**H**(CH$_3$)$_2$), 3.14 (hept, *J* = 6.3 Hz, 1H, ^{i}Pr-C**H**(CH$_3$)$_2$), 3.08 (s, 6H, DMAP-C**H**$_3$), 1.34 (d, *J* = 6.8 Hz, 3H, ^{i}Pr-CH(C**H**$_3$)$_2$), 1.25 (d, *J* = 6.6 Hz, 3H, ^{i}Pr-CH(C**H**$_3$)$_2$), 1.24 (d, *J* = 6.7 Hz, 3H, ^{i}Pr-CH(C**H**$_3$)$_2$), 1.18 (d, *J* = 6.7 Hz, 3H, ^{i}Pr-CH(C**H**$_3$)$_2$), 1.07 (d, *J* = 6.7 Hz, 3H, ^{i}Pr-CH(C**H**$_3$)$_2$), 1.01 (d, *J* = 6.7 Hz, 3H, ^{i}Pr-CH(C**H**$_3$)$_2$), 0.89 (d, *J* = 6.7 Hz, 3H, ^{i}Pr-CH(C**H**$_3$)$_2$), 0.83 (d, *J* = 6.8 Hz, 3H, ^{i}Pr-CH(C**H**$_3$)$_2$). **^{13}C NMR,** (151 MHz, THF) δ (ppm): 201.41, 188.72, 184.76, 179.01, 160.01, 156.23, 154.32, 153.95, 150.47, 146.20, 143.65, 142.46, 142.00, 141.98, 140.23, 136.77, 134.92, 128.25, 127.60, 127.60 (*HSQC reveals 2 overlapping peaks at 127.60 ppm*), 127.25, 124.95, 124.79, 121.65, 120.83, 118.66, 112.77, 111.76, 107.89, 106.00, 105.90, 38.63, 35.93, 29.76, 28.88, 28.02, 27.21, 27.21 (*HSQC reveals 2 overlapping peaks at 27.21 ppm*), 26.72, 26.23, 23.49, 22.97, 22.88, 22.48. **^{31}P{^{1}H} NMR**, 243 MHz, THF-d$_8$, δ (ppm): 233.13 (**4**), 232.64 (**4-^{15}N**, d, *J* = 50.6 Hz). **^{1}H-^{15}N HMBC NMR**, 61 MHz, THF-d$_8$, δ (ppm): 954.53 (d, *J* = 50.5 Hz). **UV/Vis**, THF, λ [nm, ε (max/sh, M^{-1} cm^{-1})]: 308 (max, 29000), 382 (max, 28000), 440 (sh, 12000), 512 (max,

5900), 580 (max, 4800). **Elemental analysis**, calculated for $C_{45}H_{54}N_5O_2OsPS_2$: C: 55.02%, H: 5.54%, N: 7.13%; found: C: 54.74%, H: 5.59%, N: 7.05%.

** Note: Sulfur was weighed precisely by making a stock solution of 17.4 mg S_8 in 10 ml toluene and taking out a 1 ml aliquot.*
*Notes on the reactivity of **4**: Sulfur atom exchange between **4** and **3** is described in Section **7.2**, and desulfurization of **4** with PPh_3 to form **3** and $SPPh_3$ is described in Section **7.3**.*
*Note on stability: **4** is stable when dissolved in a sealed tube (J Young) in C_6D_6 at 80 °C for 18 hours under N_2. **4** decomposes in air.*

3.6 Synthesis of [(salNdipp)$_2$(DMAP)Os(NPCl)] (5)

Under a N_2 atmosphere, [(salNdipp)$_2$(DMAP)Os(NP)] (**3,** 100.0 mg, 0.109 mmol) and Ph_3CCl (40.5 mg, 0.145 mmol, 1.3 eq.) were dissolved in 5 ml THF, and the reaction mixture was left for 3 hours. The solvent was removed under reduced pressure, and the dark orange residue was redissolved in minimum amount of THF (3 ml), and diluted with hexane (15 ml), cooled to -35 °C and left to crystallize overnight. The solution was decanted and dark orange crystals of [(salNdipp)$_2$(DMAP)Os(NPCl)] (**5**) were washed with cold hexane (3x2 ml), and dried under reduced pressure. Yield of [(salNdipp)$_2$(DMAP)Os(NPCl)] (**5**): 100.7 mg, 0.106 mmol, 97.0% based on **3**. Crystals suitable for X-ray crystallography separated from a THF solution of [(salNdipp)$_2$(DMAP)Os(NPCl)] (**5**), with hexane/toluene as sorbent at -35 °C. **^{1}H NMR** (400 MHz, C_6D_6) δ 23.31 (FWHM = 390 Hz), 19.95 (FWHM = 190 Hz), 18.69 (FWHM = 200 Hz), 18.11 (FWHM = 130 Hz), 16.03 (FWHM = 120 Hz), 10.23 (FWHM = 120 Hz), 10.05 (FWHM = 60 Hz), 9.20 (FWHM = 50 Hz), 8.31 (FWHM = 40 Hz), 7.38 (FWHM = 20 Hz), 7.35 (FWHM = 5 Hz), 6.95 (FWHM = 10 Hz), 5.33 (FWHM = 30 Hz), 3.83 (FWHM = 140 Hz), 3.62 (FWHM = 30 Hz), 3.13 (FWHM = 90 Hz), 1.93 (FWHM = 60 Hz), 0.56 (FWHM = 70 Hz), -0.03 (FWHM = 60 Hz), -0.60 (FWHM = 90 Hz), -2.86 (FWHM = 50 Hz), -3.48 (FWHM = 60 Hz), -7.48 (FWHM = 300 Hz), -10.64 (FWHM = 350 Hz), -22.24 (FWHM = 1100 Hz). **UV/Vis**, THF, λ [nm, ε (max/sh, M^{-1} cm^{-1})]: 256 (max, 29000), 337 (max, 17000), 395 (max, 13000). **Magnetic moment**, μ_{eff} (Evans' method, THF-d_8, 298 K): 1.83 μ_B. **Elemental analysis**, calculated for $C_{45}H_{54}ClN_5O_2OsP$: C: 56.68%, H: 5.71%, N: 7.34%; found: C: 56.59%, H: 5.74%, N: 7.31%.

Note on stability: ***5*** *is stable when dissolved in a sealed tube (J Young) in C_6D_6 at 80 °C for 18 hours under N_2.* ***5*** *decomposes in air.*

3.7 Synthesis of [(salNdipp)$_2$(DMAP)Os(N_4P)] (6)

Under a N_2 atmosphere, [(salNdipp)$_2$(DMAP)Os(NPCl)] (**5,** 25.0 mg, 26.2 µmol) and Me_3SiN_3 (3.44 mg, 29.8 µmol, 1.15 eq) were dissolved in 1.5 ml THF, and the reaction mixture was left for 1 hour. The solution was diluted with hexane (ca. 10 ml) to precipitate out dark orange crystals of [(salNdipp)$_2$(DMAP)Os(N_4P)] (**6**), the mixture was cooled to -35 °C for 30 minutes and washed with 3x1 ml hexane. Yield of [(salNdipp)$_2$(DMAP)Os(N_4P)] (**6**), 13.9 mg, 14.5 µmol, 55.2% based on **5**. Crystals suitable for X-ray crystallography separated from a Et_2O solution of [(salNdipp)$_2$(DMAP)Os(N_4P)] (**6**), with toluene as sorbent at -35 °C. **^{1}H NMR** (400 MHz, C_6D_6) δ 18.87 (FWHM = 80 Hz), 17.16 (FWHM = 100 Hz), 17.09 (FWHM = 60 Hz), 14.62 (FWHM = 100 Hz), 13.60 (FWHM = 40 Hz), 12.36 (FWHM = 40 Hz), 10.99 (FWHM = 40 Hz), 9.08 (FWHM = 70 Hz), 8.29 (FWHM = 20 Hz), 7.65 (FWHM = 20 Hz), 7.63 (FWHM = 20 Hz), 6.63 (FWHM = 20 Hz), 5.65 (FWHM = 60 Hz), 5.35 (FWHM = 70 Hz), 3.75 (FWHM = 30 Hz), 3.00 (FWHM = 40 Hz), 1.76 (FWHM = 60 Hz), 0.97 (FWHM = 20 Hz), -1.04 (FWHM = 110 Hz), -1.81 (FWHM = 50 Hz), -5.60 (FWHM = 40 Hz), -5.76 (FWHM = 30 Hz), -6.38 (FWHM = 200 Hz), -14.01 (FWHM = 550 Hz), -19.64 (FWHM = 270 Hz), -23.41 (FWHM = 500 Hz), -30.42 (FWHM = 1500 Hz). **UV/Vis**, THF, λ [nm, ε (max/sh, M^{-1} cm^{-1})]: 320 (max, 19000), 390 (max, 12000), 592 (max, 1500). **Magnetic moment**, μ_{eff} (Evans' method, THF-d_8, 298 K): 1.85 μ_B. **Elemental analysis**, calculated for $C_{45}H_{54}N_8O_2OsP$: C: 56.29%, H: 5.67, N: 11.67; found: C: 56.19%, H: 5.65%, N: 11.64%.

Note: other azide sources also convert ***5*** *to* ***6****, but due to the low stability of the product, these methods do not allow a pure product to be isolated.* ***[1]*** *When using (Bu_4N)(N_3) in C_6D_6,* ***6*** *forms cleanly within 5 minutes, but the (Bu_4N)Cl byproduct has a similar solubility to* ***6****; attempts at removing (Bu_4N)Cl with alumina resulted in full decomposition of* ***6****.* ***[2]*** *When using NaN_3 in THF or dioxane, the formation of* ***6*** *is so slow that its thermal decomposition to* ***3*** *and N_2 complex* ***7*** *(see section* ***3.9****) prevents isolation of a pure product.* ***[3]*** *When using NaN_3 with LiCl as phase-transfer catalyst in THF, the conversion rate is variable, and lithium ion remains in the sample, as verified from ^{7}Li NMR.*

*Note on stability: In the solid state, **6** is unstable at -35 °C and slowly decomposes over the course of weeks. **6** fully decomposes when dissolved in a sealed tube (J Young) in C_6D_6 at 50 °C for 18 hours under N_2. **6** decomposes in air.*

3.8 Reduction of [(salNdipp)$_2$(DMAP)Os(N_4P)] (6) with KC_8

Under a N_2 atmosphere, [(salNdipp)$_2$(DMAP)Os(NPCl)] (**5,** 13.3 mg, 13.9 µmol) and (Bu_4N)(N_3) (2.8 mg, 9.8 µmol, 0.7 eq.) were dissolved in 4 ml THF, and the reaction mixture was left for 1.5 hour. The reaction mixture was cooled to -35 °C and added to KC_8 (1.8 mg, 13.3 µmol) and left to stir overnight. The solvent was removed under reduced pressure yielding a mixture of solids including graphite, [(salNdipp)$_2$(DMAP)Os(NP)] (**3**) and KN_3. The products were identified by ^{31}P and ^{1}H NMR (**3**) and IR spectroscopy (KN_3, **Supplementary Fig. 32**) respectively.

3.9 Thermal decomposition of [(salNdipp)$_2$(DMAP)Os(N_4P)] (6) to form [(salNdipp)$_2$(DMAP)Os(NP)] (3) and [(salNdipp)$_2$(DMAP)Os(N_2)] (7).

Under a N_2 atmosphere a solution of [(salNdipp)$_2$(DMAP)Os(N_4P)] (**6,** 40.0 mg, 0.042 mmol) in 5 ml toluene was heated to 50 °C overnight. The reaction mixture was filtered through tissue paper (Kimwipe) and the solvent was removed under reduced pressure yielding [(salNdipp)$_2$(DMAP)Os(NP)] (**3**) and [(salNdipp)$_2$(DMAP)Os(N_2)] (**7**) in a 1:1 distribution according to ^{1}H NMR (**Supplementary Fig. 46**). A white amorphous solid was observed in the solution and collected on the filter and characterized by IR spectroscopy (**Supplementary Fig. 34**). [*In attempted crystallization experiments, it was found that **3** and **7** co-crystallize, which prevents direct identification of the dinitrogen complex by X-ray crystallography*]. To isolate the N_2 complex [(salNdipp)$_2$(DMAP)Os(N_2)] (**7**), sulfur (1.35 mg, 0.042 mmol of S) in 8 ml toluene was added to selectively convert [(salNdipp)$_2$(DMAP)Os(NP)] (**3**) into [(salNdipp)$_2$(DMAP)Os(NPS_2)] (**4**) while leaving **7** unreacted (**Supplementary Fig. 46.**). The solvent was removed under reduced pressure, and the mixture was redissolved in Et_2O, which was concentrated at –35 °C using toluene as a sorbent. This yielded single crystals of [(salNdipp)$_2$(DMAP)Os(N_2)] (**7**), which were characterized by X-ray crystallography.

4 NMR Spectroscopy

4.1 NMR Spectral Data for [(salNdipp)$_2$(Cl)OsN] (1)

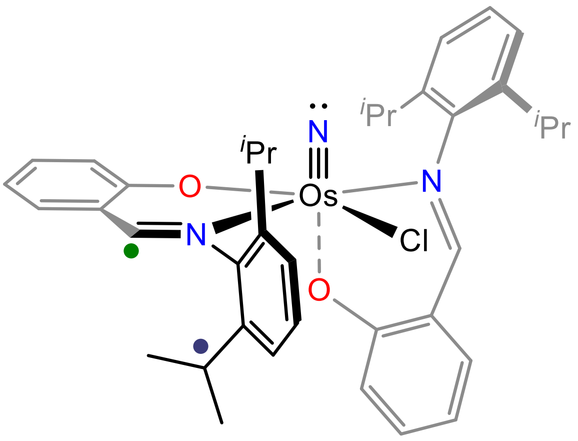

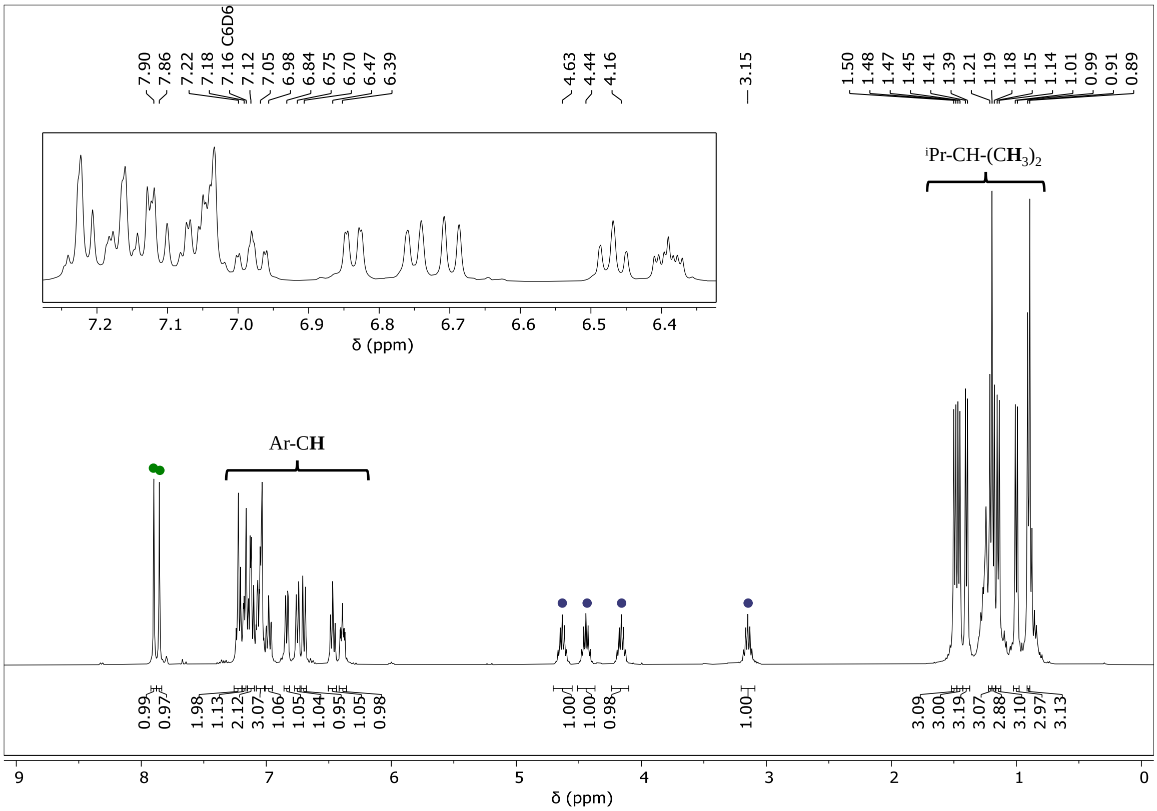

Supplementary Fig. 1. ^{1}H NMR spectrum of [(salNdipp)$_2$(Cl)OsN] (**1**) in C_6D_6 (500 MHz).

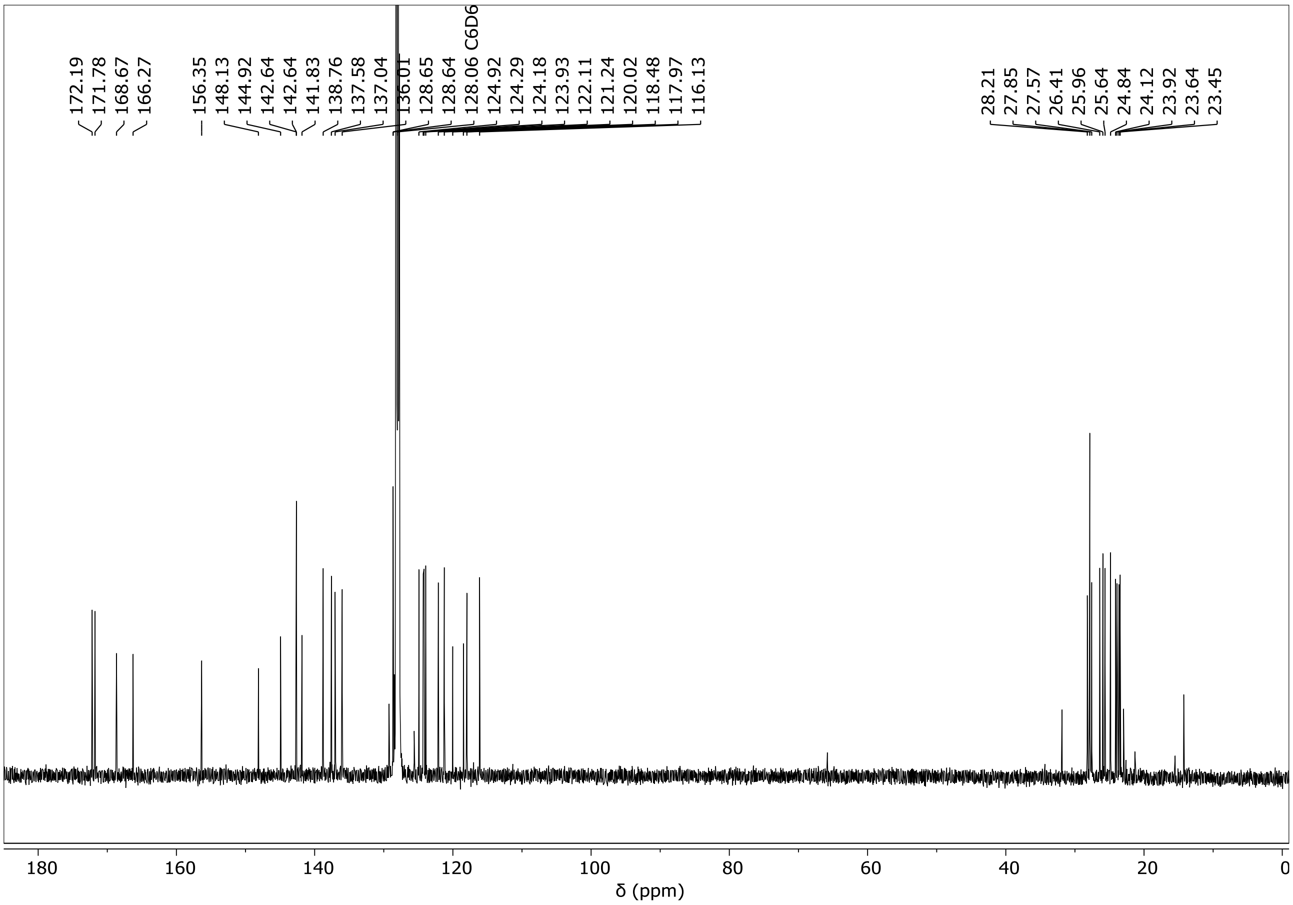

Supplementary Fig. 2. $^{13}C\{^{1}H\}$ NMR spectrum of [(salNdipp)$_2$(Cl)OsN] (**1**) in C_6D_6 (126 MHz). The resonances at 137.53, 128.98, 128.21, and 21.07 ppm (toluene), 65.56 and 15.24 ppm (Et_2O) and 33.61, 23.70 and 14.00 ppm (hexane) arise from traces of solvent.

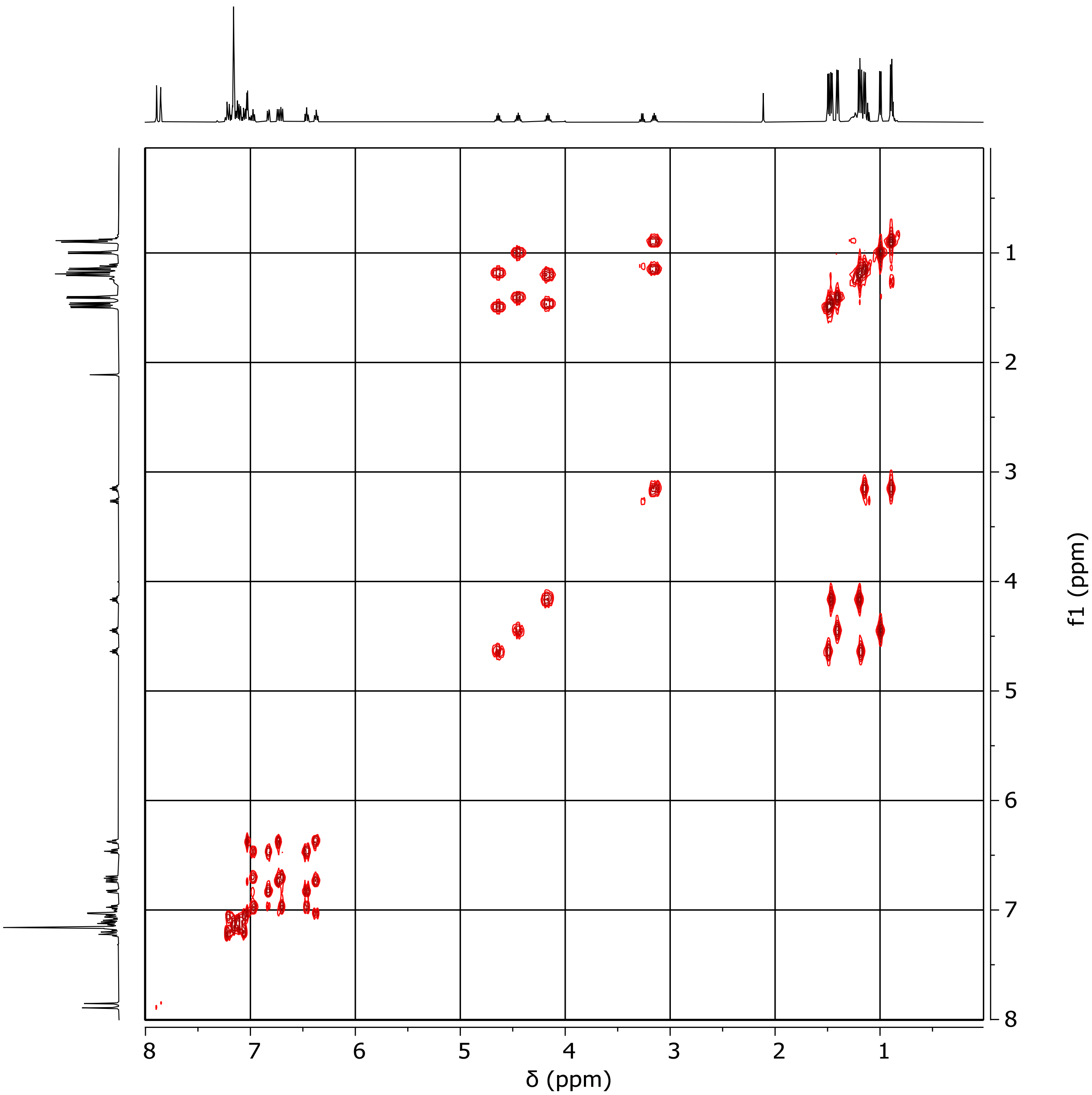

Supplementary Fig. 3 COSY NMR spectrum of [(salNdipp)$_2$(Cl)OsN] (**1**) in C_6D_6 (500 MHz).

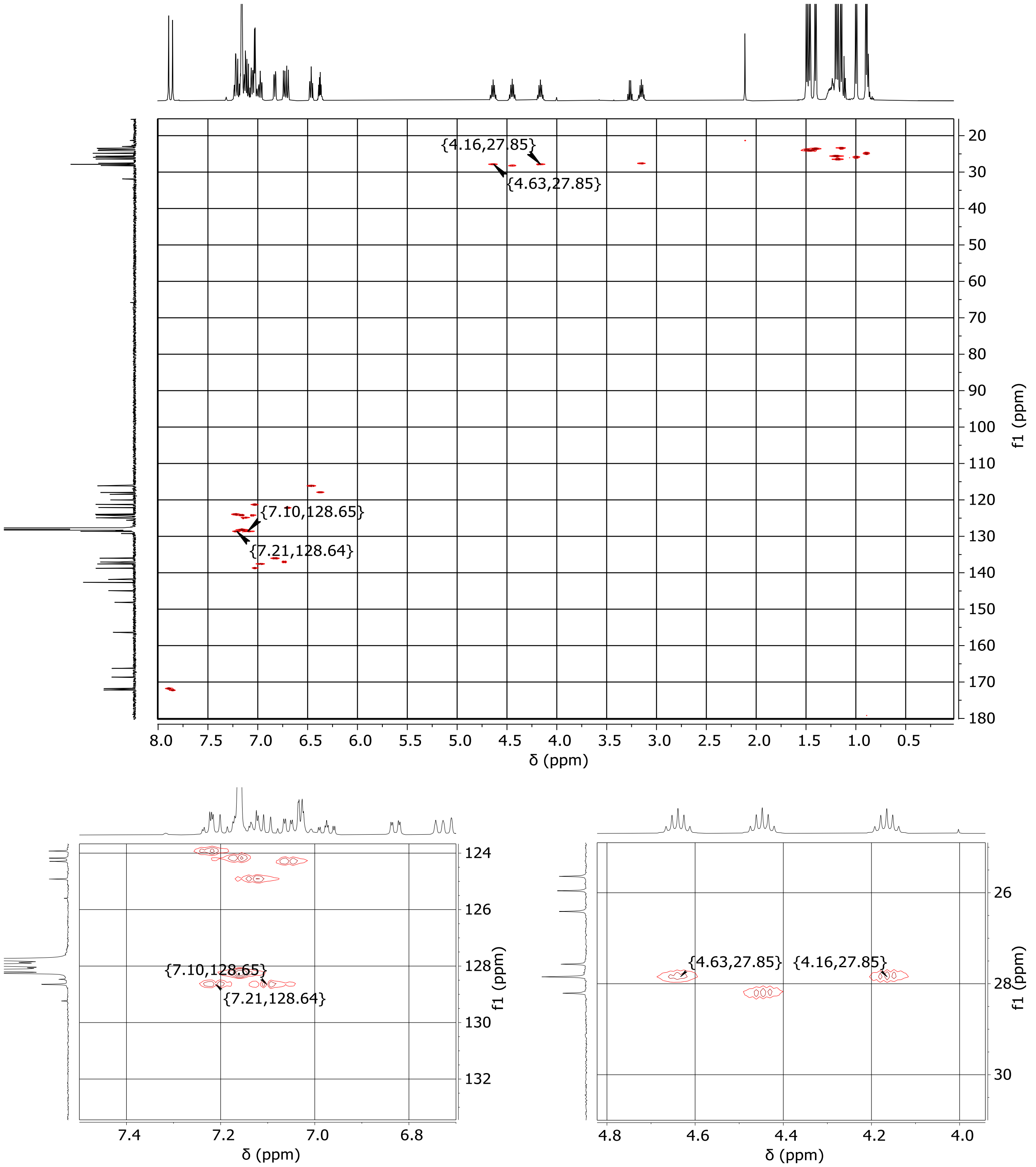

Supplementary Fig. 4. ^{1}H-^{13}C HSQC NMR spectrum of [(salNdipp)$_2$(Cl)OsN] (**1**) in C_6D_6 (500, 126 MHz). Cross-peaks from ^{13}C resonances that are overlapped in 1D NMR are highlighted (128.65, 128.64 ppm and 27.85 ppm).

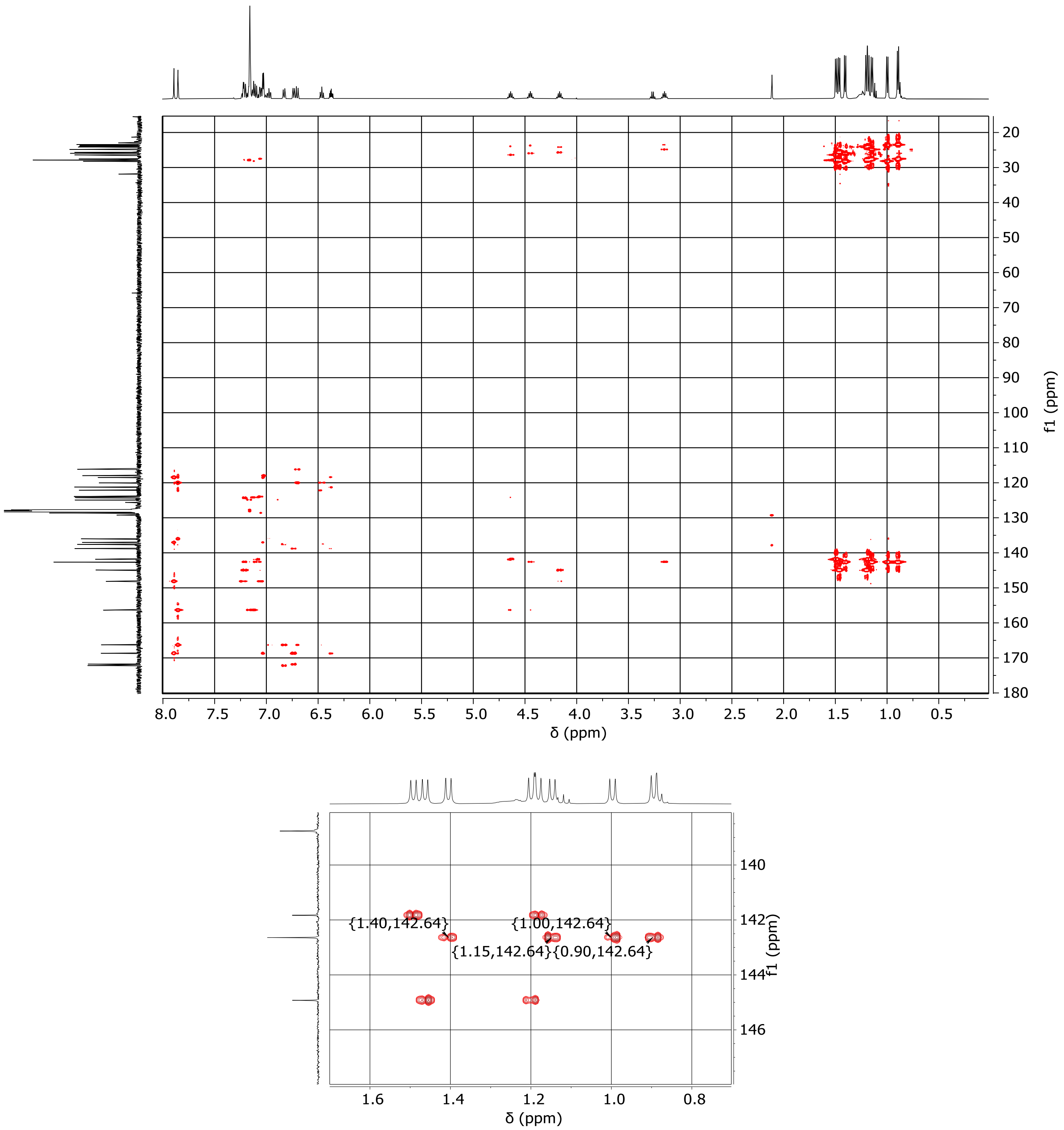

Supplementary Fig. 5. ^{1}H-^{13}C HMBC NMR spectrum of [(salNdipp)$_2$(Cl)OsN] (**1**) in C_6D_6 (500, 126 MHz). Cross-peaks from ^{13}C resonances that are overlapped in 1D NMR are highlighted (142.64 ppm).

4.2 NMR Spectral Data for [(salNdipp)$_2$(OTf)OsN] (2)

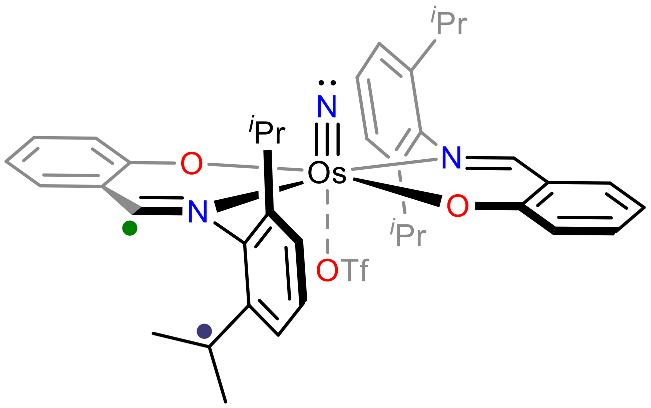

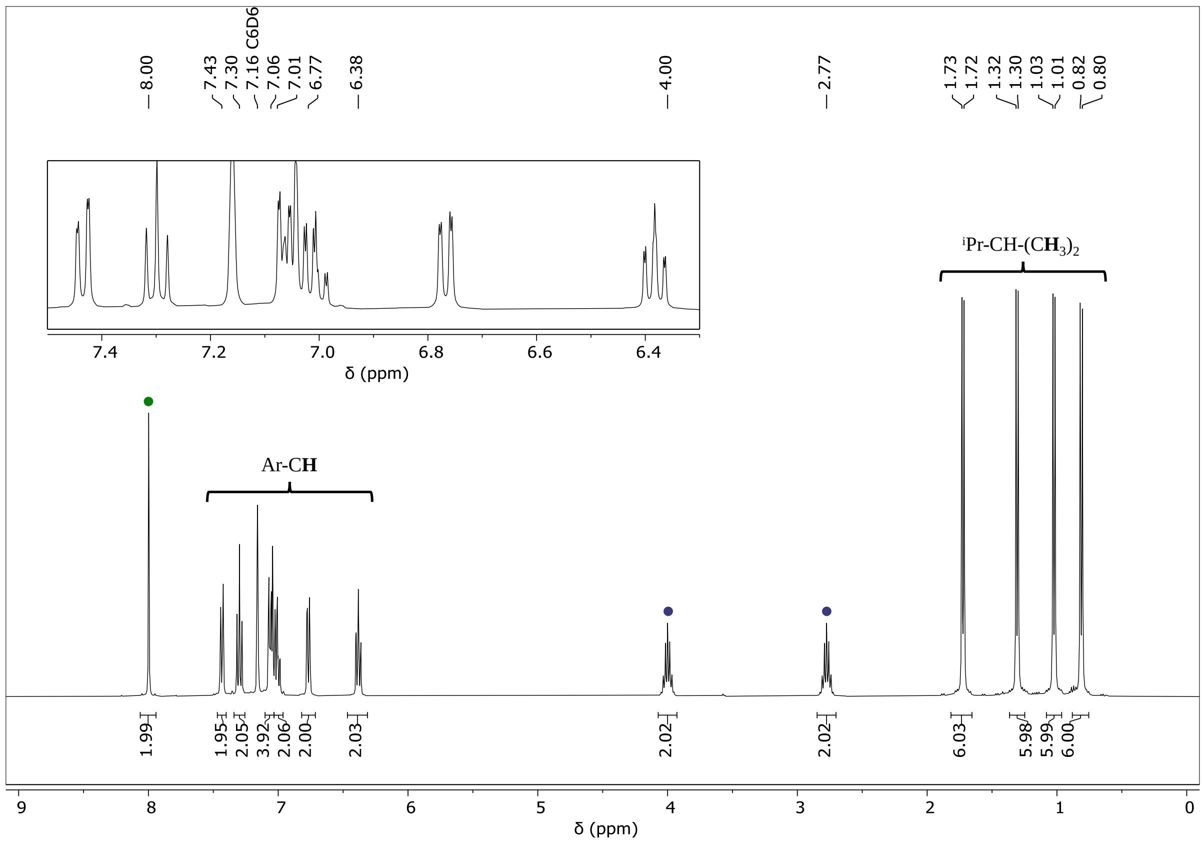

Supplementary Fig. 6. ^{1}H NMR spectrum of [(salNdipp)$_2$(OTf)OsN] (**2**) in C_6D_6 (500 MHz).

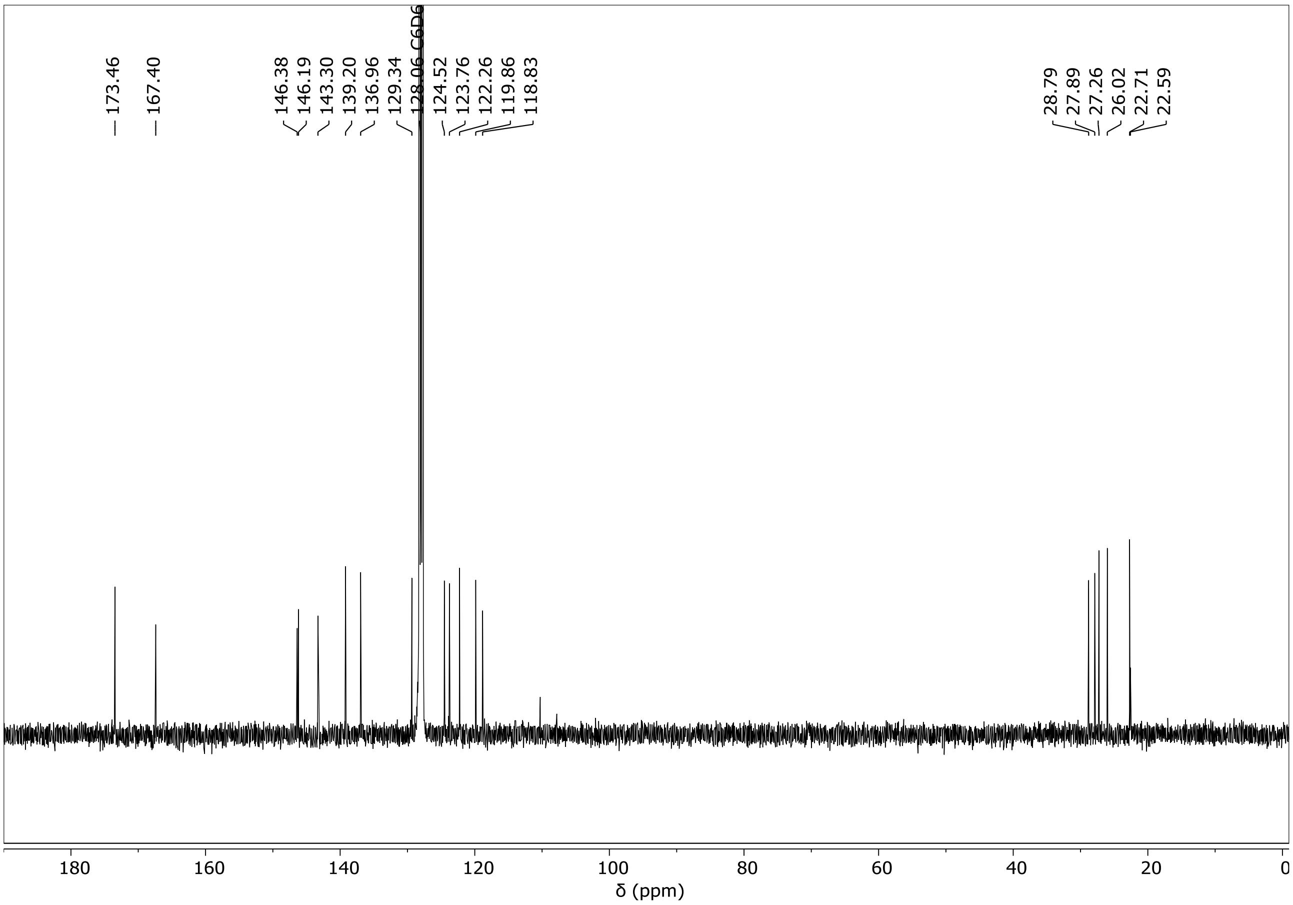

Supplementary Fig. 7. $^{13}C\{^{1}H\}$ NMR spectrum of [(salNdipp)$_2$(OTf)OsN] (**2**) in C_6D_6 (126 MHz).

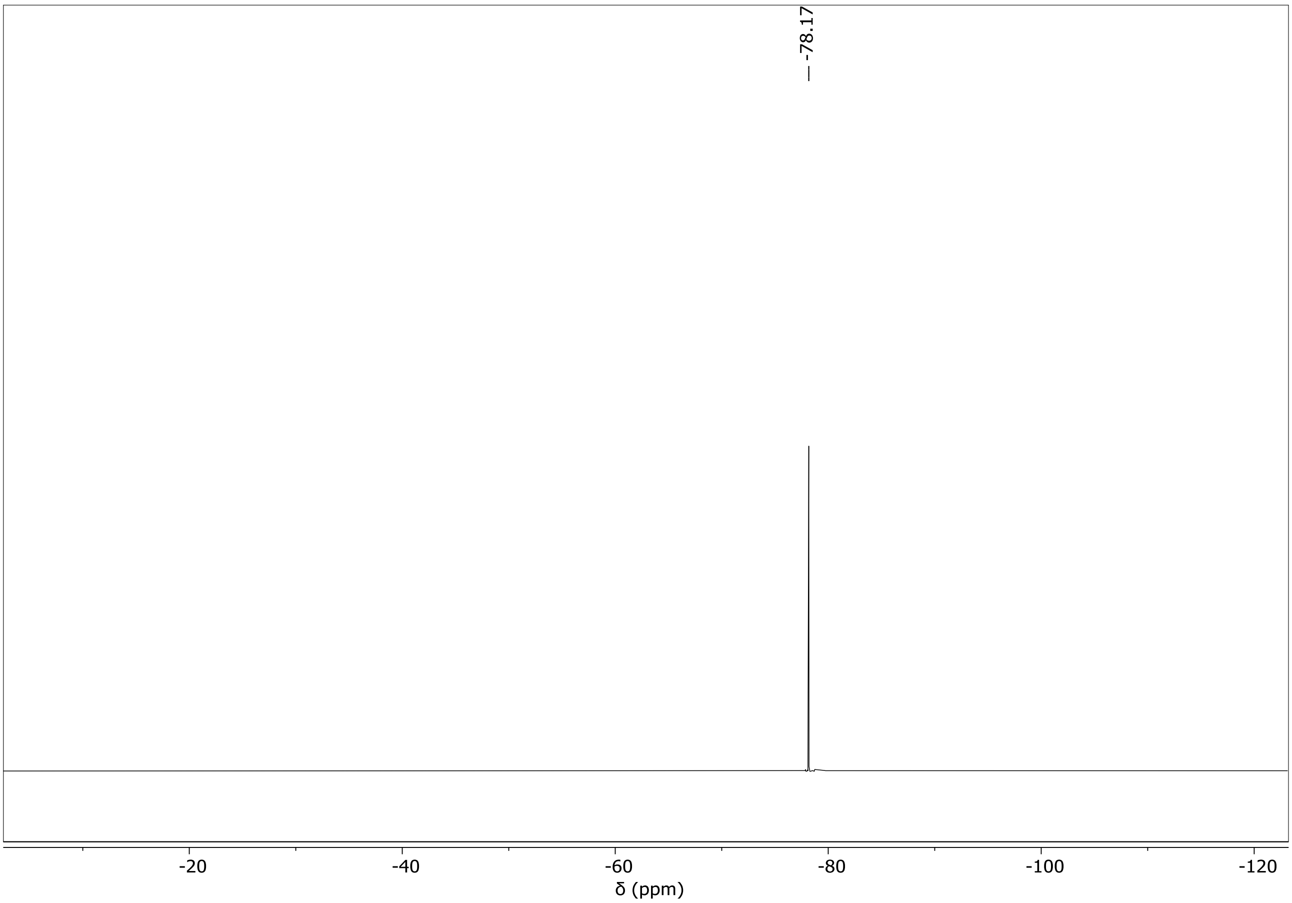

Supplementary Fig. 8. ^{19}F NMR spectrum of [(salNdipp)$_2$(OTf)OsN] (**2**) in C_6D_6 (376 MHz).

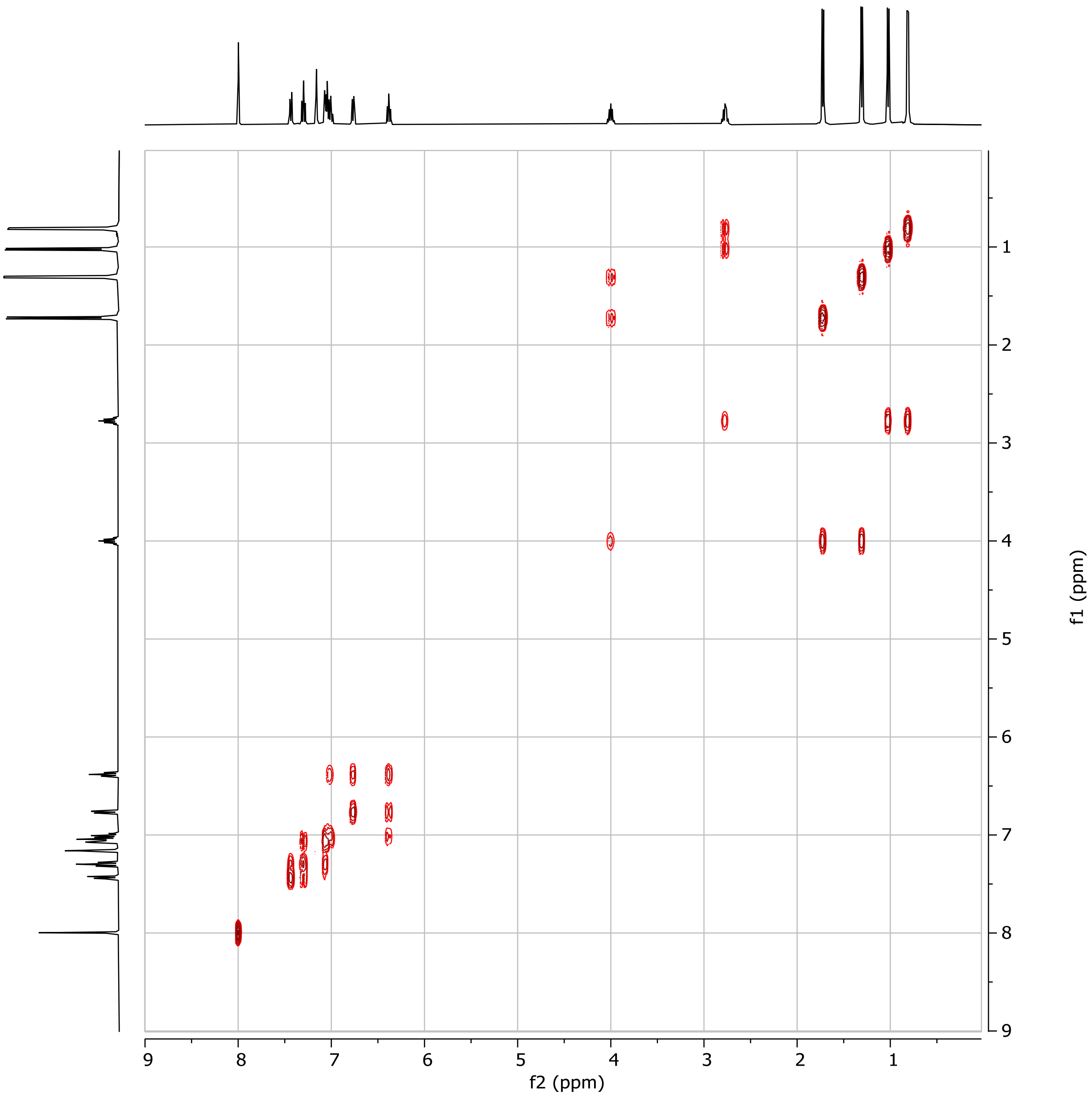

Supplementary Fig. 9. COSY NMR spectrum of [(salNdipp)$_2$(OTf)OsN] (**2**) in C_6D_6 (500 MHz).

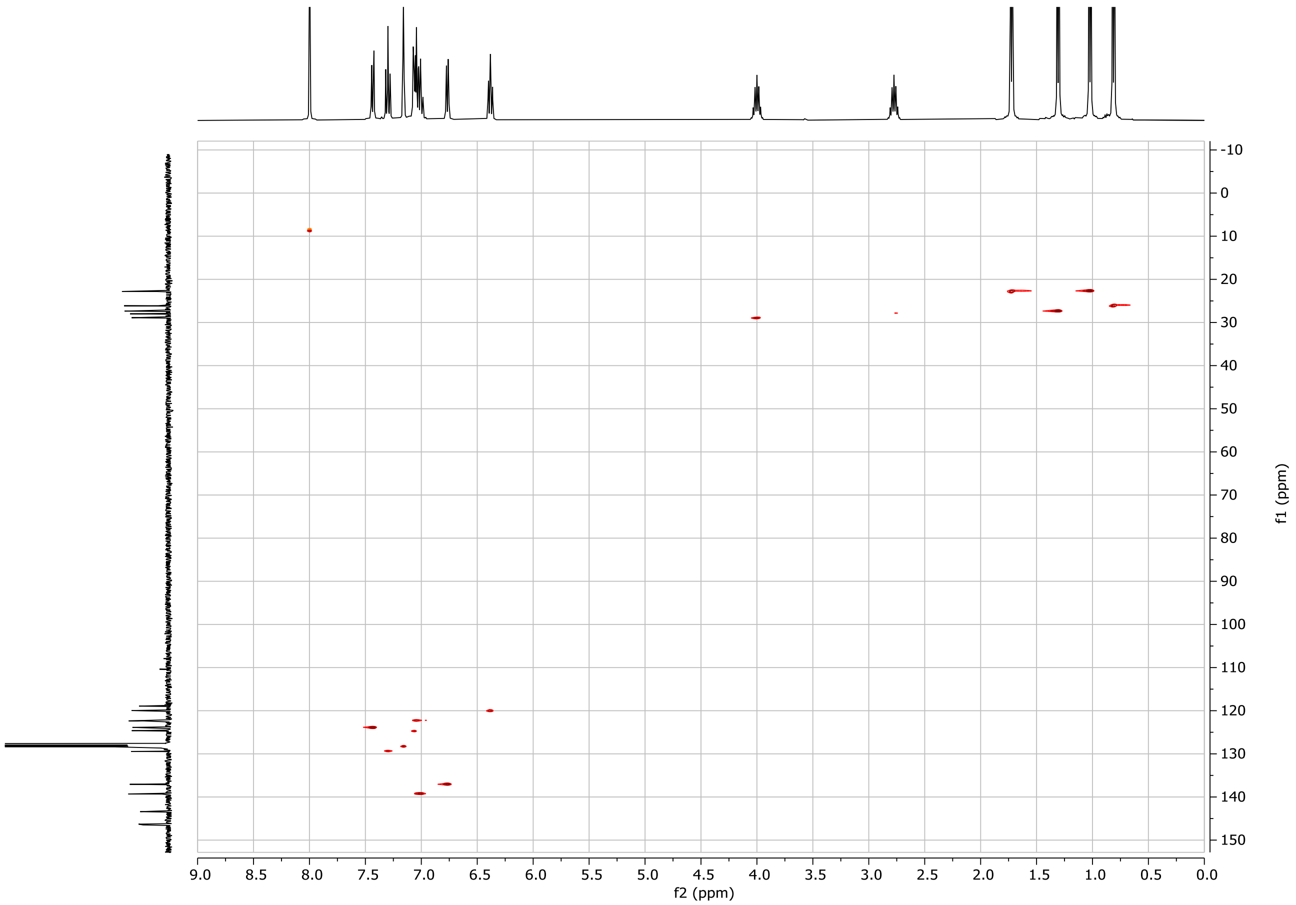

Supplementary Fig. 10. ^{1}H-^{13}C HSQC NMR spectrum of [(salNdipp)$_2$(OTf)OsN] (**2**) in C_6D_6 (500, 126 MHz).

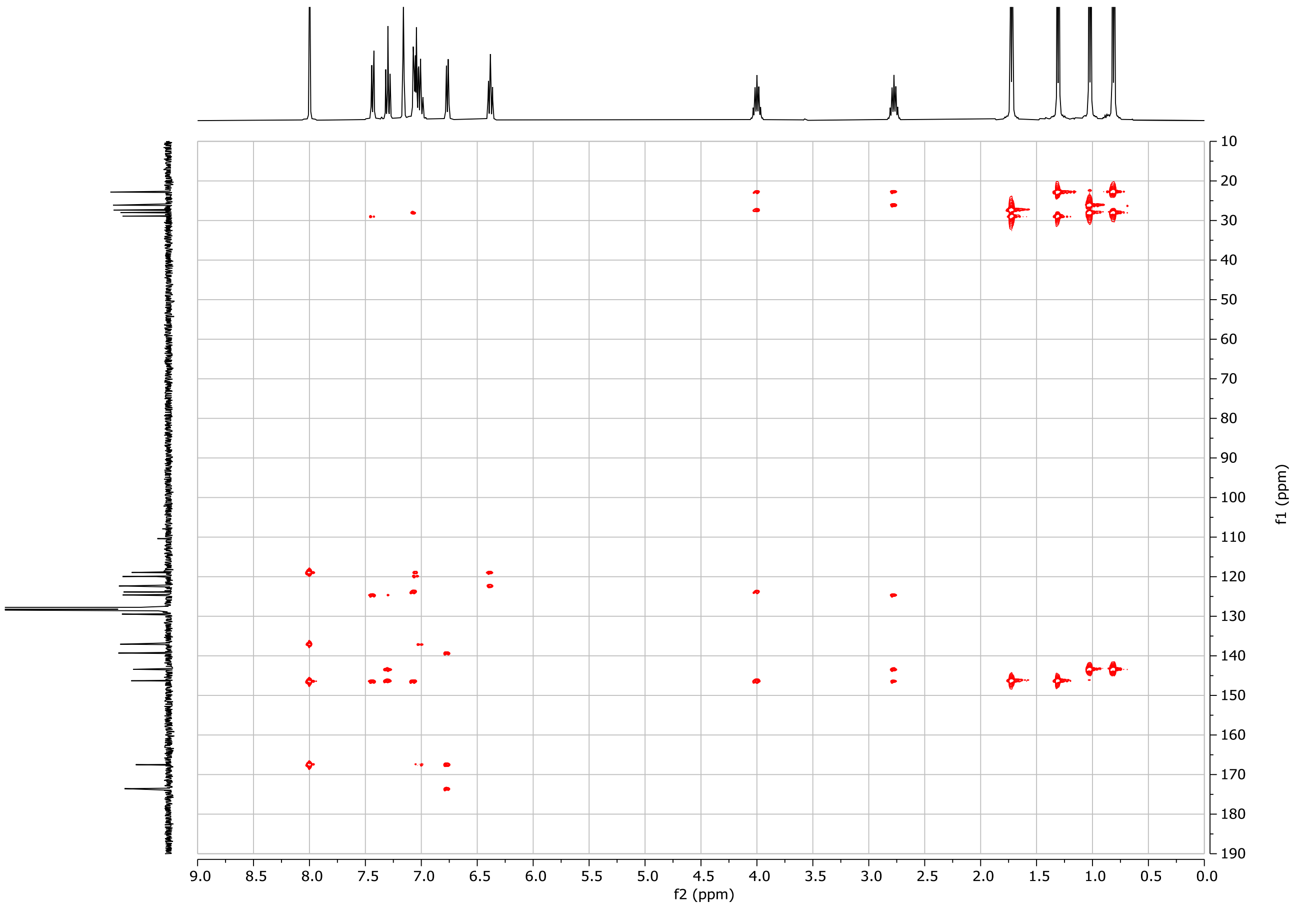

Supplementary Fig. 11. ^{1}H-^{13}C HMBC NMR spectrum of [(salNdipp)$_2$(OTf)OsN] (**2**) in C_6D_6 (500, 126 MHz).

4.3 NMR Spectral Data for [(salNdipp)$_2$(DMAP)Os(NP)] (3)

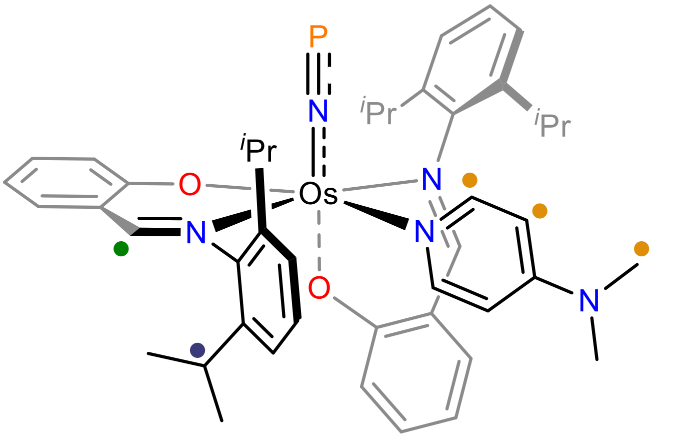

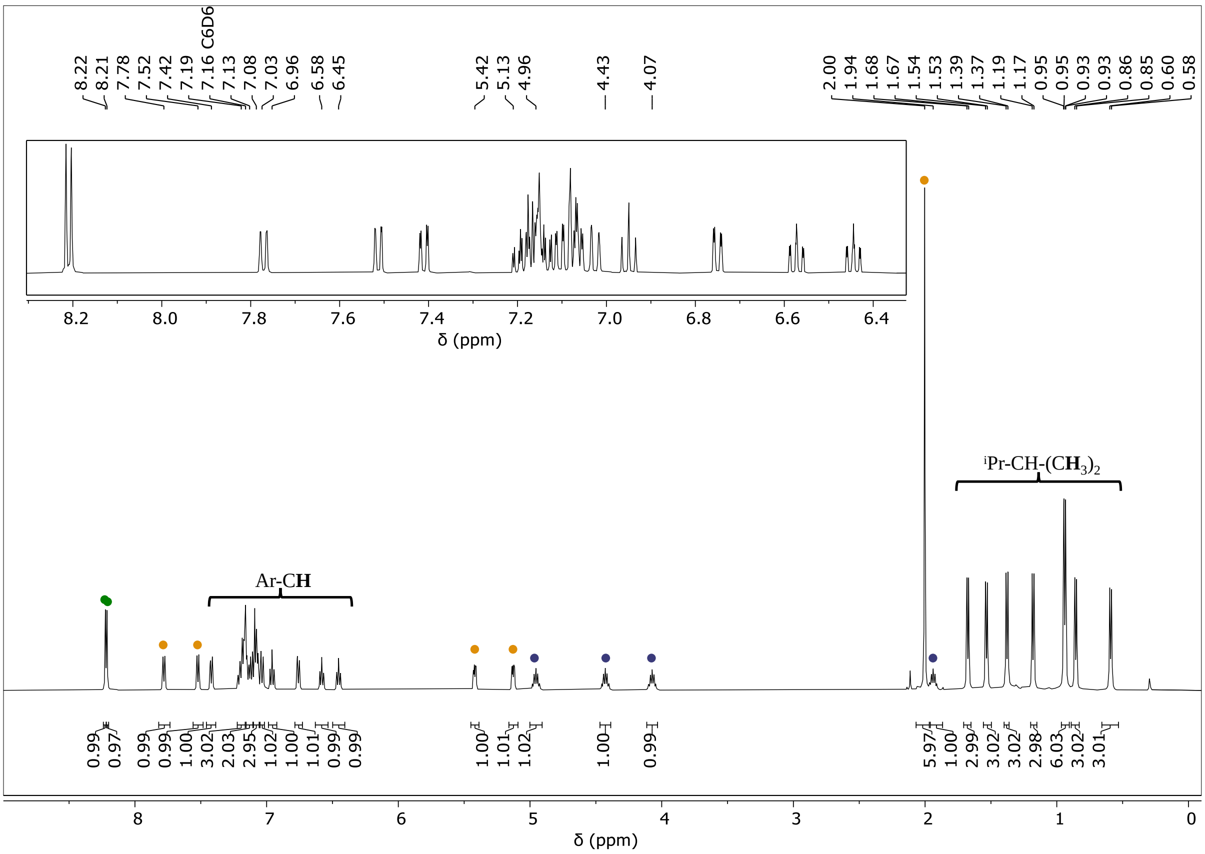

Supplementary Fig. 12. ^{1}H NMR spectrum of [(salNdipp)$_2$(DMAP)Os(NP)] (**3**) in C_6D_6 (500 MHz). The resonances at 7.13, 7.03, and 2.11 ppm (toluene) arise from traces of solvent.

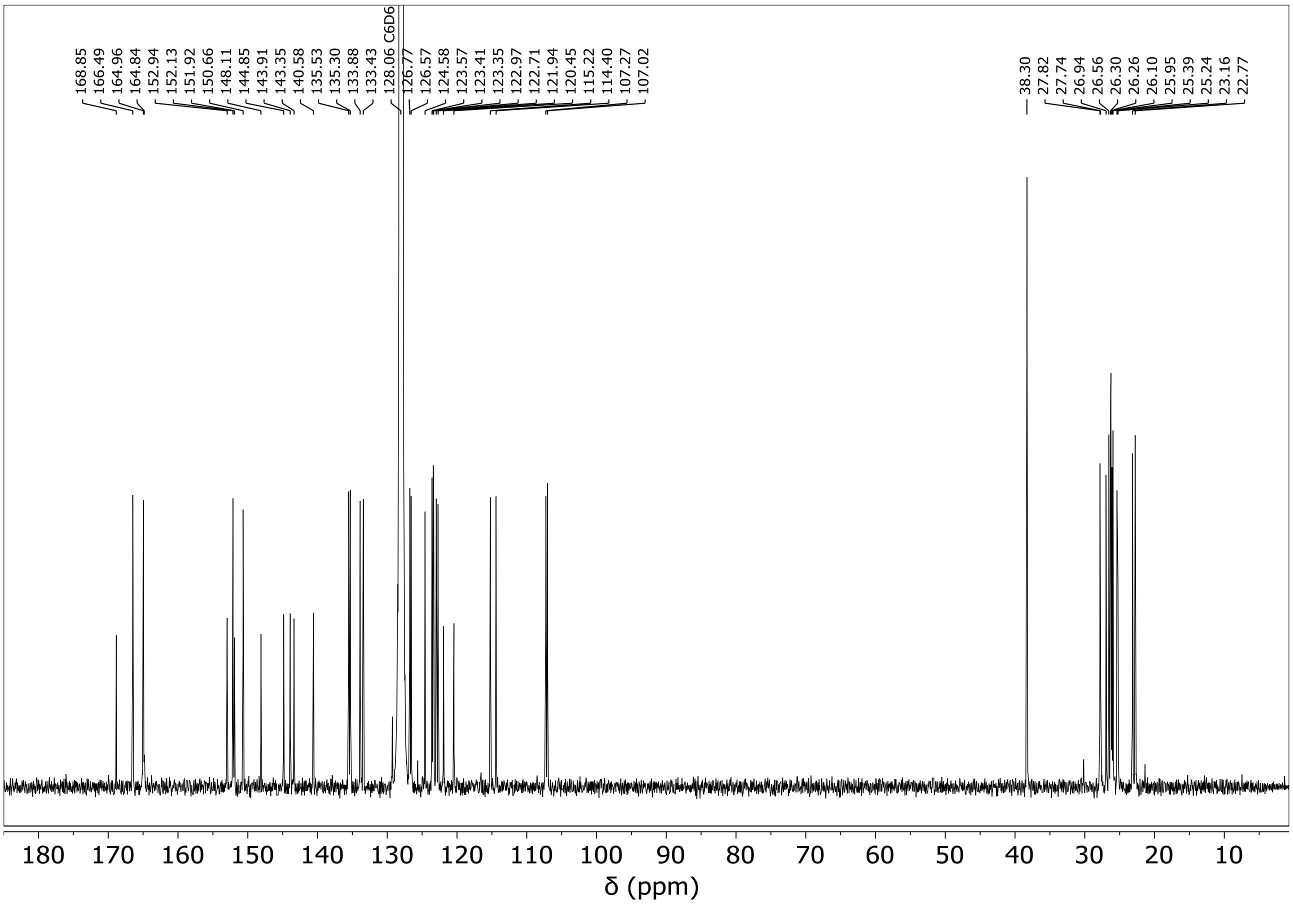

Supplementary Fig. 13. $^{13}C\{^{1}H\}$ NMR spectrum of [(salNdipp)$_2$(DMAP)Os(NP)] (**3**) in C_6D_6 (126 MHz).

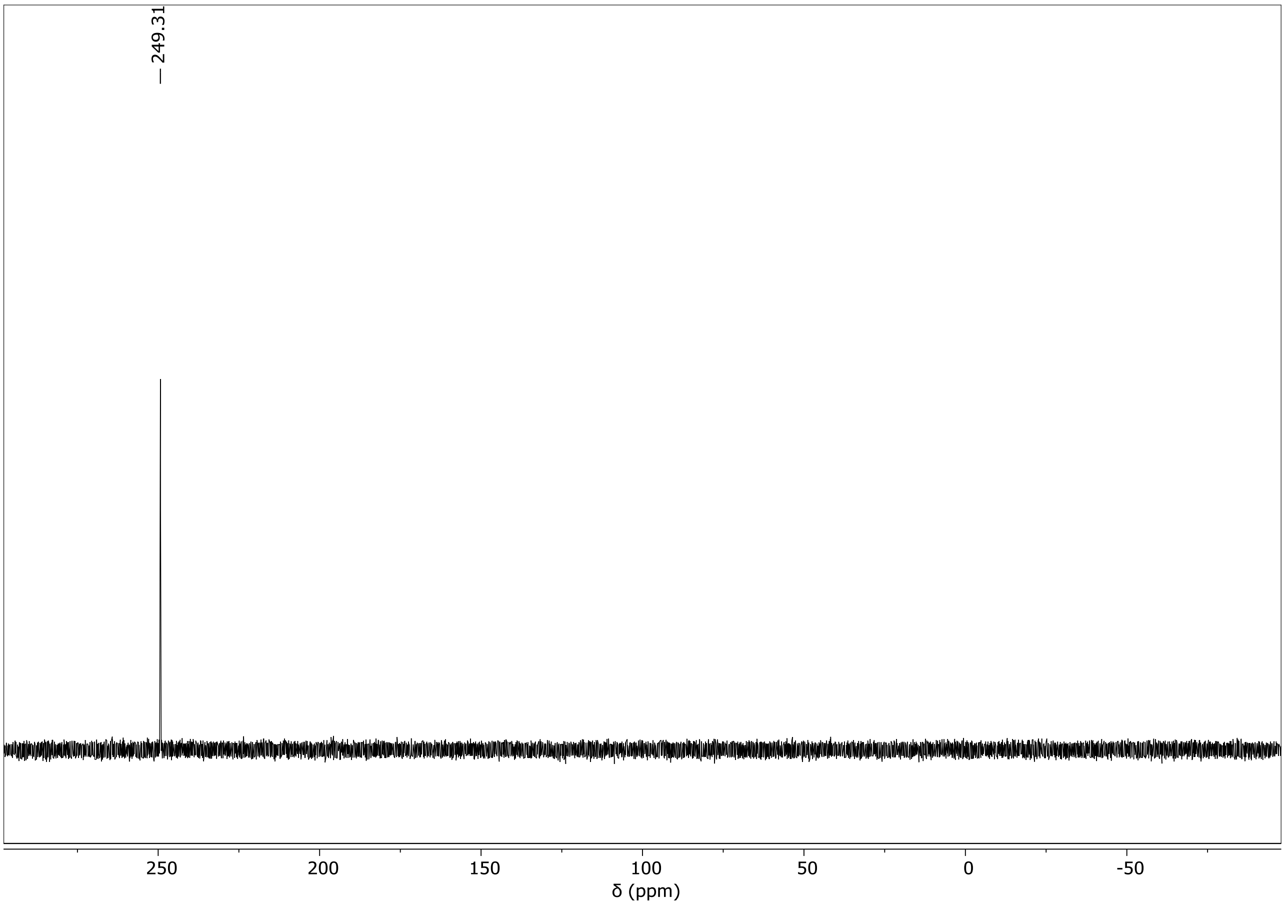

Supplementary Fig. 14. $^{31}P\{^{1}H\}$ NMR spectrum of [(salNdipp)$_2$(DMAP)Os(NP)] (**3**) in C_6D_6 (162 MHz).

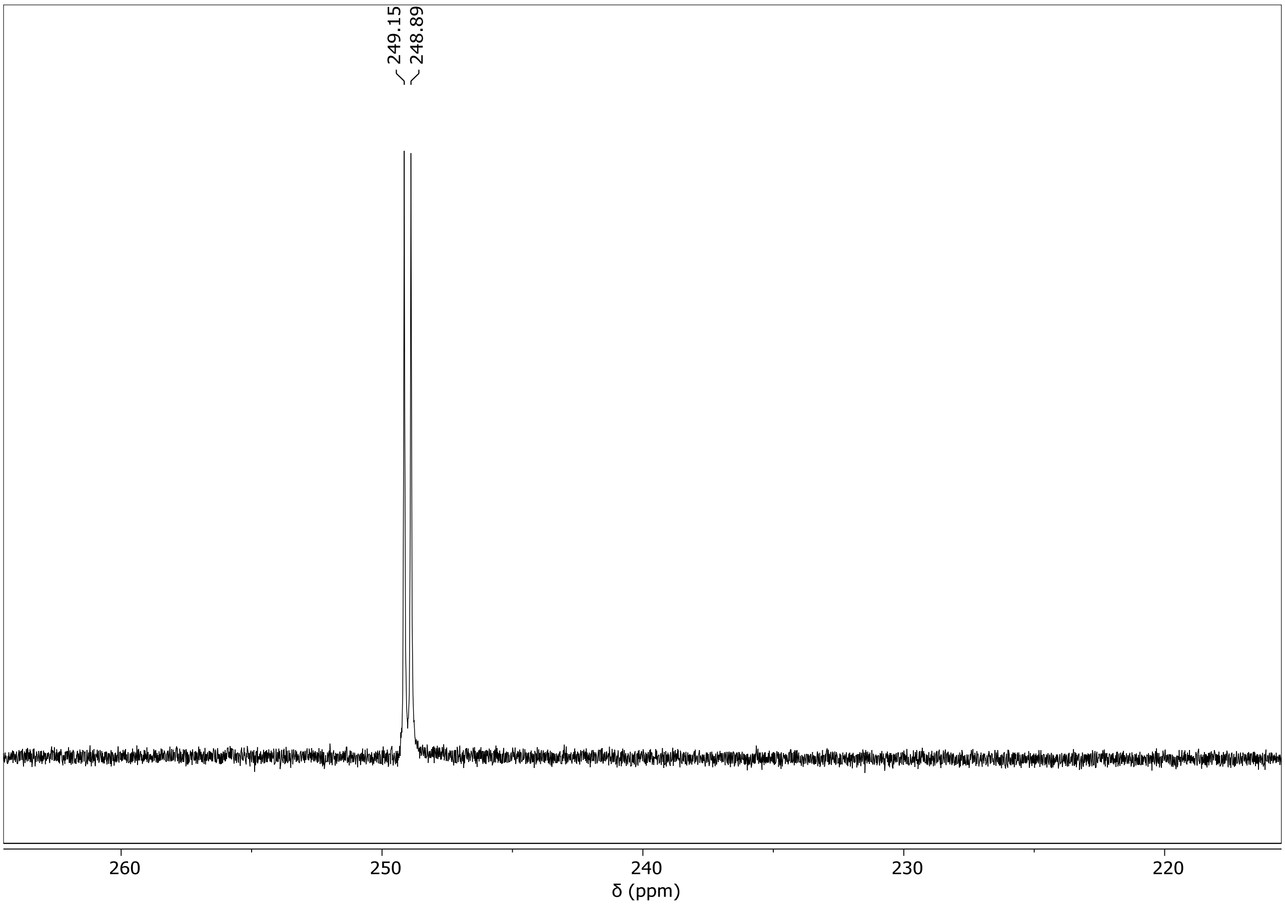

Supplementary Fig. 15. $^{31}P\{^{1}H\}$ NMR spectrum of [(salNdipp)$_2$(DMAP)Os(^{15}NP)] (**3-**^{15}N) in C_6D_6 (243 MHz).

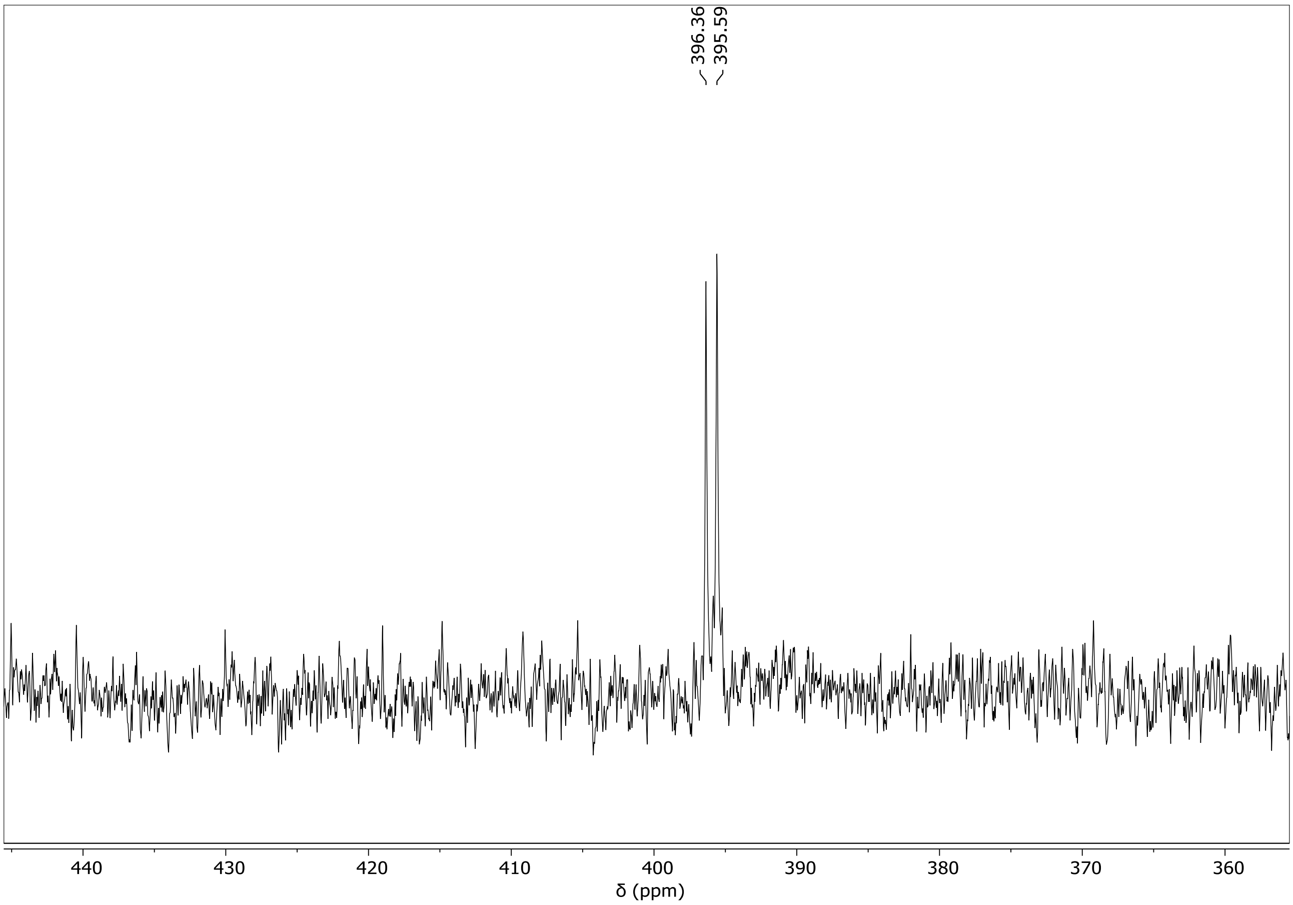

Supplementary Fig. 16. $^{15}N\{^{1}H\}$ NMR spectrum of [(salNdipp)$_2$(DMAP)Os(^{15}NP)] (**3-**^{15}N) in C_6D_6 (81 MHz).

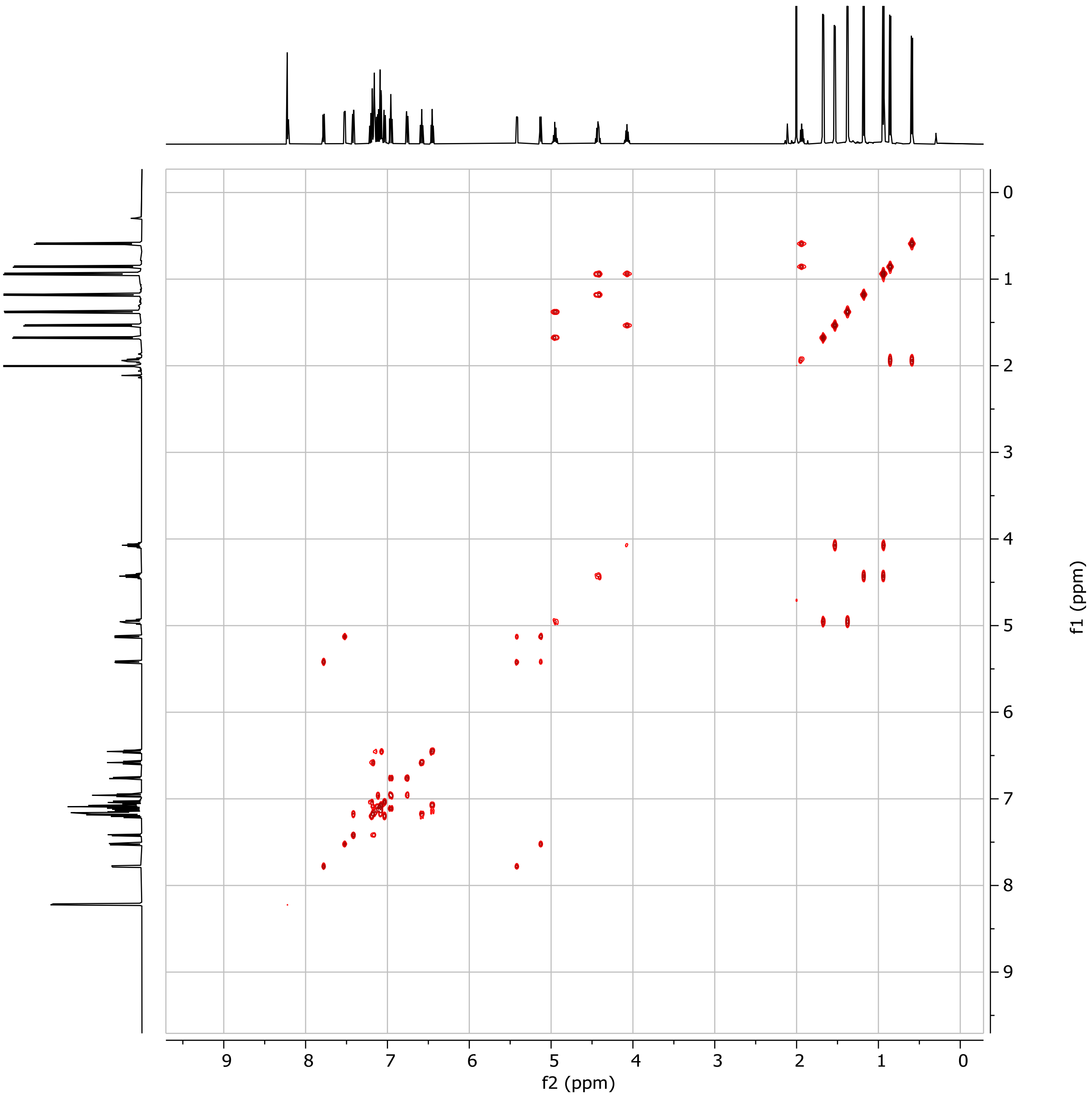

Supplementary Fig. 17. COSY NMR spectrum of [(salNdipp)$_2$(DMAP)Os(NP)] (**3**) in C_6D_6 (500 MHz).

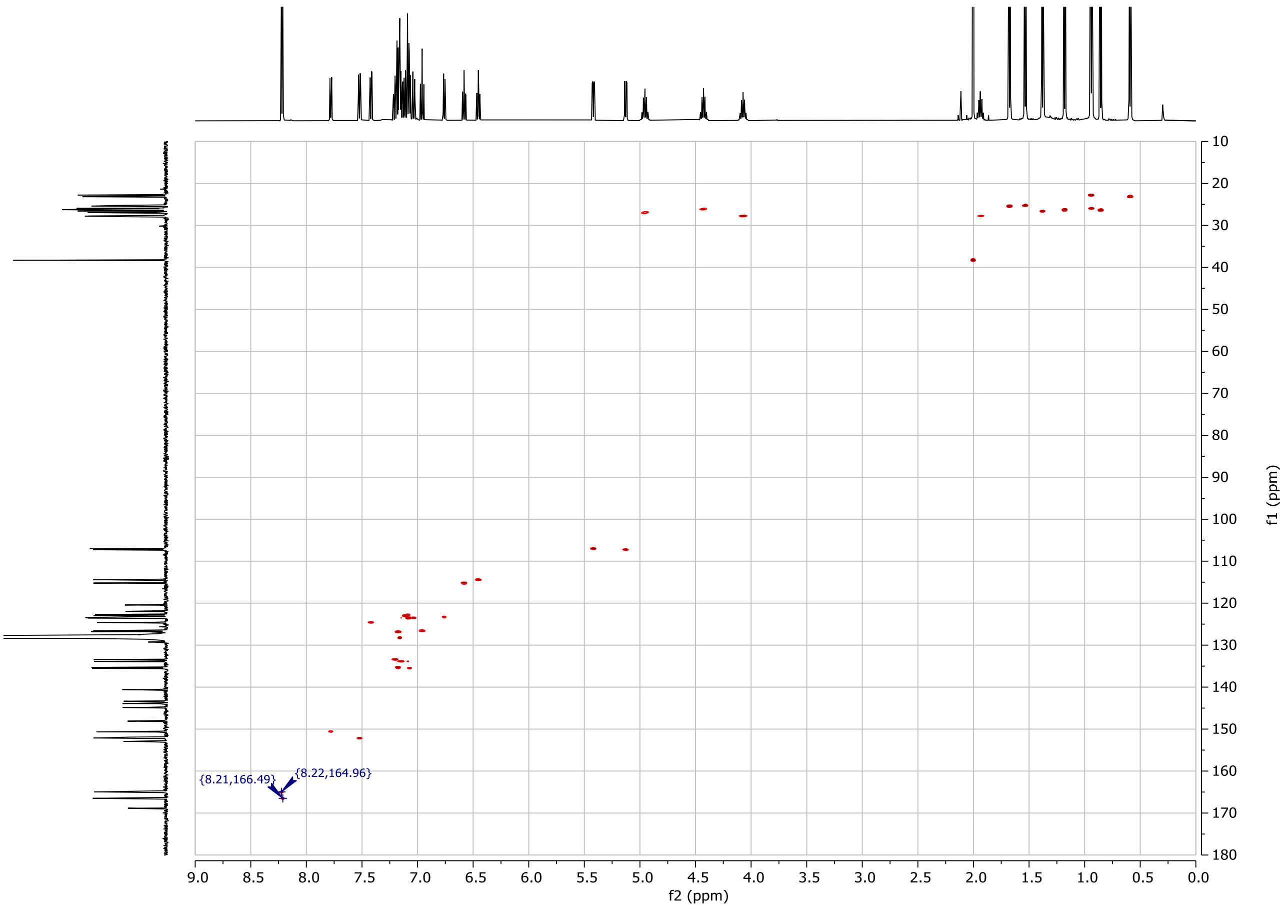

Supplementary Fig. 18. Phase-edited ^{1}H-^{13}C HSQC NMR spectrum of [(salNdipp)$_2$(DMAP)Os(NP)] (**3**) in C_6D_6 (500, 126 MHz). Cross-peaks from imine CH groups are highlighted.

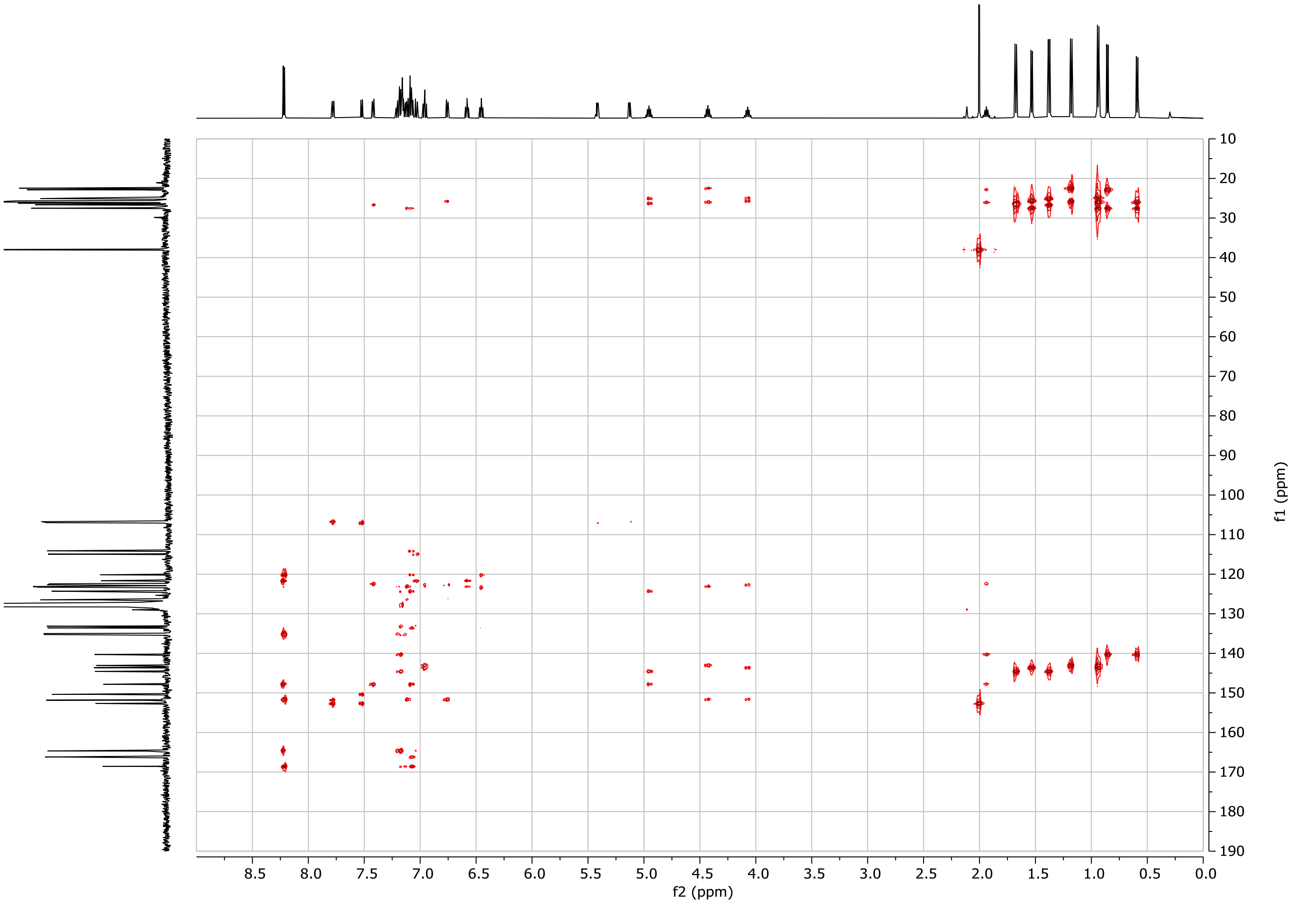

Supplementary Fig. 19. ^{1}H-^{13}C HMBC NMR spectrum of [(salNdipp)$_2$(DMAP)Os(NP)] (**3**) in C_6D_6 (500, 126 MHz).

4.4 NMR Spectral Data for [(salNdipp)$_2$(DMAP)Os(NPS$_2$)] (4)

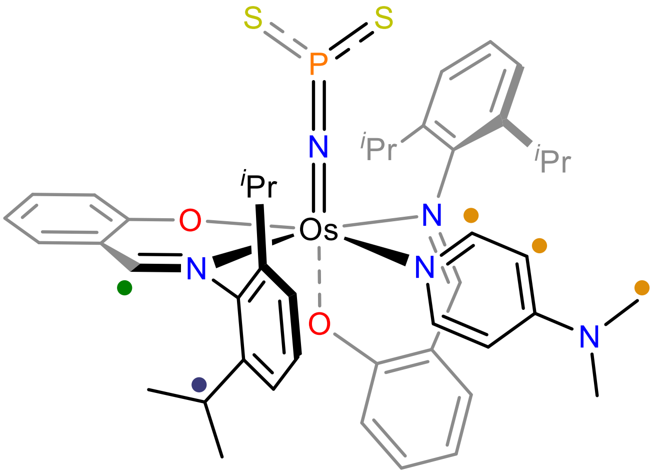

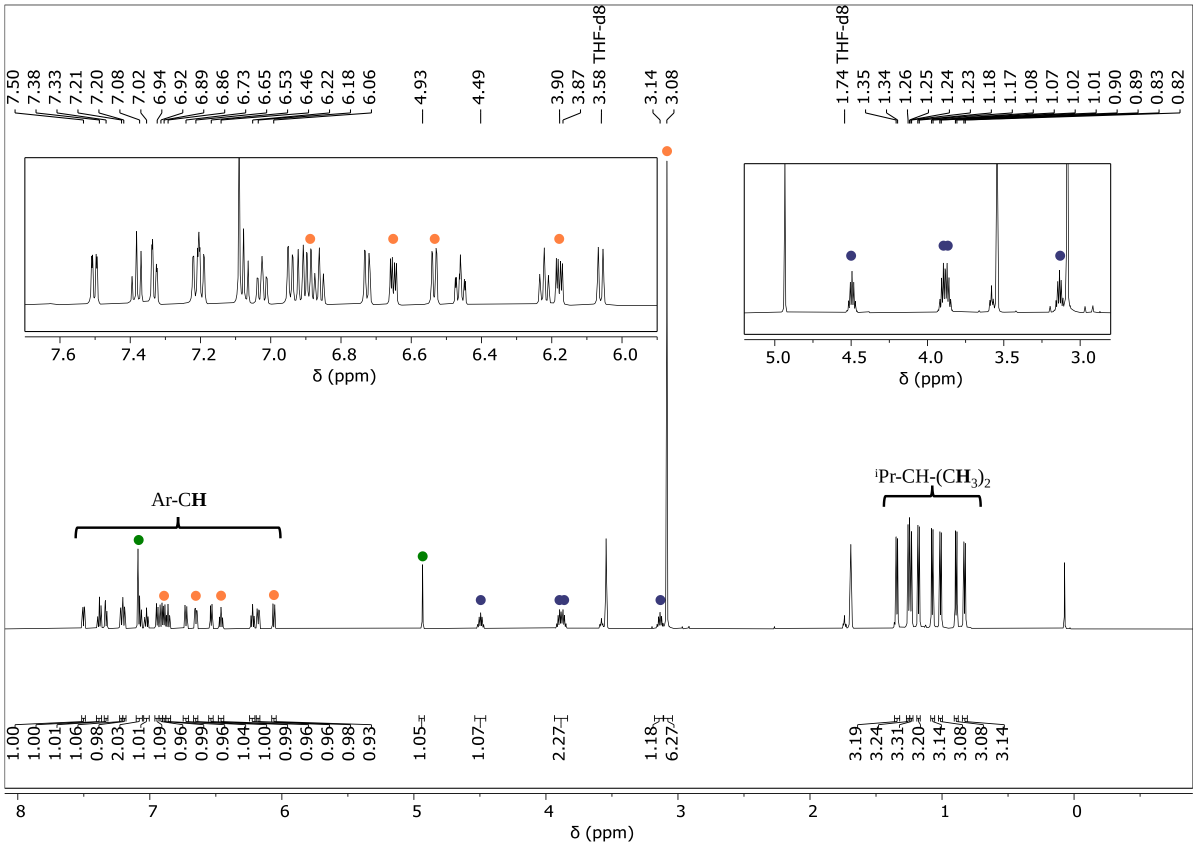

Supplementary Fig. 20. ^{1}H NMR spectrum of [(salNdipp)$_2$(DMAP)Os(NPS$_2$)] (**4**) in THF-d$_8$ (600 MHz). The resonances at 7.19, 7.10, and 2.31 ppm (toluene) arise from traces of solvent.

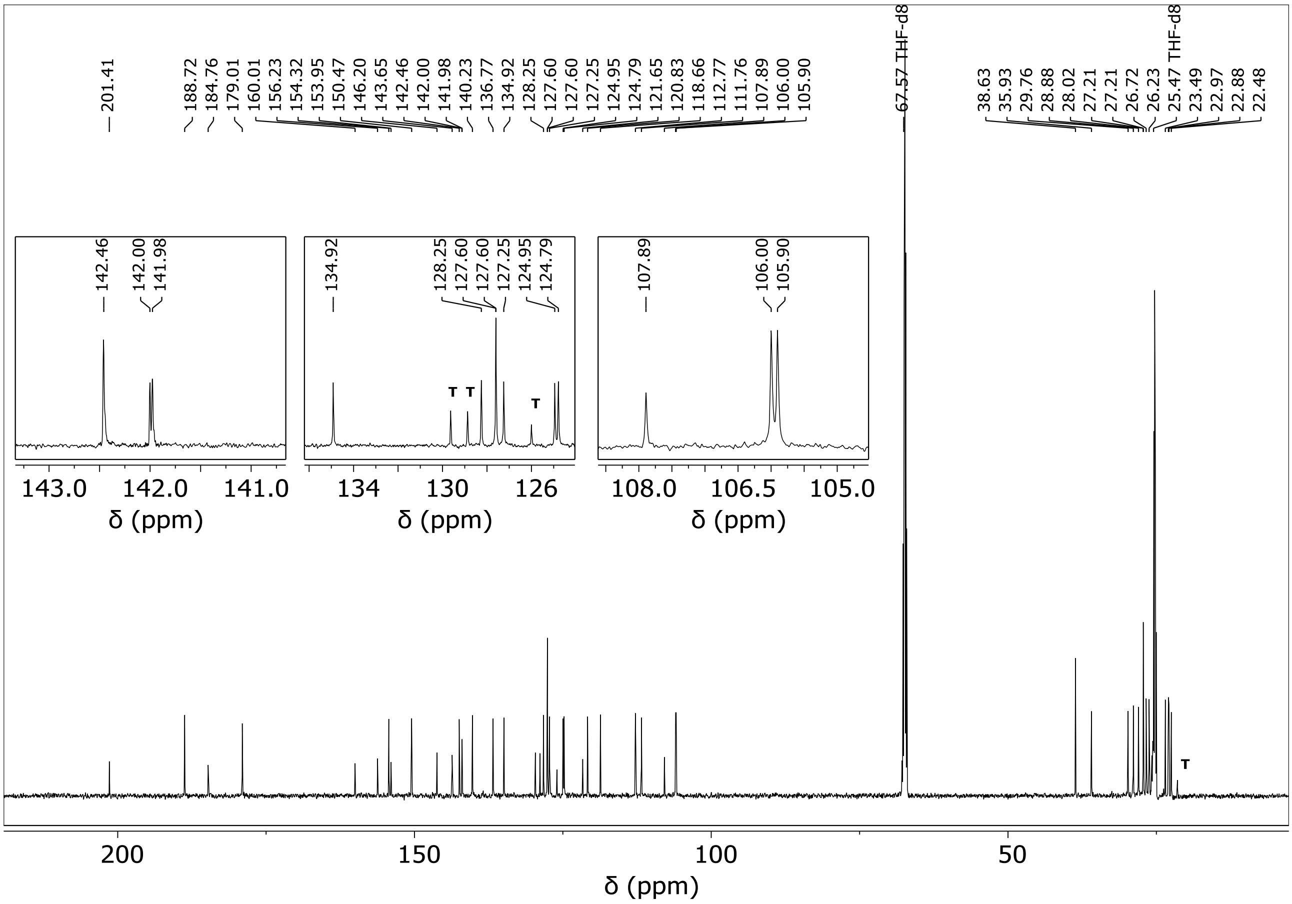

Supplementary Fig. 21. $^{13}C\{^{1}H\}$ NMR spectrum of [(salNdipp)$_2$(DMAP)Os(NPS$_2$)] (**4**) in THF-d$_8$ (151 MHz). The resonances at 129.63, 128.87, 126.00, and 21.45 ppm (toluene, **T**) arise from traces of solvent.

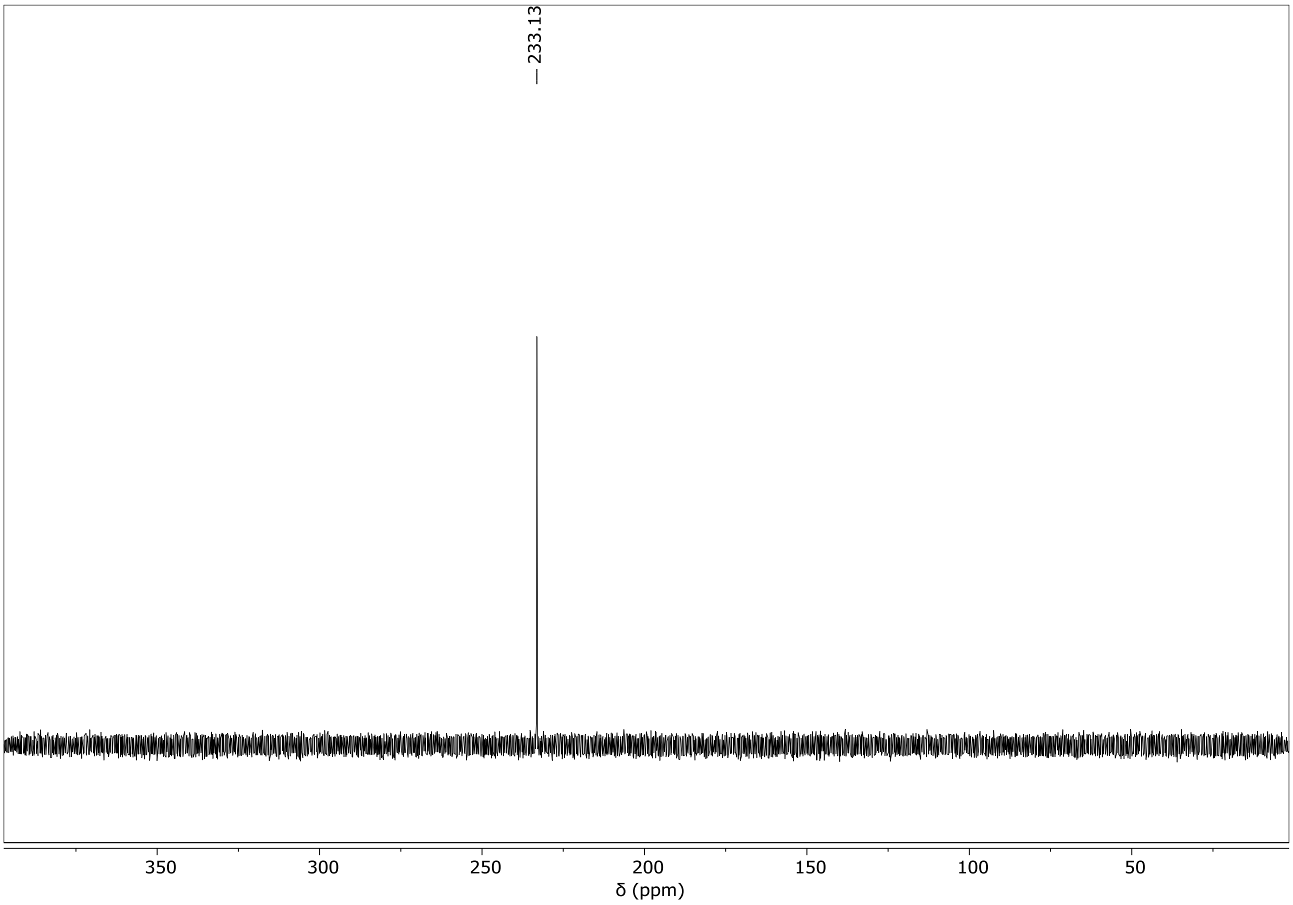

Supplementary Fig. 22. $^{31}P\{^{1}H\}$ NMR spectrum of [(salNdipp)$_2$(DMAP)Os(NPS$_2$)] (**4**) in THF-d$_8$ (243 MHz).

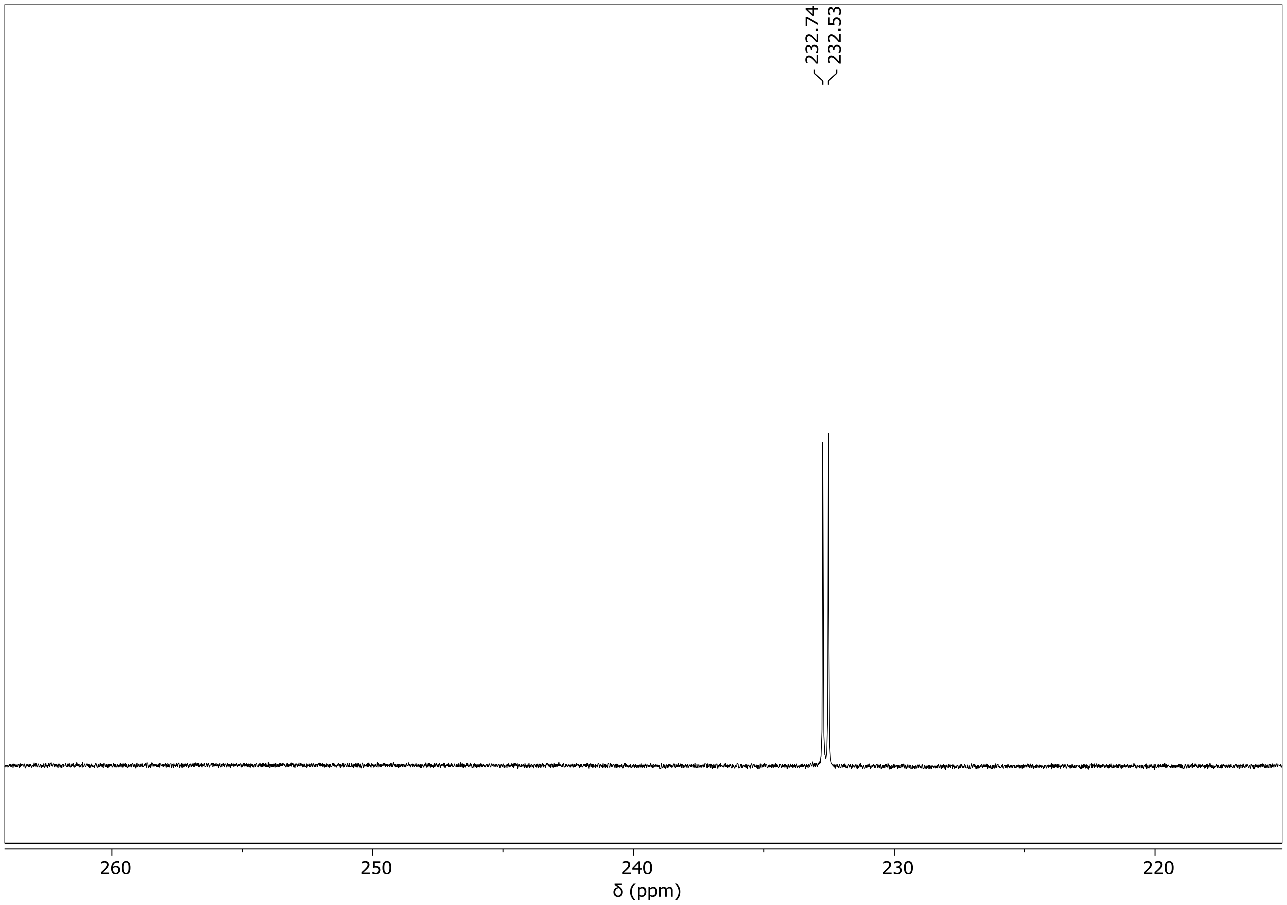

Supplementary Fig. 23. $^{31}P\{^{1}H\}$ NMR spectrum of [(salNdipp)$_2$(DMAP)Os($^{15}NPS_2$)] (**4-**^{15}N) in THF-d_8 (243 MHz).

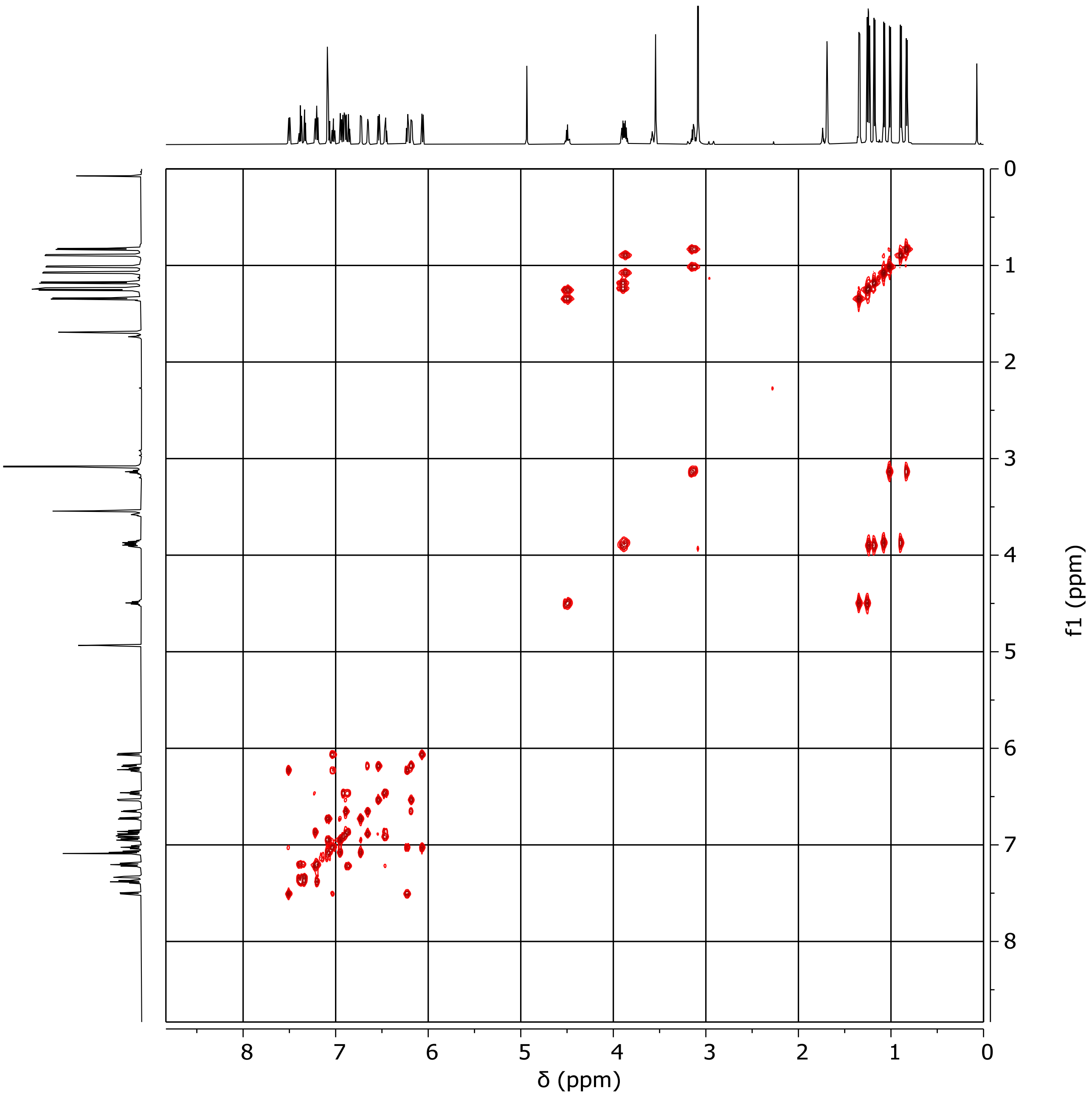

Supplementary Fig. 24. COSY NMR spectrum of [(salNdipp)$_2$(DMAP)Os(NPS$_2$)] (**4**) in THF-d$_8$ (600 MHz).

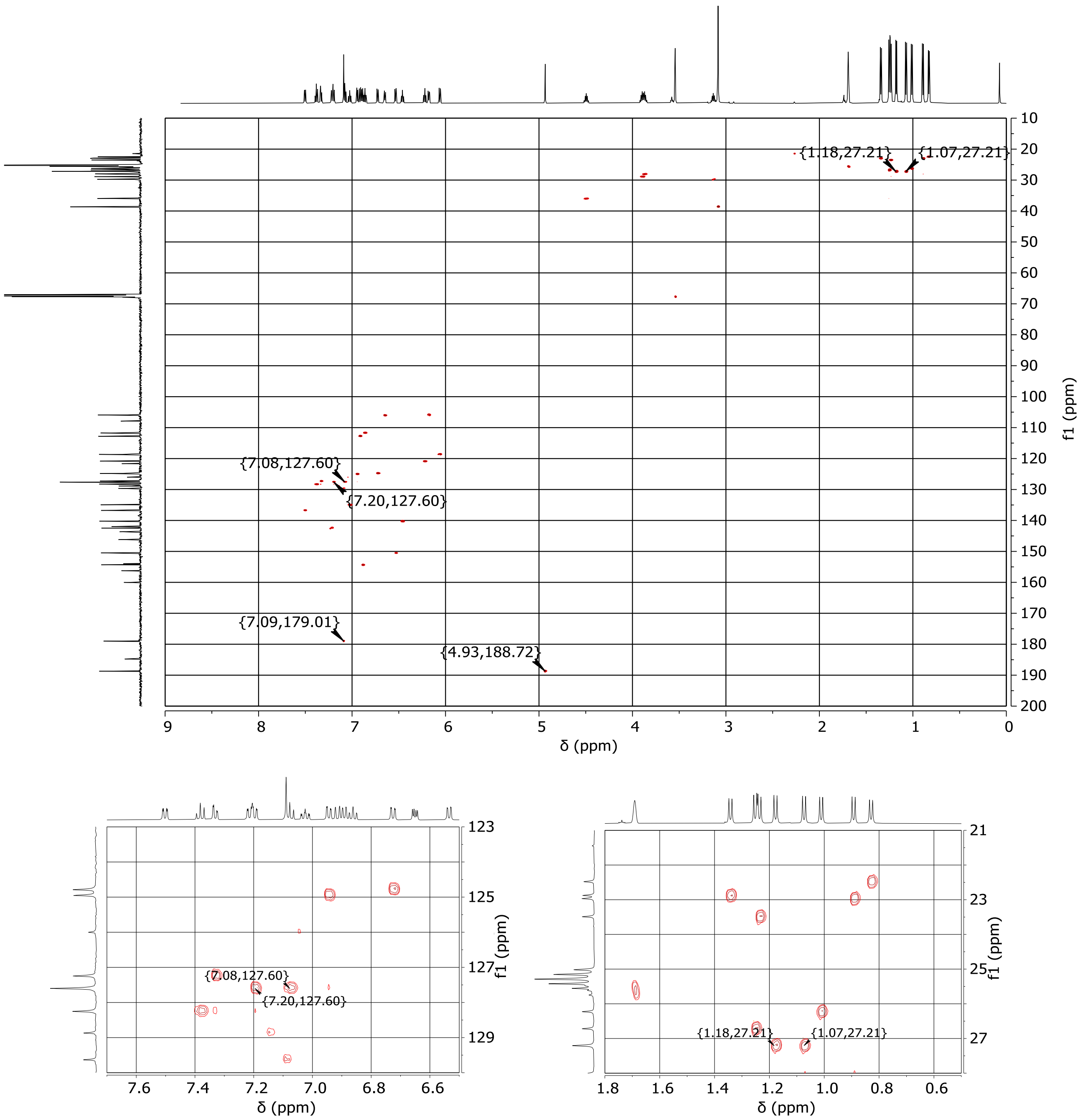

Supplementary Fig. 25. Phase-edited ^{1}H-^{13}C HSQC NMR spectrum of [(salNdipp)$_2$(DMAP)Os(NPS$_2$)] (**4**) in THF-d$_8$ (600, 151 MHz). Cross-peaks from imine CH groups are highlighted. Cross-peaks from ^{13}C resonances that are overlapped in 1D NMR are also highlighted (127.60 ppm and 27.21 ppm).

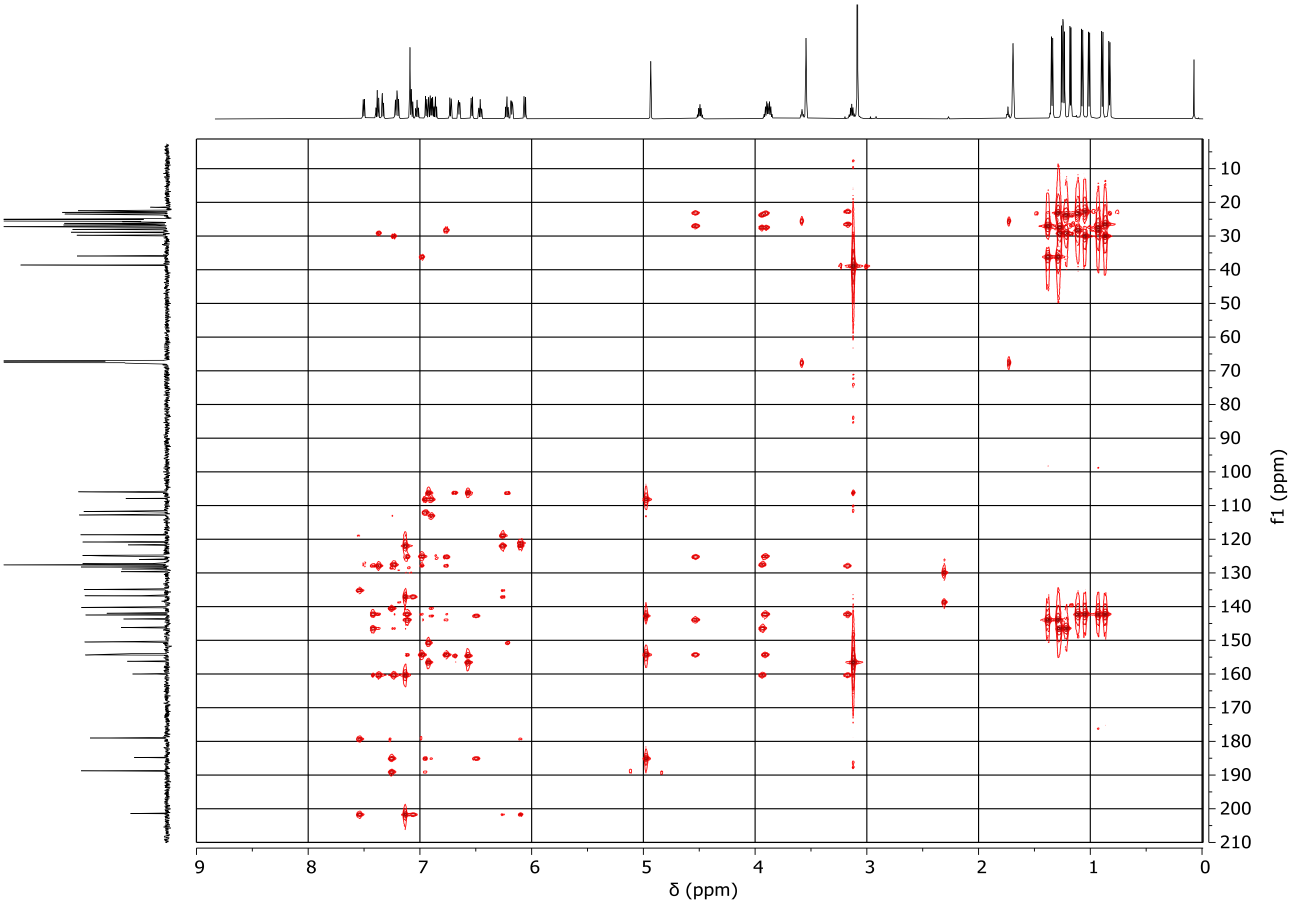

Supplementary Fig. 26. ^{1}H-^{13}C HMBC NMR spectrum of [(salNdipp)$_2$(DMAP)Os(NPS$_2$)] (**4**) in THF-d$_8$ (600, 151 MHz).

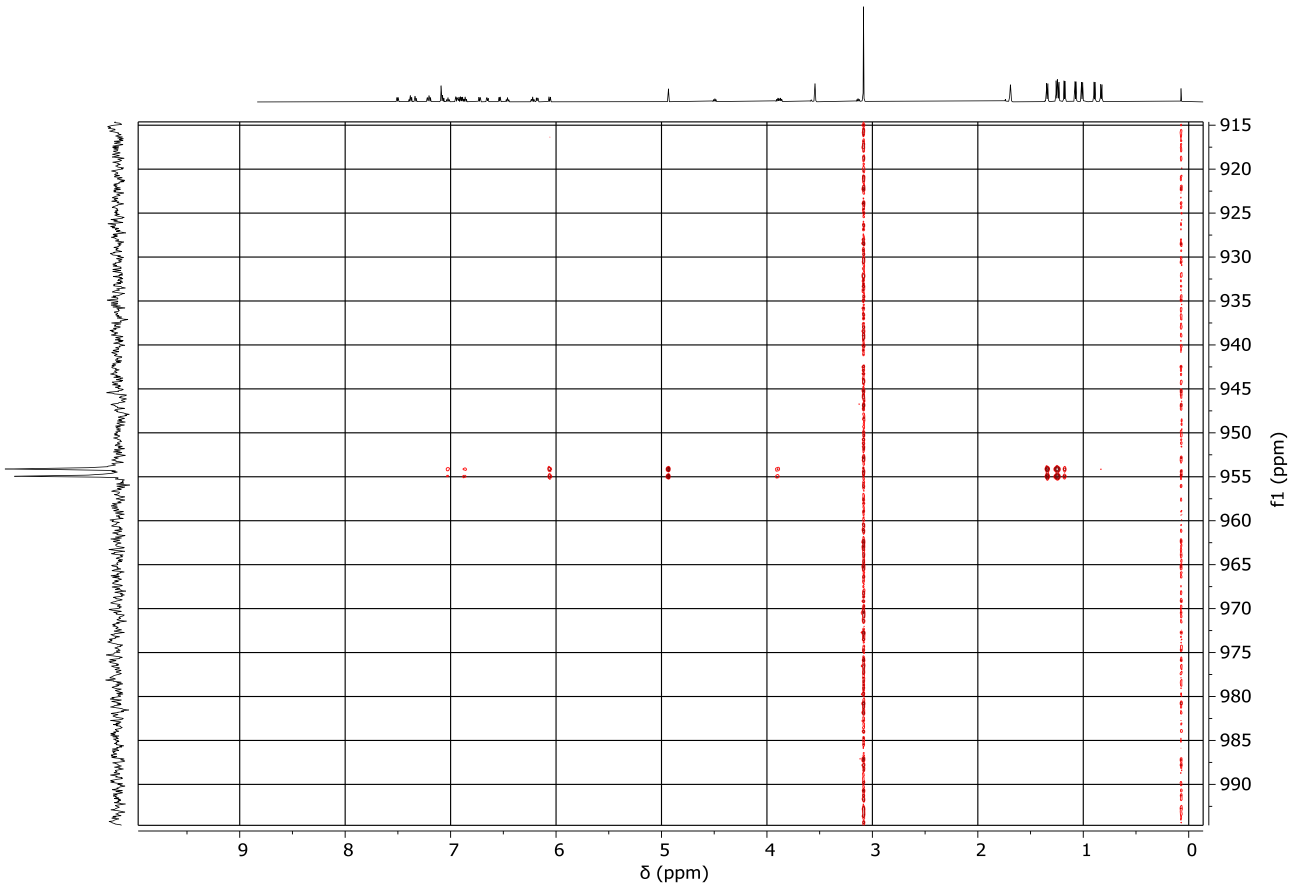

Supplementary Fig. 27. ^{1}H-^{15}N HMBC NMR spectrum of [(salNdipp)$_2$(DMAP)Os($^{15}NPS_2$)] (**4**-^{15}N) in THF-d_8 (600, 61 MHz)

4.5 NMR Spectral Data for [(salNdipp)$_2$(DMAP)Os(NPCl)] (5)

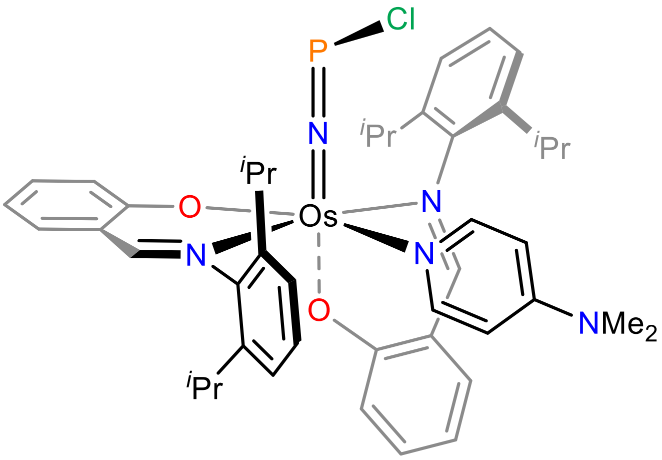

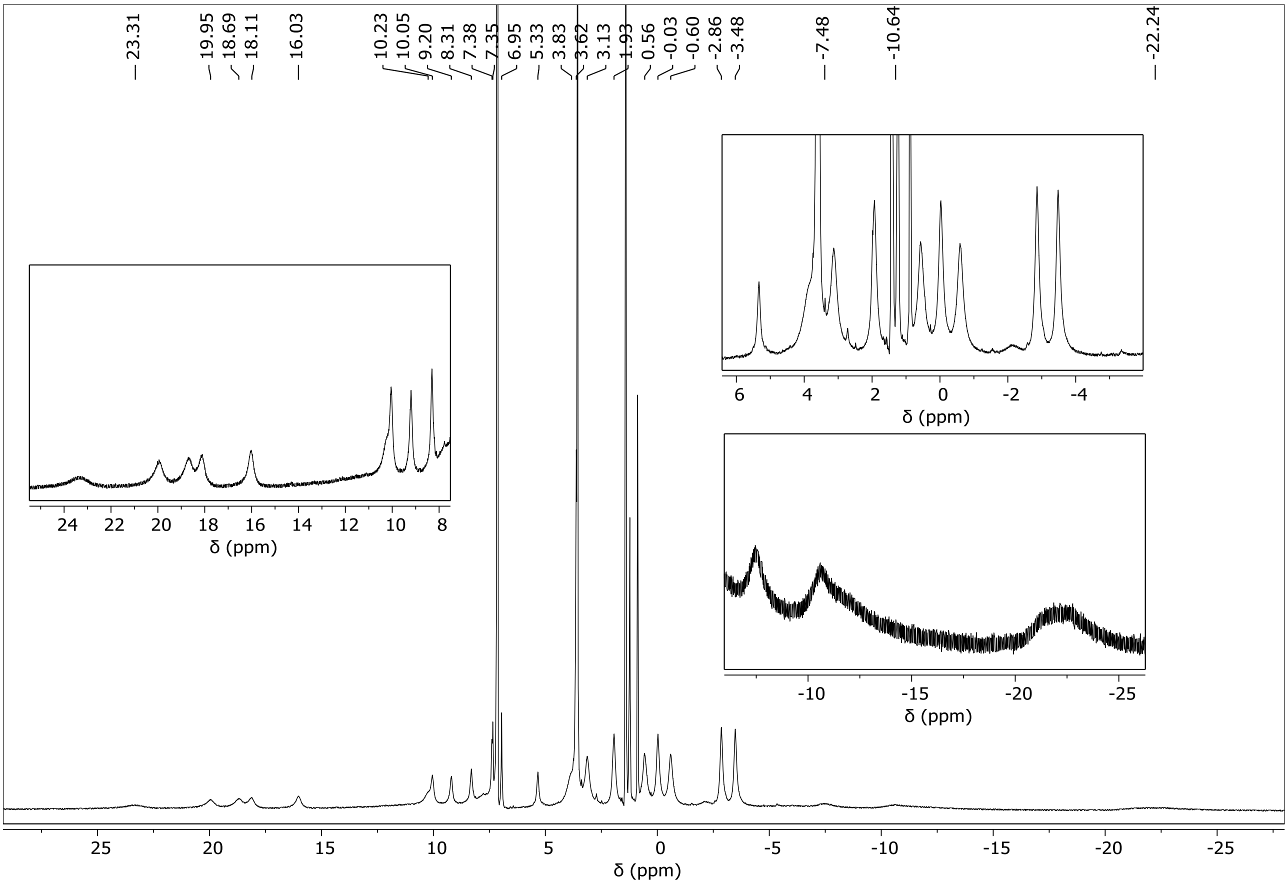

Supplementary Fig. 28. ^{1}H NMR spectrum of [(salNdipp)$_2$(DMAP)Os(NPCl)] (**5**) in C_6D_6 (400 MHz). The resonances at 1.24, and 0.89 ppm (hexane) and at 3.57, and 1.40 ppm (THF) arise from traces of solvent.

4.6 NMR Spectral Data for [(salNdipp)$_2$(DMAP)Os(N_4P)] (6)

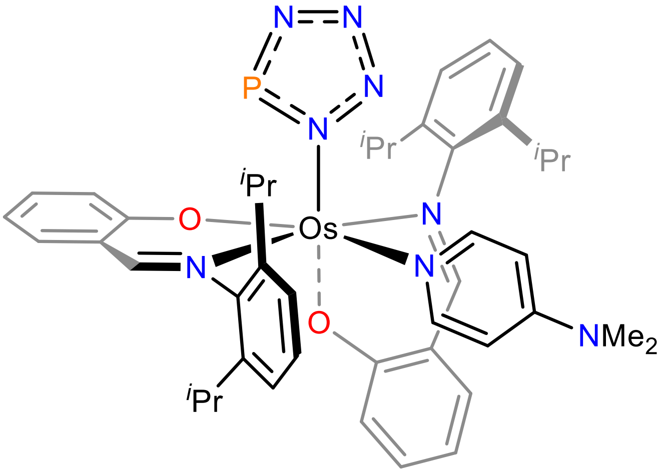

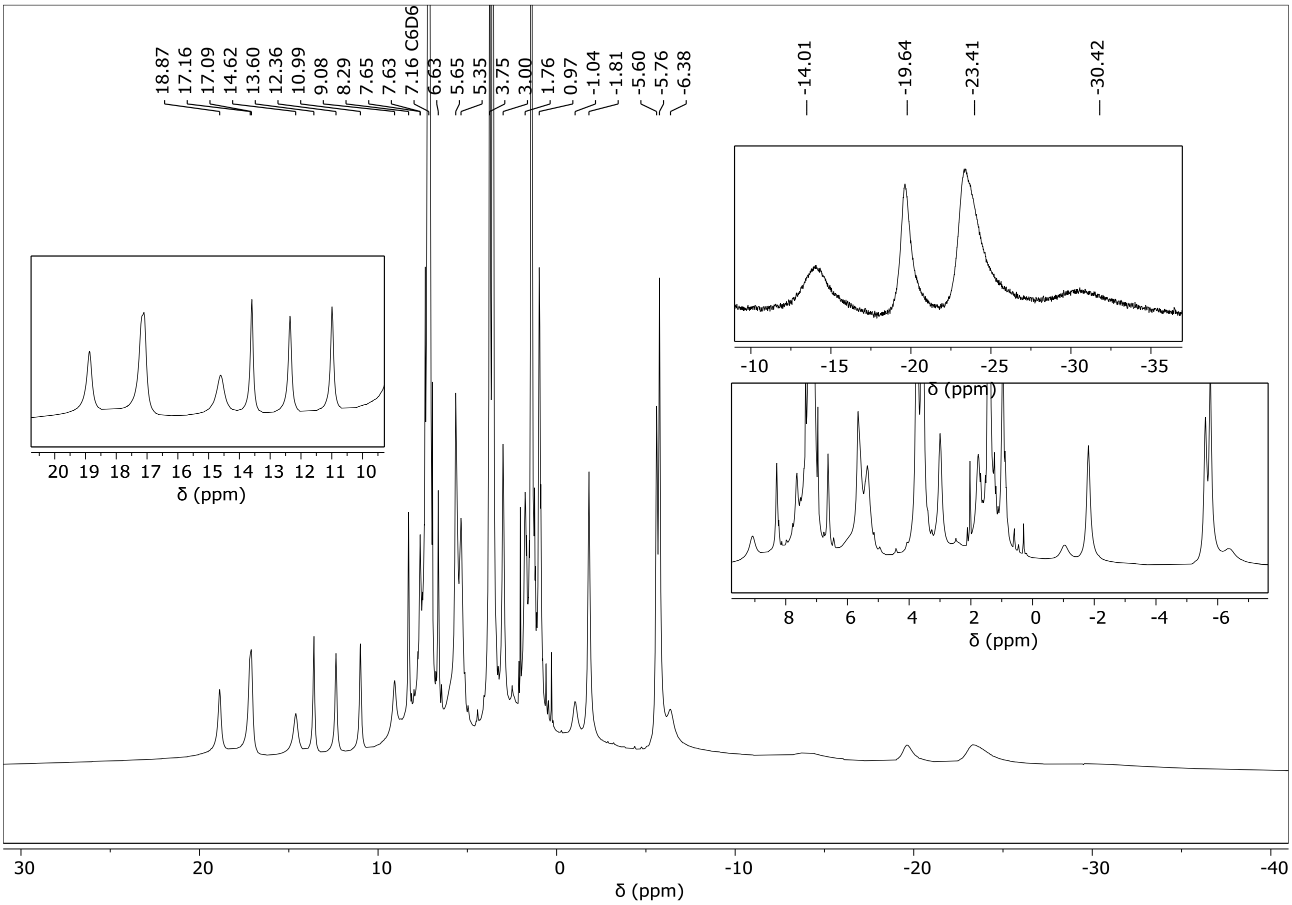

Supplementary Fig. 29. ^{1}H NMR spectrum of [(salNdipp)$_2$(DMAP)Os(N_4P)] (**6**) in C_6D_6 (400 MHz). The resonances at 3.57, and 1.40 ppm (THF) arise from traces of solvent.

5 IR Spectroscopy

5.1 IR Spectral Data for [(salNdipp)$_2$(DMAP)Os(NP)] (3)

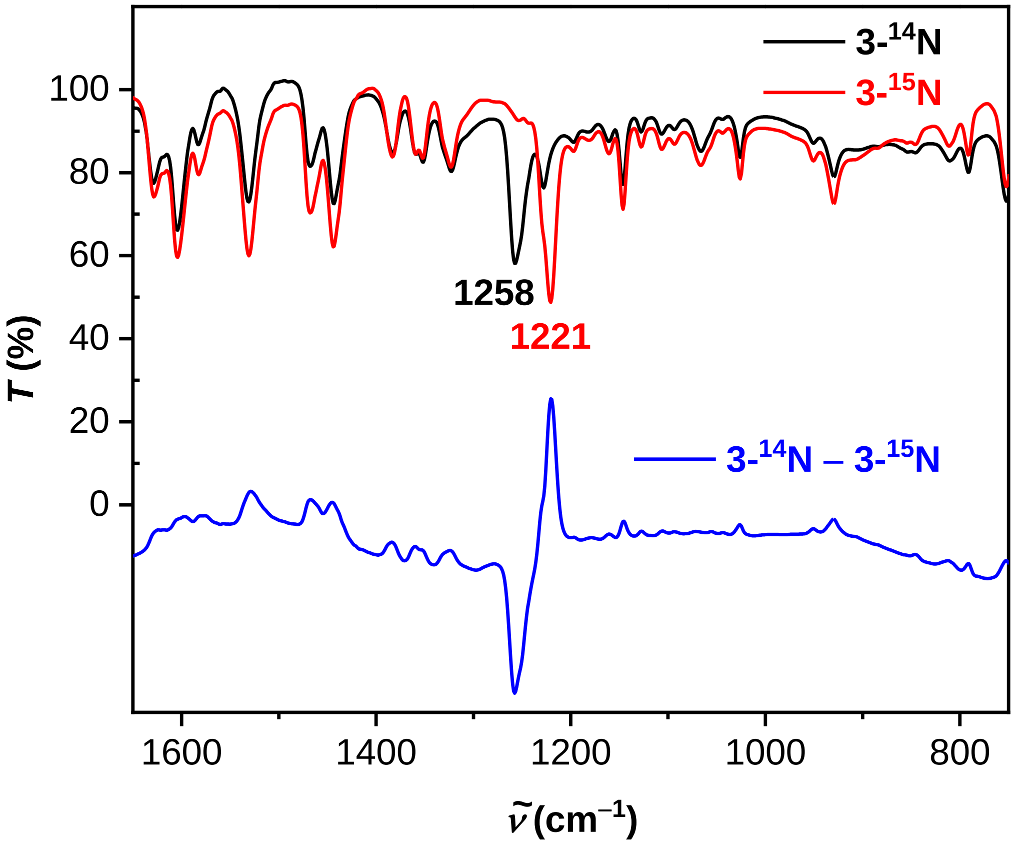

Supplementary Fig. 30. IR spectrum of solid [(salNdipp)$_2$(DMAP)Os(NP)] (**3**) and [(salNdipp)$_2$(DMAP)Os(^{15}NP)] (**3-^{15}N**).

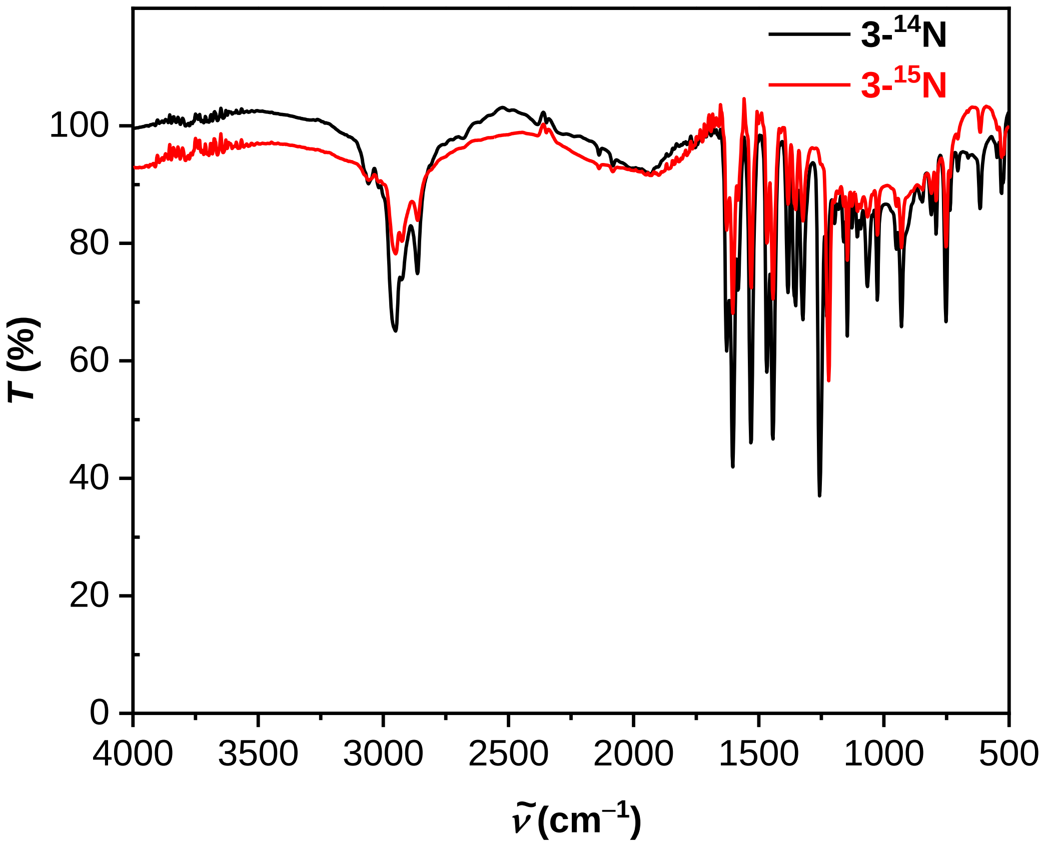

Supplementary Fig. 31. IR spectrum of solid [(salNdipp)$_2$(DMAP)Os(NP)] (**3**) and [(salNdipp)$_2$(DMAP)Os(NP)] (**3-^{15}N**).

5.2 IR Spectrum of [(salNdipp)$_2$(DMAP)Os(N_4P)] (6) treated with KC_8

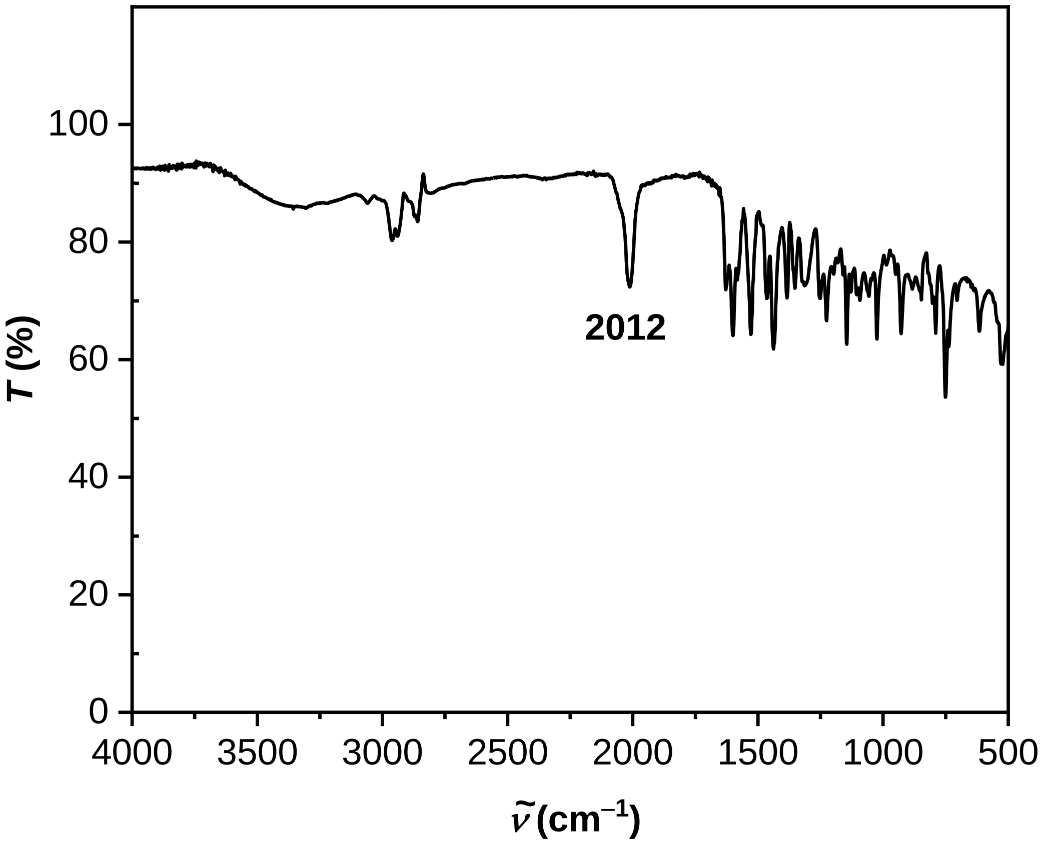

Supplementary Fig. 32. IR spectrum of reaction mixture from [(salNdipp)$_2$(DMAP)Os(N_4P)] (**6**) treated with KC_8 over 18h.

5.3 IR Spectrum of [(salNdipp)$_2$(OTf)Os(N)] (2) treated with Na(OCP).

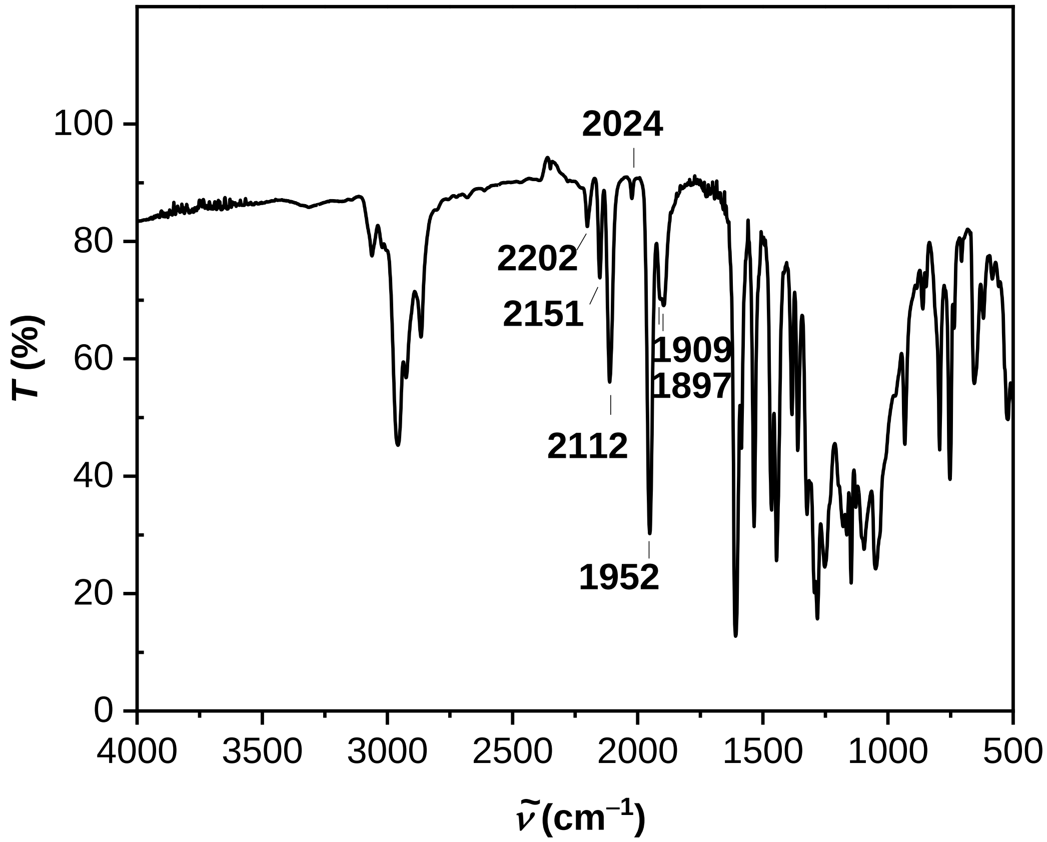

Supplementary Fig. 33. IR spectrum of the reaction mixture from the reaction between [(salNdipp)$_2$(OTf)Os(N)] (**2**) and Na(OCP) · 2.5 dioxane.

5.4 IR Spectrum of insoluble product from thermal decomposition of [(salNdipp)$_2$(DMAP)Os(N_4P)] (6).

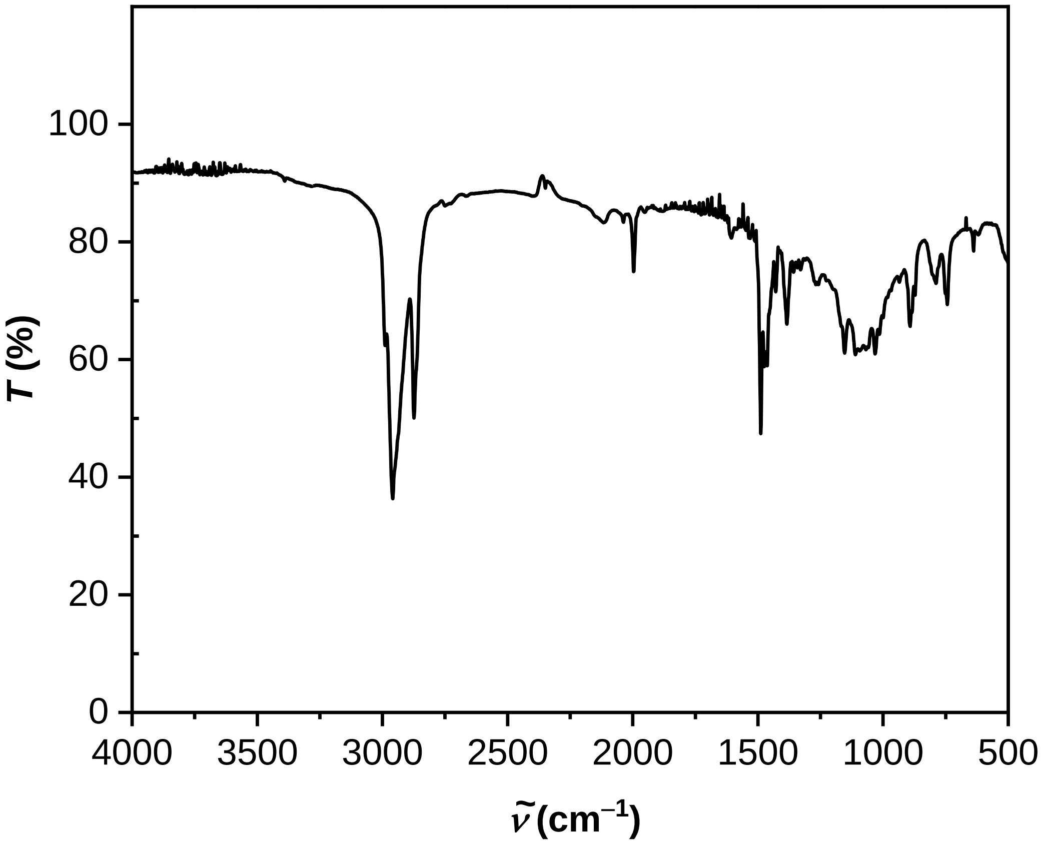

Supplementary Fig. 34. IR spectrum of insoluble product formed in the thermal decomposition of [(salNdipp)$_2$(DMAP)Os(N_4P)] (**6**).

5.5 IR Spectrum of products from thermal decomposition of [(salNdipp)$_2$(DMAP)Os(N_4P)] (6).

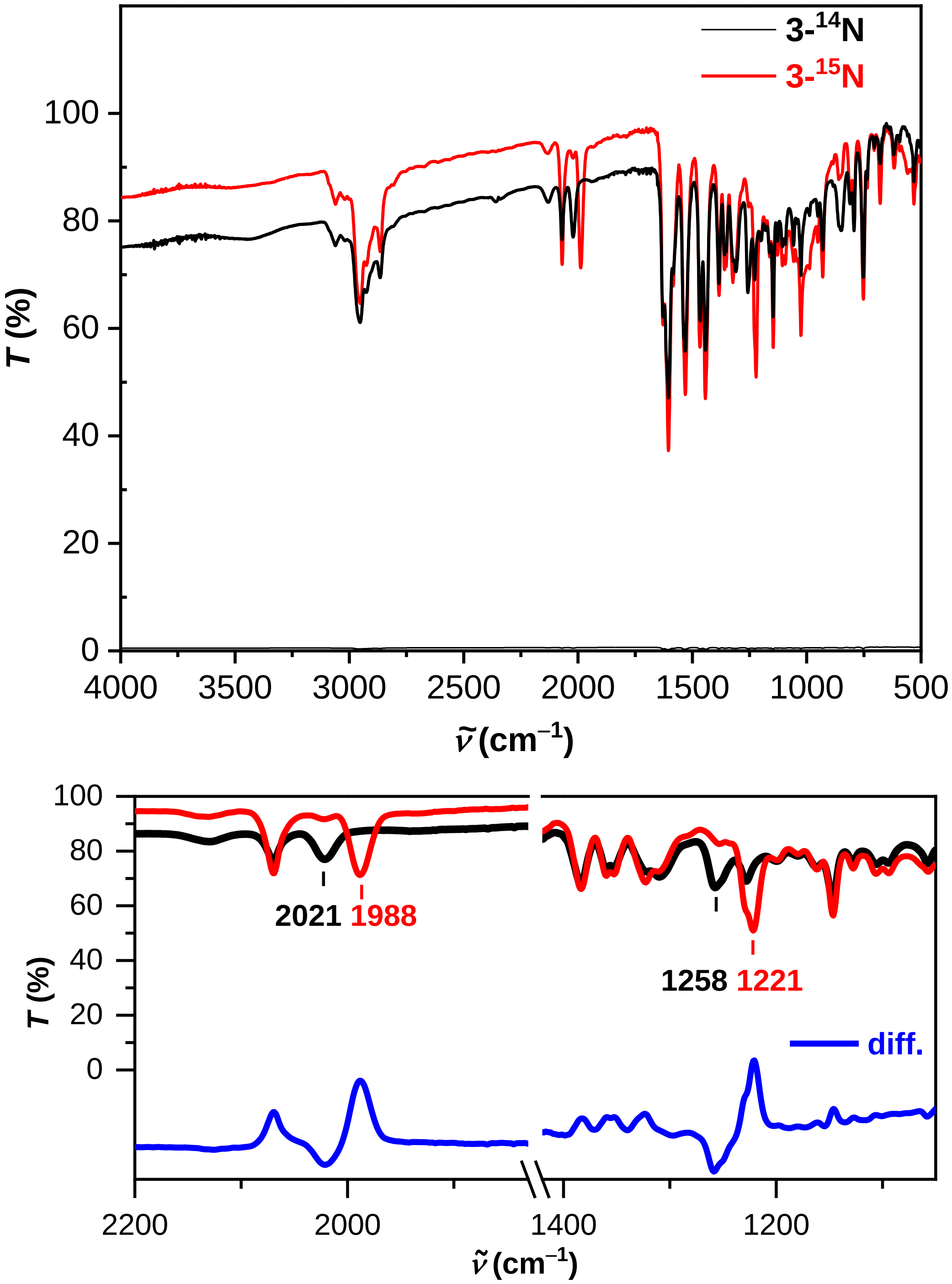

Supplementary Fig. 35. IR spectra of products formed upon heating **6/6-^{15}N** in C_6D_6, showing **3/3-^{15}N** at 1258/1221 cm^{-1} and **7/7-^{15}N** at 2021/1988 cm^{-1}. The isotopic shift for **7/7-^{15}N** agrees closely with the expected value for a $^{14}N{\equiv}^{14}N/^{15}N{\equiv}^{14}N$ harmonic oscillator (both shifting by 1.017).

6 UV-vis Spectroscopy

6.1 UV-vis Spectral Data for [(salNdipp)$_2$(OTf)Os(N)] (2)

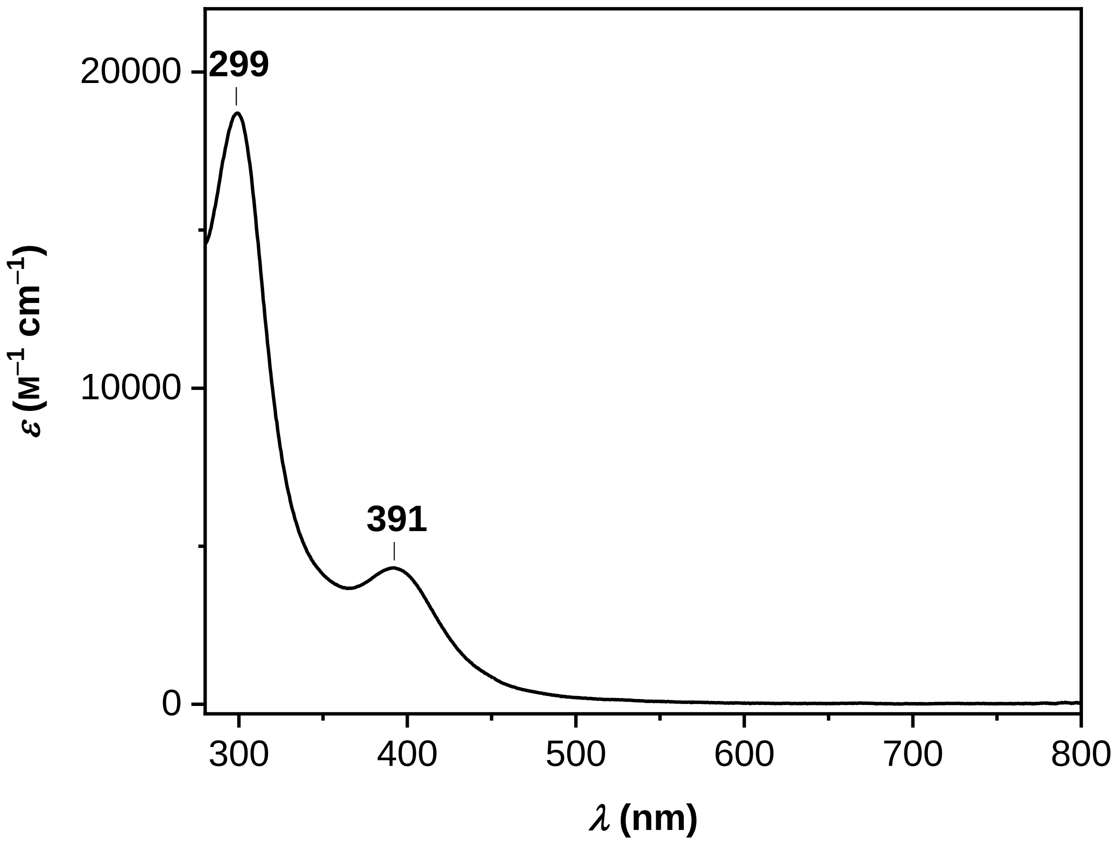

Supplementary Fig. 36. UV-vis spectrum of [(salNdipp)$_2$(OTf)Os(N)] (**2**) in THF. The above spectrum consists of data recorded at $4.94 \cdot 10^{-5}$ M.

6.2 UV-vis Spectral Data for [(salNdipp)$_2$(DMAP)Os(NP)] (3)

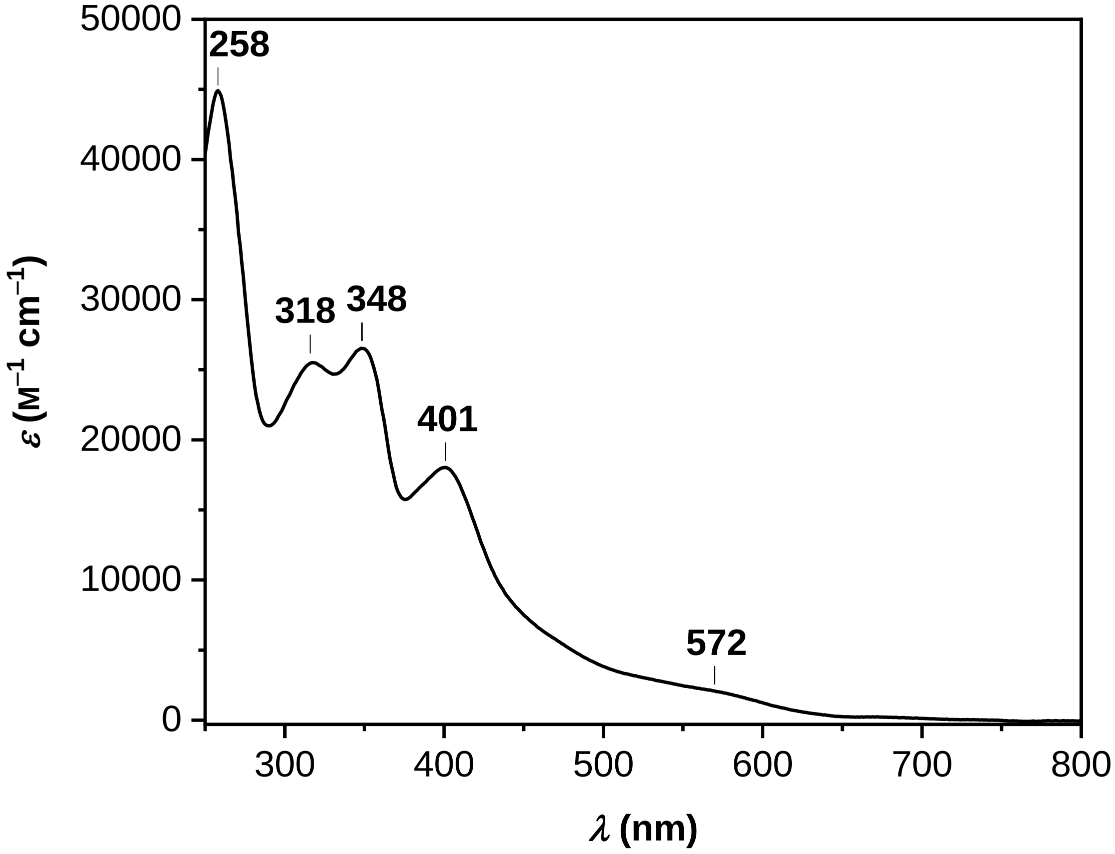

Supplementary Fig. 37. UV-vis spectrum of [(salNdipp)$_2$(DMAP)Os(NP)] (**3**) in THF. The above spectrum consists of data recorded at $2.28 \cdot 10^{-5}$ M.

6.3 UV-vis Spectral Data for [(salNdipp)$_2$(DMAP)Os(NPS$_2$)] (4)

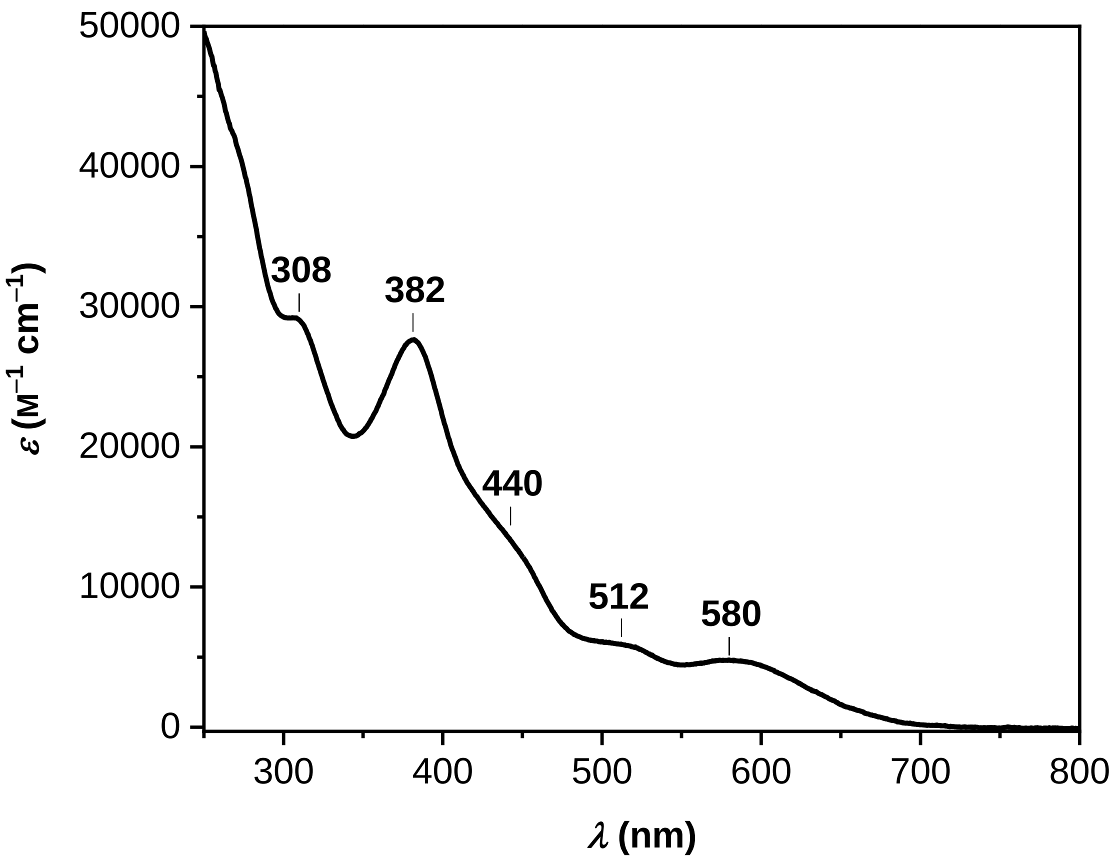

Supplementary Fig. 38. UV-vis spectrum of [(salNdipp)$_2$(DMAP)Os(NPS$_2$)] (**4**) in THF. The above spectrum consists of data recorded at $1.32 \cdot 10^{-5}$ M.

6.4 UV-vis Spectral Data for [(salNdipp)$_2$(DMAP)Os(NPCl)] (5)

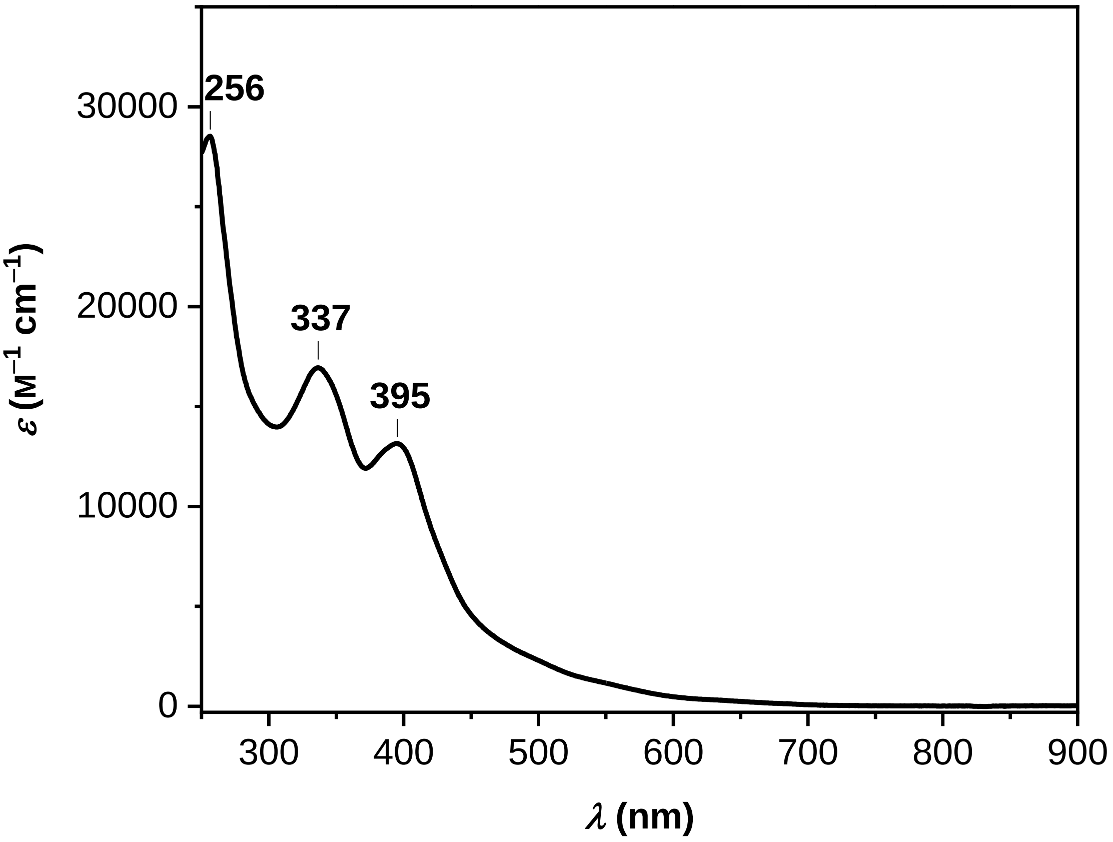

Supplementary Fig. 39. UV-vis spectrum of [(salNdipp)$_2$(DMAP)Os(NPCl)] (**5**) in THF. The above spectrum consists of data recorded at 6.43 · 10^{-5} M.

6.5 UV-vis Spectral Data for [(salNdipp)$_2$(DMAP)Os(N_4P)] (6)

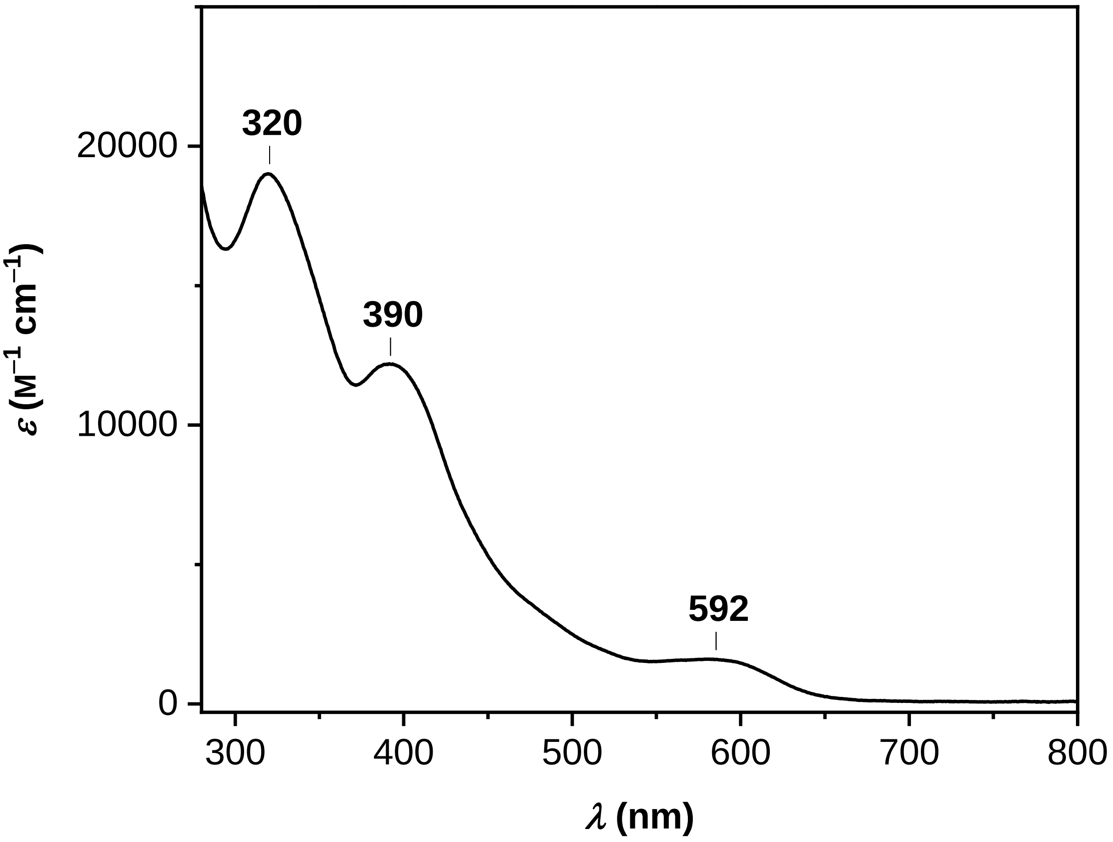

Supplementary Fig. 40. UV-vis spectrum of [(salNdipp)$_2$(DMAP)Os(N_4P)] (**6**) in THF. The above spectrum consists of data recorded at 5.71 · 10^{-5} M.

7 Reactivity Studies

7.1 Reaction between [(salNdipp)$_2$(OTf)Os(N)] (2) and Na(OCP).

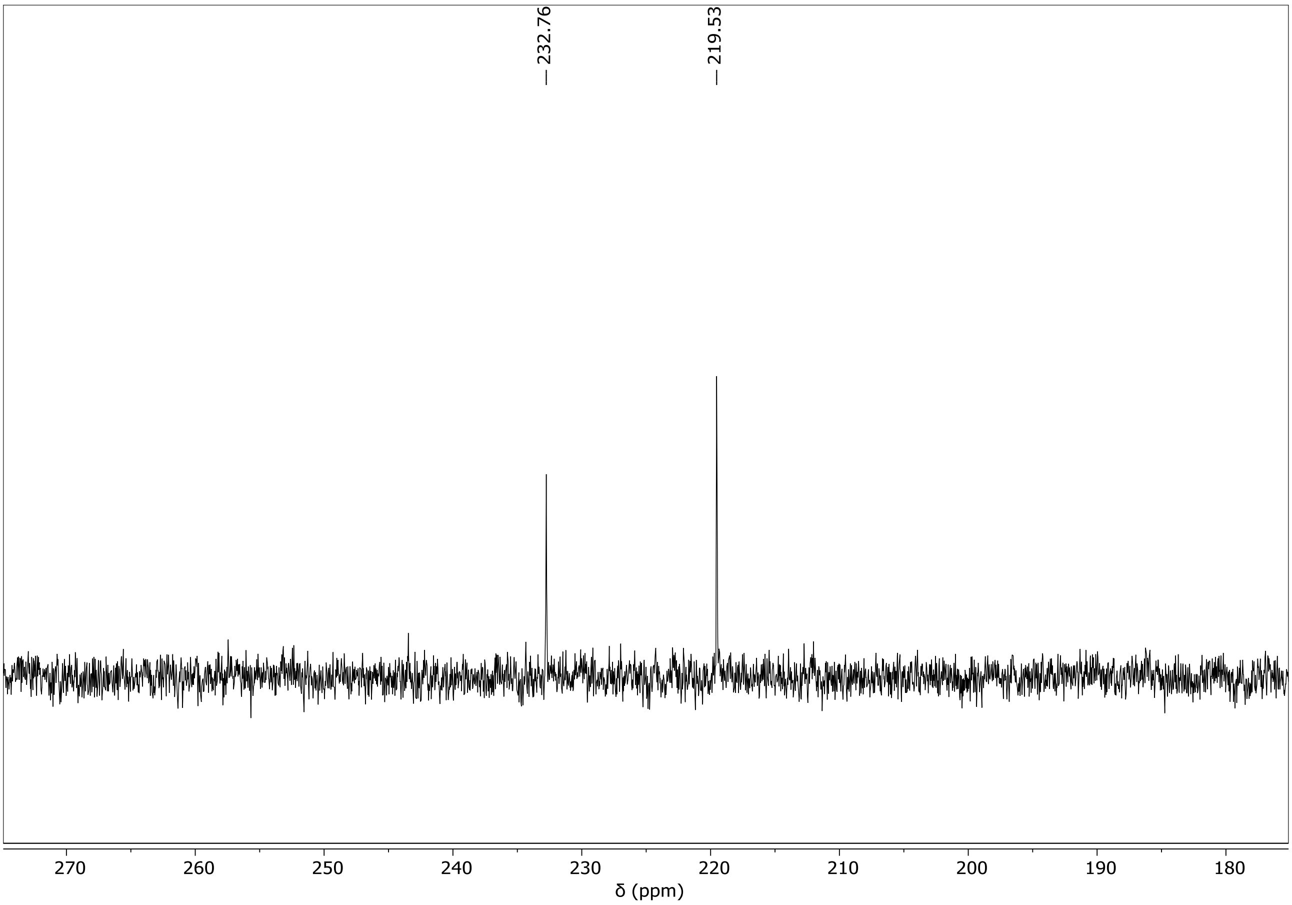

Supplementary Fig. 41. $^{31}P\{^{1}H\}$ NMR spectrum from the reaction mixture of [(salNdipp)$_2$(OTf)OsN] (**2**) and Na(OCP) · 2.5 dioxane in C_6D_6.

7.2 Sulfur atom transfer between [(salNdipp)$_2$(DMAP)Os(^{15}NP)] (3-^{15}N) and [(salNdipp)$_2$(DMAP)Os(NPS$_2$)] (4).

Procedure: 3.0 mg of [(salNdipp)$_2$(DMAP)Os(^{15}NP)] (**3-^{15}N**, 3.3 µmol) and 3.3 mg of [(salNdipp)$_2$(DMAP)Os(NPS$_2$)] (**4**, 3.4 µmol) were dissolved in 0.5 ml THF-d$_8$, and the reaction mixture was analyzed by ^{31}P{^{1}H} NMR spectroscopy.

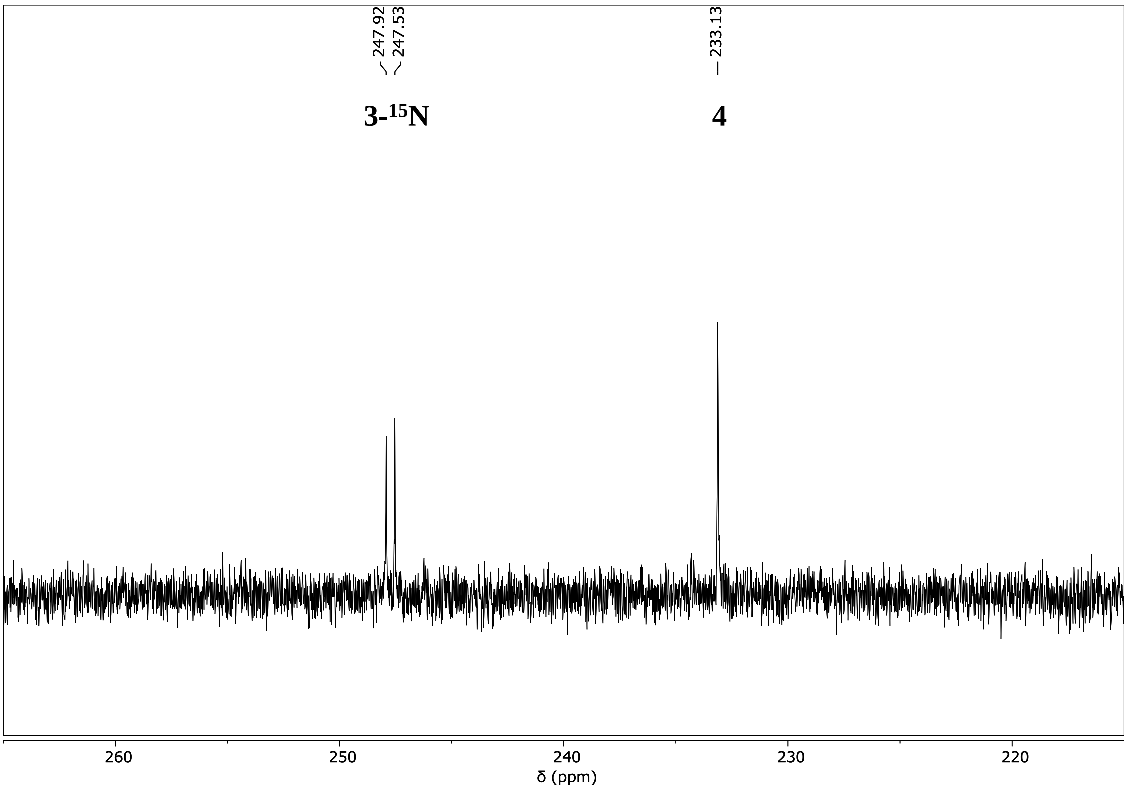

Supplementary Fig. 42. ^{31}P{^{1}H} NMR spectrum of [(salNdipp)$_2$(DMAP)Os(^{15}NP)] (**3-^{15}N**) and [(salNdipp)$_2$(DMAP)Os(NPS$_2$)] (**4**) in THF-d$_8$ after dissolution at room temperature.

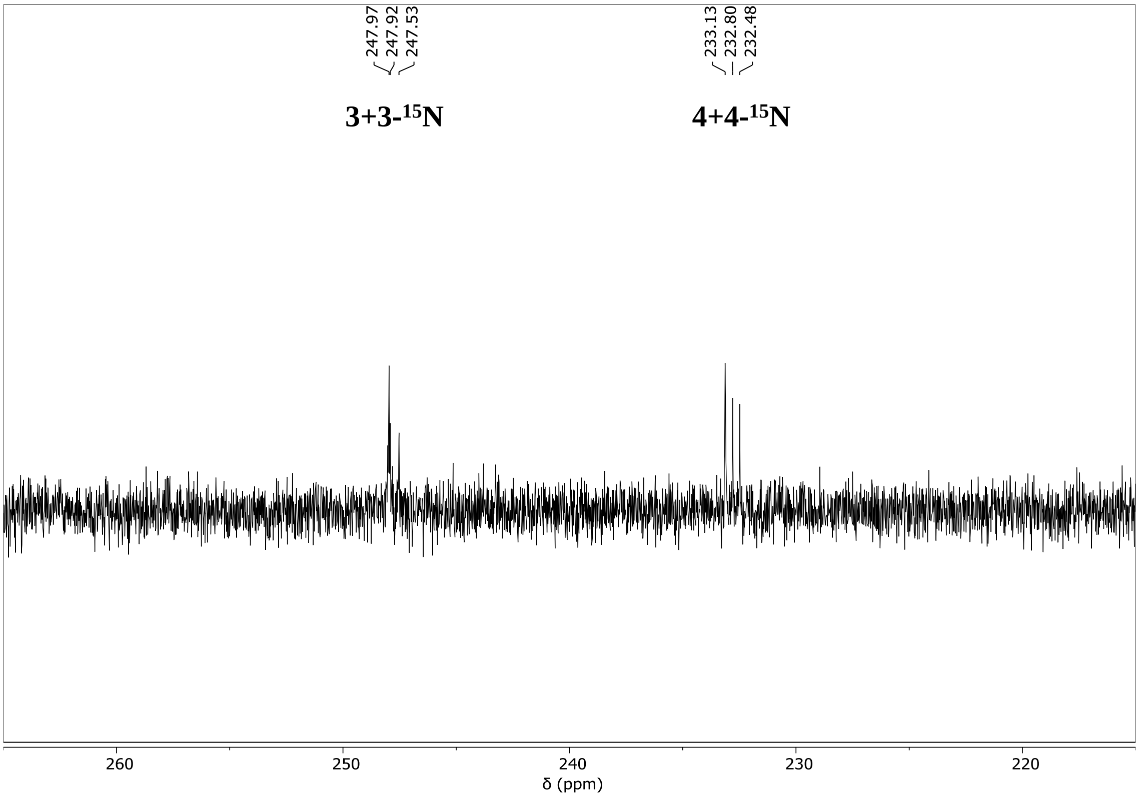

Supplementary Fig. 43. $^{31}P\{^{1}H\}$ NMR spectrum of [(salNdipp)$_2$(DMAP)Os(^{15}NP)] (**3-^{15}N**) and [(salNdipp)$_2$(DMAP)Os(NPS$_2$)] (**4**) in THF-d$_8$ after being heated to 50 °C for 48 hours. Sulfur atom transfer results in peaks from **3** and **3-^{15}N** as well as **4** and **4-^{15}N**.

7.3 Desulfurization of [(salNdipp)$_2$(DMAP)Os(NPS$_2$)] (4) using PPh$_3$

Procedure: 2.5 mg of [(salNdipp)$_2$(DMAP)Os(NPS$_2$)] (**4**, 2.5 µmol) and 3.1 mg PPh$_3$ (12 µmol) were partially dissolved in 0.5 ml C_6D_6, and the reaction mixture was analyzed by ^{31}P{^{1}H} NMR spectroscopy.

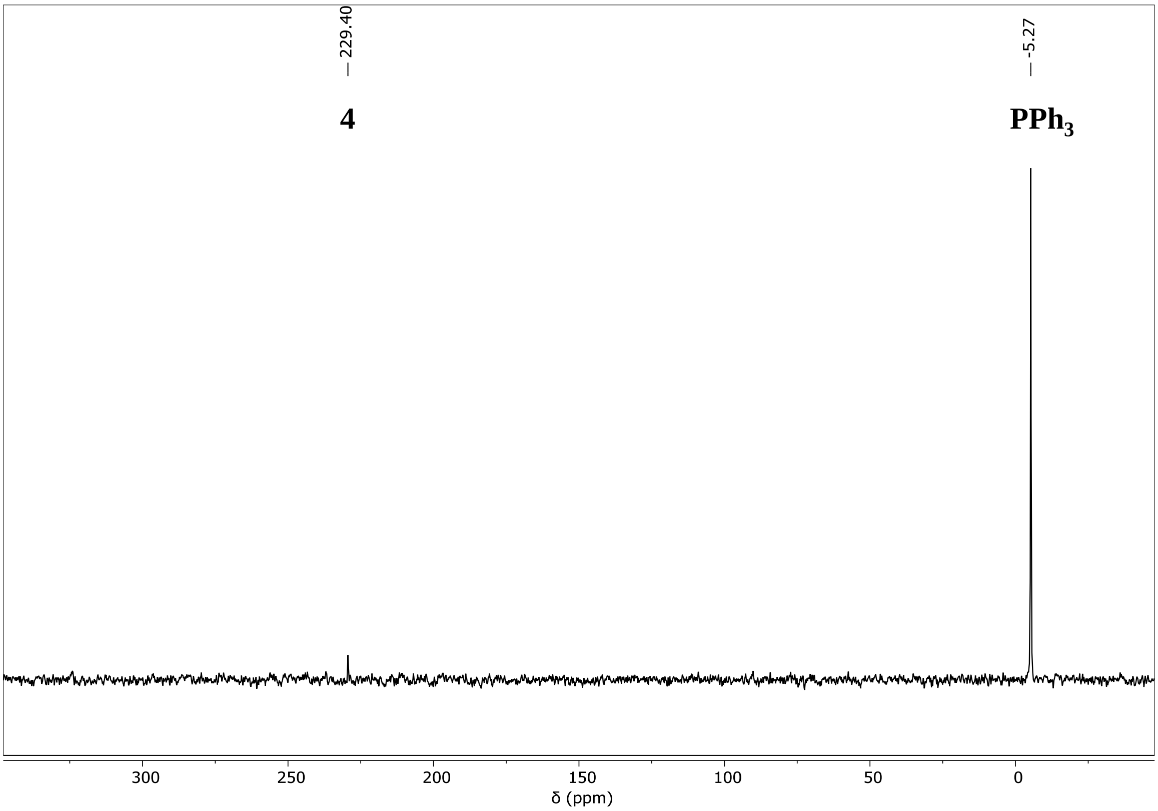

Supplementary Fig. 44. ^{31}P{^{1}H} NMR spectrum of [(salNdipp)$_2$(DMAP)Os(NPS$_2$)] (**4**) and PPh$_3$ in C_6D_6 after dissolution at room temperature.

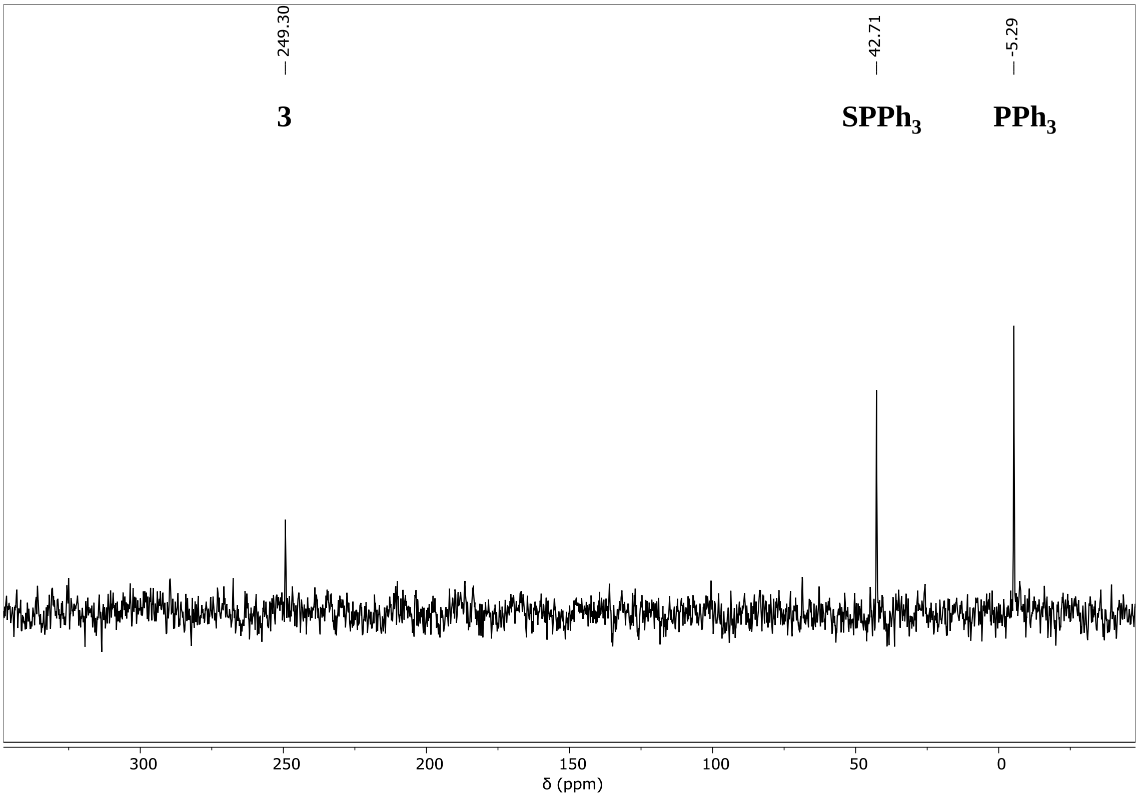

Supplementary Fig. 45. $^{31}P\{^1H\}$ NMR spectrum of [(salNdipp)$_2$(DMAP)Os(NPS$_2$)] (**4**) and PPh_3 in C_6D_6 after being heated to 50 °C for 24 hours. Desulfurization results in peaks of **3** and $SPPh_3$.

7.4 **Thermal decomposition of [(salNdipp)$_2$(DMAP)Os(N_4P)] (6)**

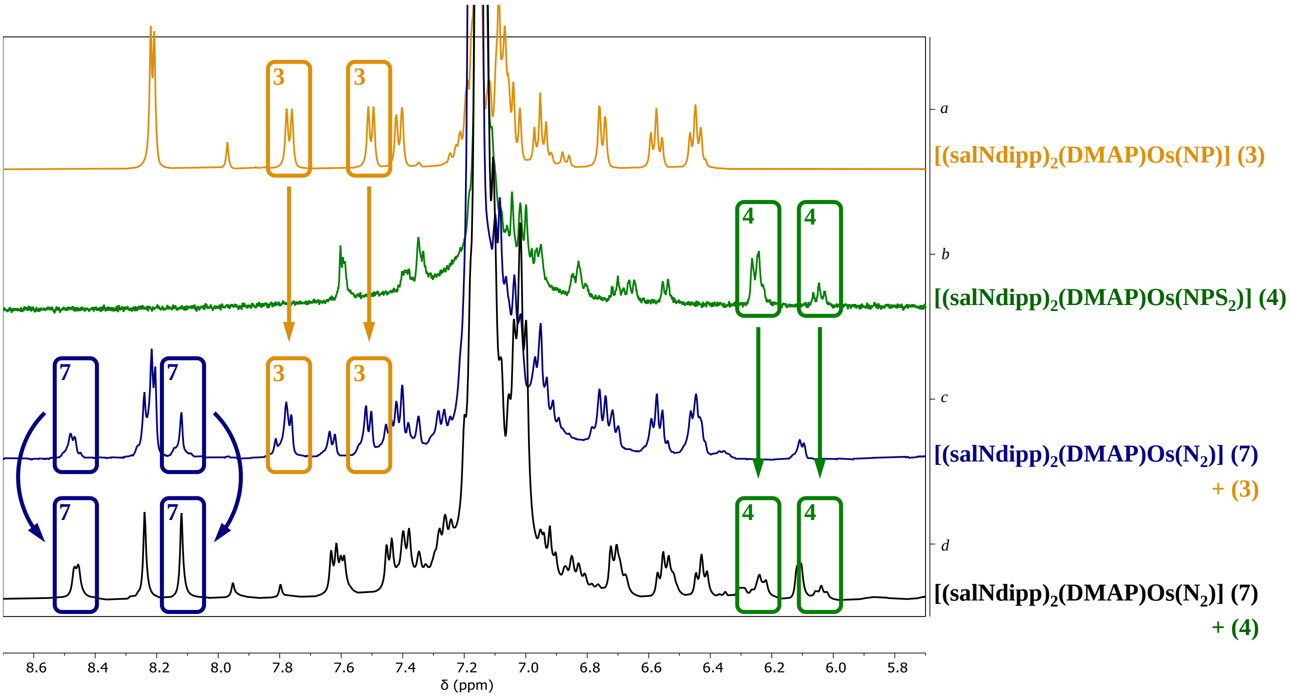

Supplementary Fig. 46. Stacked ^{1}H NMR spectra of:

[a] [(salNdipp)$_2$(DMAP)Os(NP)] (**3**, **Orange**),

[b] [(salNdipp)$_2$(DMAP)Os(NPS_2)] (**4**, **Green**),

[c] [(salNdipp)$_2$(DMAP)Os(N_4P)] (**6**, **Blue**) in C_6D_6 after being heated to 50 °C for 8 hours (showing a 1:1 mixture of **3** and **7**),

[d] [(salNdipp)$_2$(DMAP)Os(N_4P)] (**6**, **Black**) in C_6D_6 after being heated to 50 °C for 8 hours and reacted with sulfur (showing a 1:1 mixture of **4** and **7**).

8 Crystallographic Data

8.1 Crystallographic Tables

Supplementary Table 1. Crystallographic data for complex **1** as a toluene solvate and complex **2**.

**Complex**<br>**CCDC entry**	[(salNdipp)$_2$(Cl)Os≡N] **(1)**<br>**2388544**	[(salNdipp)$_2$(OTf)Os≡N] **(2)**<br>**2388542**
Empirical formula	$C_{45}H_{52}ClN_3O_2Os$	$C_{39}H_{44}F_3N_3O_5OsS$
Formula weight	892.54	914.03
Temperature / K	100(2)	100(2)
Crystal system	monoclinic	triclinic
Spacegroup	$P2_1/c$	P–1
a / Å	10.4517(2)	12.6896(4)
b / Å	19.8822(3)	17.3558(5)
c / Å	19.9294(3)	19.5680(5)
α / °	90	91.264(2)
β / °	104.712(2)	100.567(2)
γ / °	90	103.866(2)
V / Å^3	4005.61(12)	4103.2(2)
Z	4	4
ρ_{calc} g/cm^3	1.480	1.480
μ / mm^{-1}	3.291	3.215
F(000)	1808.0	1832.0
Crystal size / mm^3	0.243 × 0.152 × 0.025	0.129 × 0.118 × 0.06
Radiation (Å)	MoK_α (λ = 0.71073)	MoK_α (λ = 0.71073)
2θ range / °	6.664 – 56.652	6.55 – 50.826
Index ranges	$-13 \le h \le 13$, $-26 \le k \le 26$, $-26 \le l \le 26$	$-15 \le h \le 15$, $-20 \le k \le 20$, $-23 \le l \le 23$
Reflections collected	235851	121247
Independent reflections	9941 [R_{int} = 0.1449, R_{sigma} = 0.0481]	15066 [R_{int} = 0.1681, R_{sigma} = 0.1211]
Data / restraints / parameters	9941 / 0 / 478	15066 / 12 / 953
Goodness-of-fit on F^2	1.034	1.018
Final R indexes [$I \ge 2\sigma(I)$]	R_1 = 0.0336, wR_2 = 0.0648	R_1 = 0.0646, wR_2 = 0.1373
Final R indexes [all data]	R_1 = 0.0649, wR_2 = 0.0759	R_1 = 0.1265, wR_2 = 0.1632
Largest diff. peak/hole / e Å^{-3}	2.37 / –1.04	4.87 / –1.25

Supplementary Table 2. Crystallographic data for complexes **3**, **4** as toluene solvates.

**Complex**<br>**CCDC entry**	[(salNdipp)$_2$(DMAP)Os(NP)] **(3)**<br>**2388539**	[(salNdipp)$_2$(DMAP)Os(NPS$_2$)] **(4)**<br>**2388543**
Empirical formula	$C_{48.5}H_{57.5}N_5O_2OsP$	$C_{55.5}H_{62}N_5O_2OsPS_2$
Formula weight	963.66	1116.39
Temperature / K	100(2)	100(2)
Crystal system	triclinic	monoclinic
Spacegroup	P–1	$P2_1/c$
a / Å	11.7080(3)	20.8588(5)
b / Å	12.9028(3)	11.8348(2)
c / Å	20.1856(5)	22.5214(5)
α / °	74.061(2)	90
β / °	82.537(2)	109.278(2)
γ / °	68.180(2)	90
V / Å^3	2720.73(12)	5247.9(2)
Z	2	4
ρ_{calc} g/cm^3	1.176	1.413
μ / mm^{-1}	2.409	2.585
F(000)	981.0	2276.0
Crystal size / mm^3	0.352 × 0.173 × 0.093	0.443 × 0.144 × 0.06
Radiation (Å)	MoK_α (λ = 0.71073)	MoK_α (λ = 0.71073)
2θ range / °	6.668 – 50.732	6.702 – 52.784
Index ranges	$-14 \le h \le 14$, $-15 \le k \le 15$, $-24 \le l \le 24$	$-26 \le h \le 26$, $-14 \le k \le 14$, $-28 \le l \le 28$
Reflections collected	51980	133698
Independent reflections	9959 [R_{int} = 0.0671, R_{sigma} = 0.0541]	10745 [R_{int} = 0.1103, R_{sigma} = 0.0513]
Data / restraints / parameters	9959 / 6 / 514	10745 / 0 / 599
Goodness-of-fit on F^2	1.045	1.021
Final R indexes [$I \ge 2\sigma(I)$]	R_1 = 0.0439, wR_2 = 0.1073	R_1 = 0.0346, wR_2 = 0.0655
Final R indexes [all data]	R_1 = 0.0526, wR_2 = 0.1118	R_1 = 0.0604, wR_2 = 0.0745
Largest diff. peak/hole / e Å^{-3}	2.81 / –1.09	1.43 / –0.93

Supplementary Table 3. Crystallographic data for complexes **5**, **6**.

**Complex**<br>**CCDC entry**	[(salNdipp)$_2$(DMAP)Os(NPCl)] **(5)**<br>**2388541**	[(salNdipp)$_2$(DMAP)Os(N$_4$P)] **(6)**<br>**2388540**
Empirical formula	$C_{45}H_{54}ClN_5O_2OsP$	$C_{45}H_{54}N_8O_2OsP$
Formula weight	953.55	960.13
Temperature / K	100(2)	100(2)
Crystal system	monoclinic	monoclinic
Spacegroup	$P2_1/n$	$P2_1/n$
a / Å	14.9374(3)	13.3318(5)
b / Å	21.8926(5)	15.0163(5)
c / Å	15.8131(4)	22.7199(10)
α / °	90	90
β / °	105.943(2)	93.921(4)
γ / °	90	90
V / Å^3	4972.3(2)	4537.7(3)
Z	4	4
ρ_{calc} g/cm^3	1.274	1.405
μ / mm^{-1}	2.687	2.890
F(000)	1932.0	1948.0
Crystal size / mm^3	0.197 × 0.123 × 0.053	0.293 × 0.06 × 0.036
Radiation (Å)	MoK_α (λ = 0.71073)	MoK_α (λ = 0.71073)
2θ range / °	6.526 – 50.748	6.51 – 49.426
Index ranges	$-18 \le h \le 17$, $-26 \le k \le 26$, $-19 \le l \le 19$	$-15 \le h \le 15$, $-17 \le k \le 17$, $-26 \le l \le 26$
Reflections collected	105958	84882
Independent reflections	9102 [R_{int} = 0.1212, R_{sigma} = 0.0655]	7724 [R_{int} = 0.2489, R_{sigma} = 0.1259]
Data / restraints / parameters	9102 / 0 / 506	7724 / 6 / 524
Goodness-of-fit on F^2	1.004	0.964
Final R indexes [$I \ge 2\sigma(I)$]	R_1 = 0.0406, wR_2 = 0.0792	R_1 = 0.0553, wR_2 = 0.1007
Final R indexes [all data]	R_1 = 0.0767, wR_2 = 0.0890	R_1 = 0.1242, wR_2 = 0.1231
Largest diff. peak/hole / e Å^{-3}	1.58 / –0.55	1.36 / –0.71

Supplementary Table 4. Crystallographic data for complex **7**.

**Complex**<br>**CCDC entry**	[(salNdipp)$_2$(DMAP)Os(N$_2$)] **(7)**<br>**2388538**
Empirical formula	$C_{45}H_{54}N_6O_2Os$
Formula weight	901.14
Temperature / K	100(2)
Crystal system	triclinic
Spacegroup	P–1
a / Å	11.6638(3)
b / Å	12.7773(4)
c / Å	20.0015(7)
α / °	76.502(3)
β / °	84.182(3)
γ / °	67.648(3)
V / Å^3	2680.48(16)
Z	2
ρ_{calc} g/cm^3	1.116
μ / mm^{-1}	2.413
F(000)	916.0
Crystal size / mm^3	0.34 × 0.16 × 0.09
Radiation (Å)	MoK_α (λ = 0.71073)
2θ range / °	6.622 – 56.674
Index ranges	$-15 \le h \le 15$, $-17 \le k \le 17$, $-26 \le l \le 26$
Reflections collected	51489
Independent reflections	13283 [R_{int} = 0.0916, R_{sigma} = 0.1040]
Data / restraints / parameters	13283 / 6 / 497
Goodness-of-fit on F^2	1.005
Final R indexes [$I \ge 2\sigma(I)$]	R_1 = 0.0567, wR_2 = 0.1271
Final R indexes [all data]	R_1 = 0.0825, wR_2 = 0.1371
Largest diff. peak/hole / e Å^{-3}	2.72 / –1.70

8.2 Thermal Ellipsoid Plot of [(salNdipp)$_2$(Cl)Os≡N] (1)

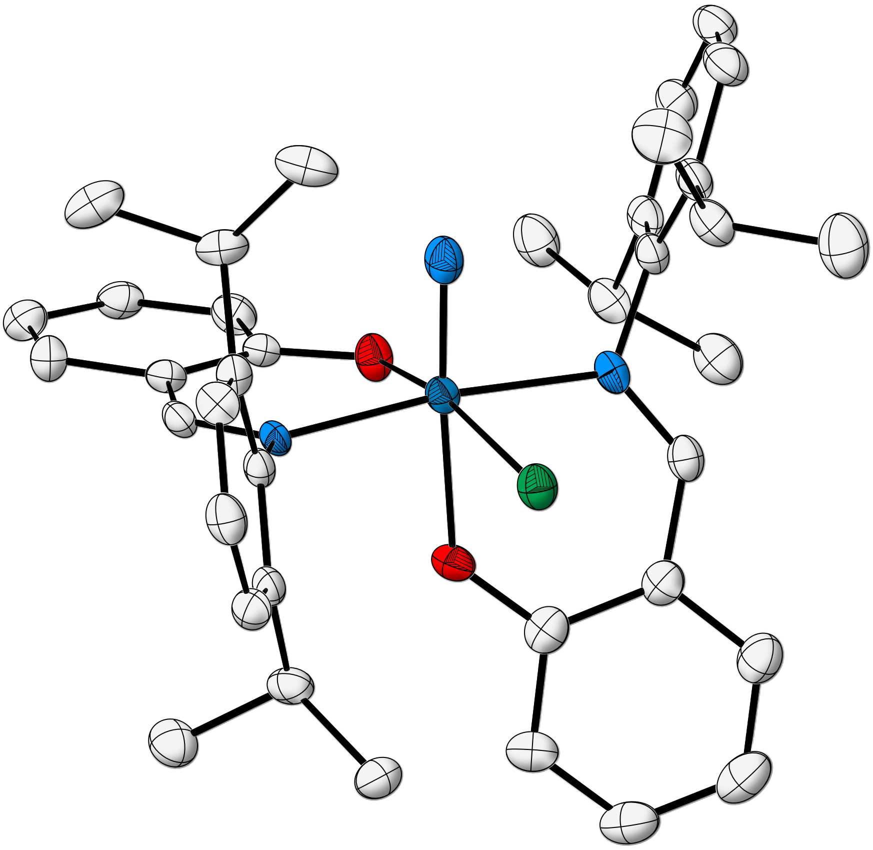

Supplementary Fig. 47. ORTEP plot of [(salNdipp)$_2$(Cl)Os≡N] (**1**), CCDC entry **2388544**. The diffraction pattern was recorded at 100(2) K, and the thermal ellipsoids are set to 50% probability. H-atoms and co-crystallized toluene are omitted. Color code: Light gray (C), light blue (N), red (O), green (Cl), turquoise (Os).

8.3 Thermal Ellipsoid Plot of [(salNdipp)$_2$(OTf)Os≡N] (2)

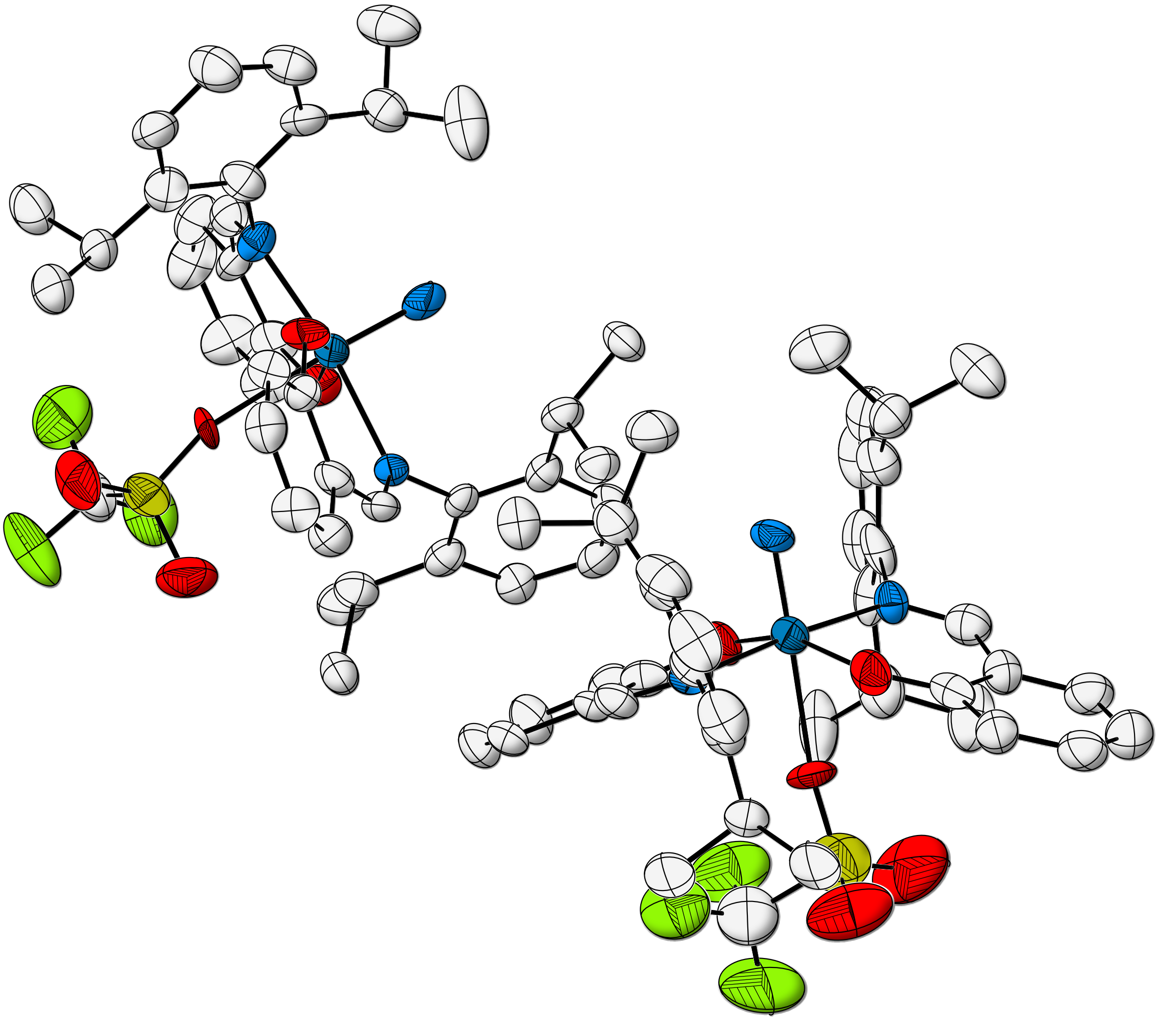

Supplementary Fig. 48. ORTEP plot showing two crystallographically independent units of [(salNdipp)$_2$(OTf)Os≡N] (**2**), CCDC entry **2388542**. The diffraction pattern was recorded at 100(2) K, and the thermal ellipsoids are set to 50% probability. H-atoms are omitted. Color code: Light gray (C), light blue (N), red (O), light green (F), dark yellow (S), turquoise (Os).

8.4 Thermal Ellipsoid Plot of [(salNdipp)$_2$(DMAP)Os(NP)] (3)

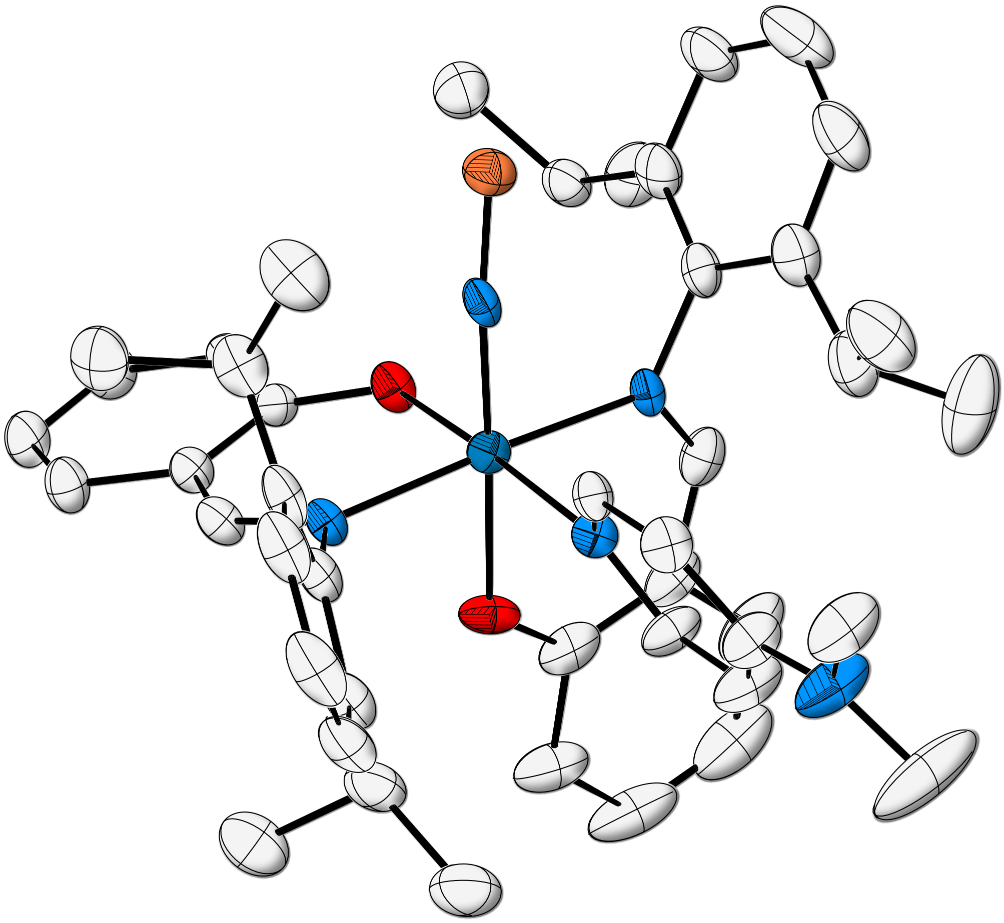

Supplementary Fig. 49. ORTEP plot of [(salNdipp)$_2$(DMAP)Os(NP)] (**3**), CCDC entry **2388539**. The diffraction pattern was recorded at 100(2) K, and the thermal ellipsoids are set to 50% probability. H-atoms and co-crystallized toluene are omitted. Color code: Light gray (C), light blue (N), red (O), orange (P), turquoise (Os).

8.5 Thermal Ellipsoid Plot of [(salNdipp)$_2$(DMAP)Os(NPS$_2$)] (4)

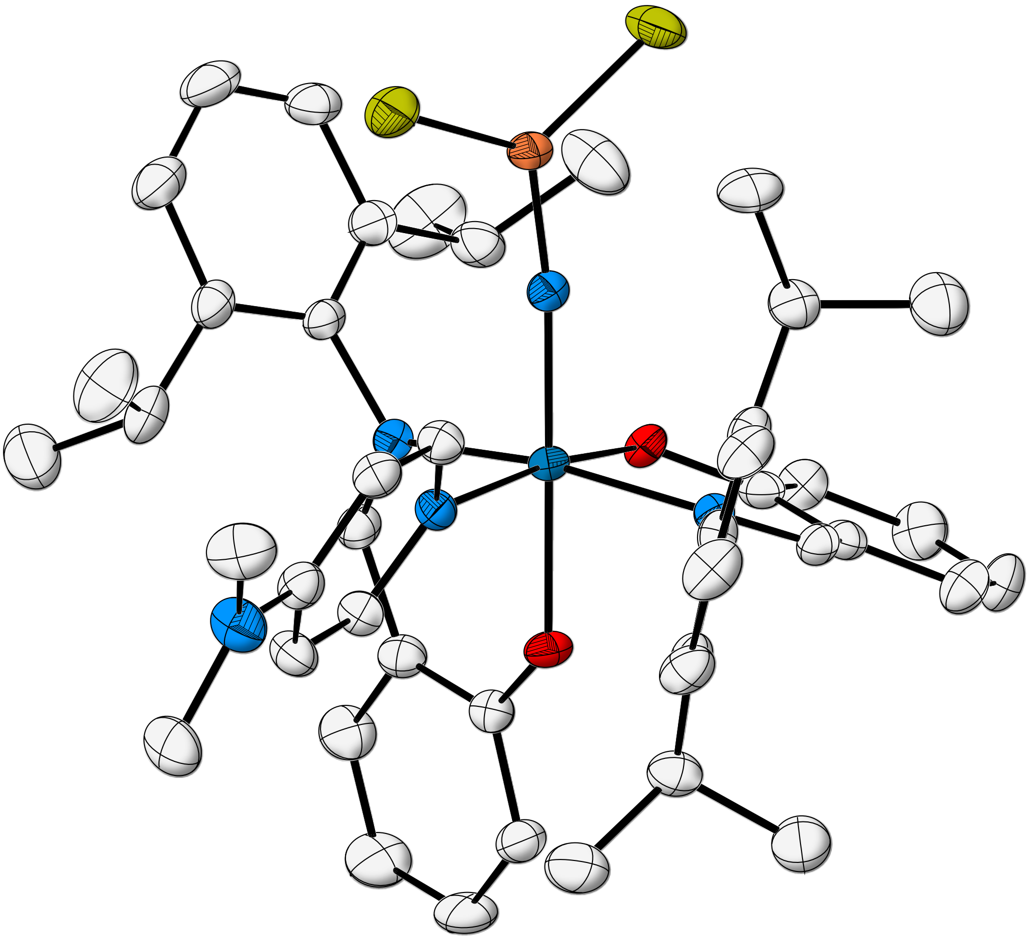

Supplementary Fig. 50. ORTEP plot of [(salNdipp)$_2$(DMAP)Os(NPS$_2$)] (**4**), CCDC entry **2388543**. The diffraction pattern was recorded at 100(2) K, and the thermal ellipsoids are set to 50% probability. H-atoms and co-crystallized toluene are omitted. Color code: Light gray (C), light blue (N), red (O), orange (P), dark yellow (S), turquoise (Os).

8.6 Thermal Ellipsoid Plot of [(salNdipp)$_2$(DMAP)Os(NPCl)] (5)

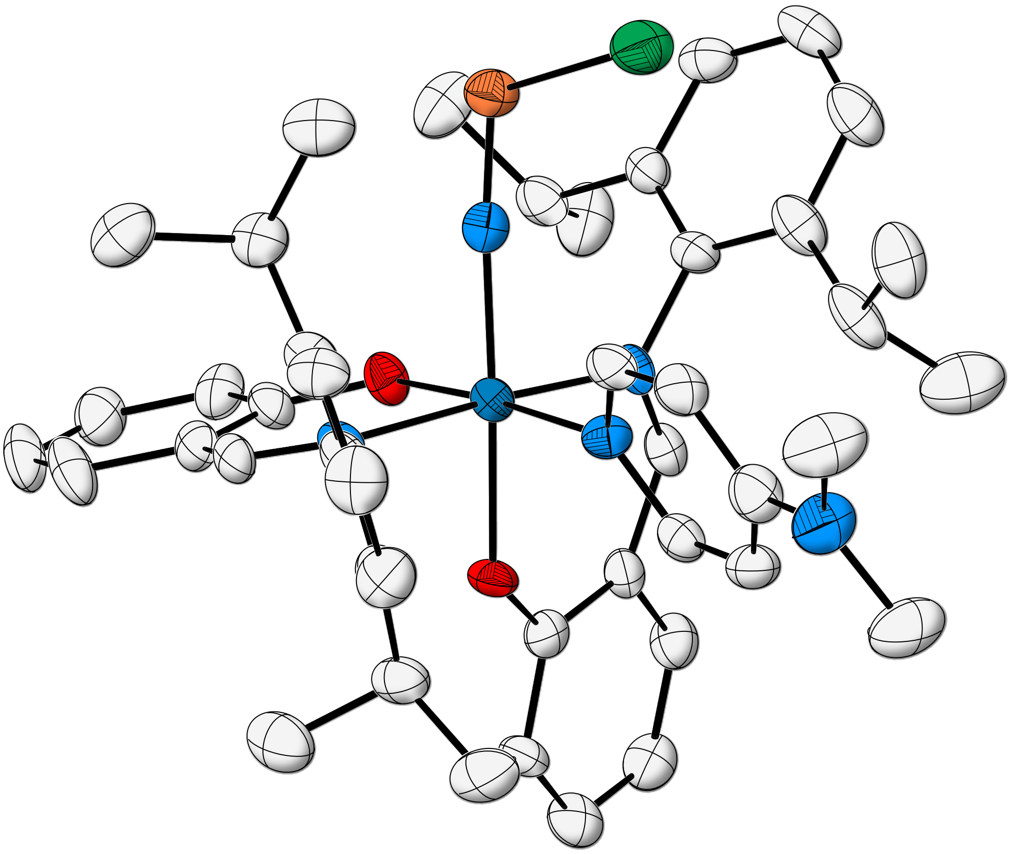

Supplementary Fig. 51. ORTEP plot of [(salNdipp)$_2$(DMAP)Os(NPCl)] (**5**), CCDC entry **2388541**. The diffraction pattern was recorded at 100(2) K, and the thermal ellipsoids are set to 50% probability. H-atoms are omitted. Color code: Light gray (C), light blue (N), red (O), orange (P), green (Cl), turquoise (Os).

8.7 Thermal Ellipsoid Plot of [(salNdipp)$_2$(DMAP)Os(η^1-N_4P)] (6)

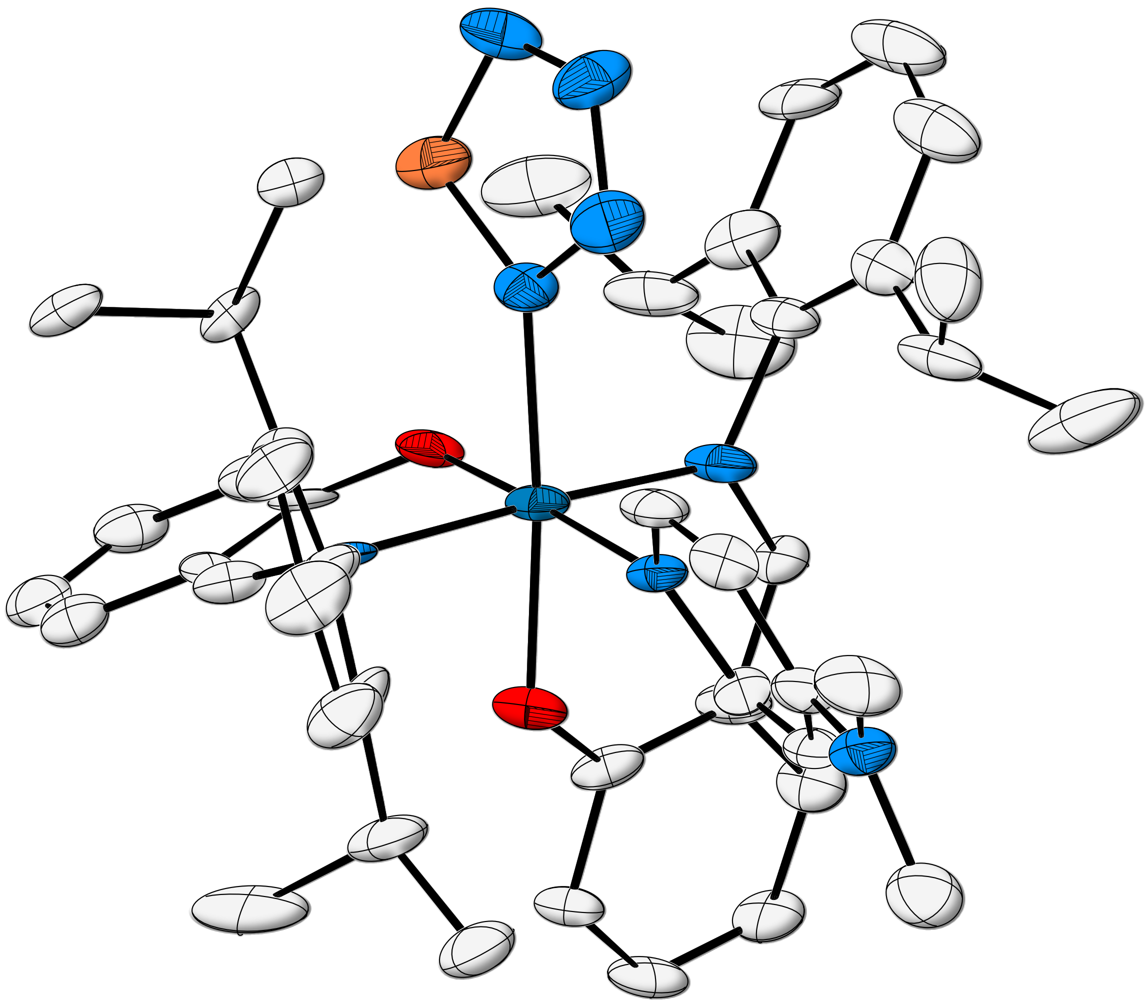

Supplementary Fig. 52. ORTEP plot of [(salNdipp)$_2$(DMAP)Os(η^1-N_4P)] (**6**), CCDC entry **2388540**. The diffraction pattern was recorded at 100(2) K, and the thermal ellipsoids are set to 50% probability. H-atoms are omitted. Color code: Light gray (C), light blue (N), red (O), orange (P), turquoise (Os).

8.8 Thermal Ellipsoid Plot of [(salNdipp)$_2$(DMAP)Os(N_2)] (7)

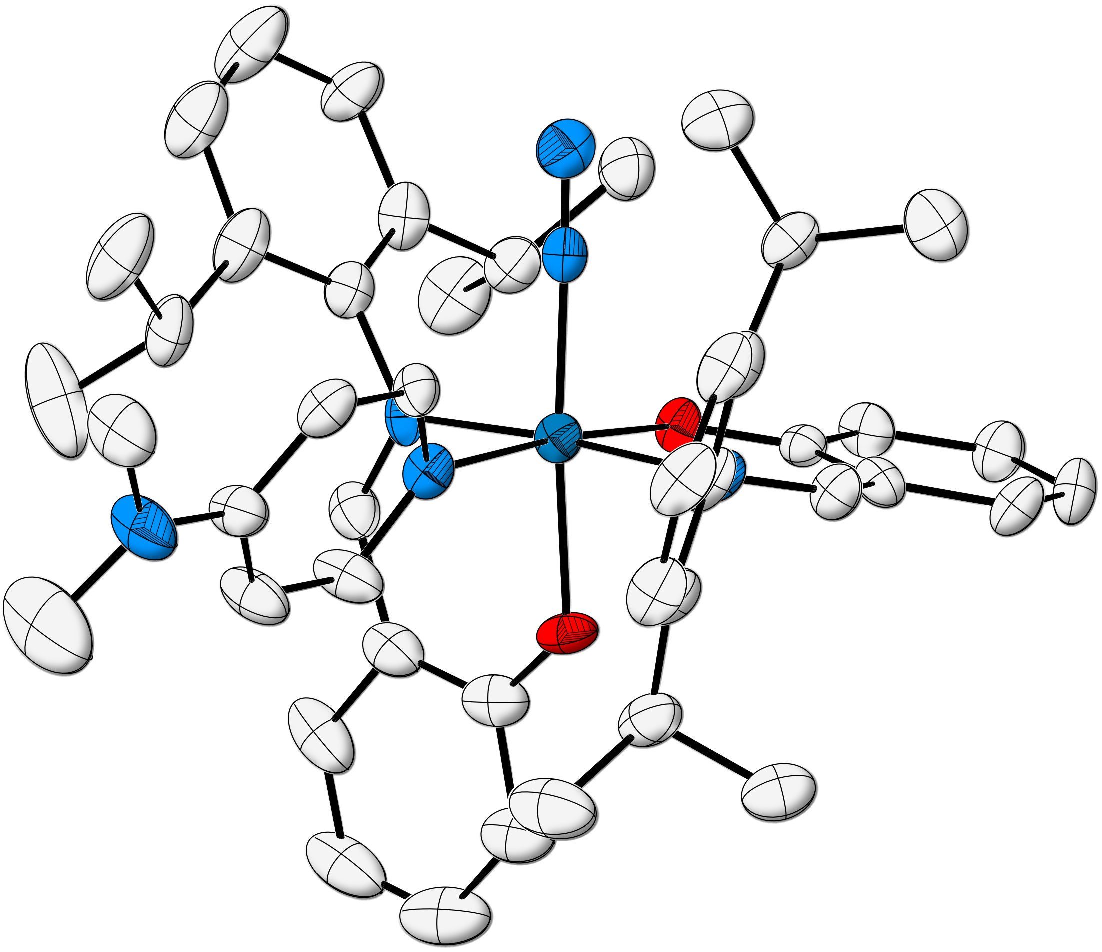

Supplementary Fig. 53. ORTEP plot of [(salNdipp)$_2$(DMAP)Os(N_2)] (**7**), CCDC entry **2388538**. The diffraction pattern was recorded at 100(2) K, and the thermal ellipsoids are set to 50% probability. H-atoms are omitted. Color code: Light gray (C), light blue (N), red (O), turquoise (Os).

8.9 Notes on A- and B-Level Alerts in CheckCif Reports

[(salNdipp)$_2$(Cl)Os≡N] (1)

Alert level B

```
PLAT910_ALERT_3_B Missing # of FCF Reflection(s) Below Theta(Min).         18 Note
                 1  0  0,   1  1  0,   0  2  0,   1  2  0,  -1  1  1,   0  1  1,
                 1  1  1,  -1  2  1,   0  2  1,   1  2  1,   0  3  1,  -1  0  2,
                 0  0  2,   1  0  2,  -1  1  2,   0  1  2,  -1  2  2,   0  2  2,
```

Author Response: These strong reflections were occluded by the beamstop; including them in the data refinement would introduce unphysical observations into the dataset.

[(salNdipp)$_2$(OTf)Os≡N] (2)

Alert level A

```
PLAT971_ALERT_2_A Check Calcd Resid. Dens.  1.00Ang From Os2            4.54 eA-3
```

Author Response: This alert is due to high values for residual electron density. A maximum is located close to an Os atom in a position, which is not realistic for any atom nor a disordered fragment of the molecule. The feature arises from series termination errors, which are common for heavy atoms.

Alert level B

```
PLAT230_ALERT_2_B Hirshfeld Test Diff for    S2       --O8       .         9.7 s.u.
```

Author Response: This alert reveals directional differences in thermal motion between an S and an O atom of a coordinated triflate ligand, which arises due to the relatively free rotation about bonds involving (another) O atom of the triflate which is coordinated to osmium.

```
PLAT910_ALERT_3_B Missing # of FCF Reflection(s) Below Theta(Min).         30 Note
                 1  0  0,  -1  1  0,   0  1  0,   1  1  0,  -1  2  0,   0  2  0,
                 0 -2  1,   1 -2  1,  -1 -1  1,   0 -1  1,   1 -1  1,  -1  0  1,
                 0  0  1,   1  0  1,  -1  1  1,   0  1  1,   1  1  1,  -1  2  1,
                 0  2  1,   0 -2  2,  -1 -1  2,   0 -1  2,   1 -1  2,  -1  0  2,
```

Author Response: These strong reflections were occluded by the beamstop; including them in the data refinement would introduce unphysical observations into the dataset.

```
PLAT971_ALERT_2_B Check Calcd Resid. Dens.  0.99Ang From Os1            2.66 eA-3
```

Author Response: This alert is due to high values for residual electron density. A maximum is located close to an Os atom in a position, which is not realistic for any atom nor a disordered fragment of the molecule. The feature arises from series termination errors, which are common for heavy atoms.

```
PLAT973_ALERT_2_B Check Calcd Positive Resid. Density on       Os2       1.90 eA-3
```

Author Response: This alert is due to high values for residual electron density. A maximum is located close to an Os atom in a position, which is not realistic for any atom nor a disordered fragment of the molecule. The feature arises from series termination errors, which are common for heavy atoms.

[(salNdipp)$_2$(DMAP)Os(NP)] (3)

Alert level B

```
PLAT910_ALERT_3_B Missing # of FCF Reflection(s) Below Theta(Min).         23 Note
                1  0  0,  -1  1  0,   0  1  0,   1  1  0,  -1 -1  1,   0 -1  1,
                1 -1  1,  -1  0  1,   0  0  1,   1  0  1,  -1  1  1,   0  1  1,
                1  1  1,   1  2  1,  -1 -1  2,   0 -1  2,  -1  0  2,   0  0  2,
                1  0  2,   0  1  2,   1  1  2,   0  0  3,   0  1  3,
```

Author Response: These strong reflections were occluded by the beamstop; including them in the data refinement would introduce unphysical observations into the dataset.

```
PLAT971_ALERT_2_B Check Calcd Resid. Dens.  1.05Ang From N3             2.75 eA-3
```

Author Response: This alert is due to high values for residual electron density. A maximum is located between the N atom and an Os atom in a position, which is not realistic for any atom nor a disordered fragment of the molecule. The feature arises from series termination errors, which are common for heavy atoms.

[(salNdipp)$_2$(DMAP)Os(NPS$_2$)] (4)

Alert level B

```
PLAT910_ALERT_3_B Missing # of FCF Reflection(s) Below Theta(Min).         21 Note
                1  0  0,   1  1  0,   2  0  0,   2  1  0,   3  0  0,  -2  1  1,
               -1  1  1,   0  1  1,   1  1  1,   2  1  1,  -3  0  2,  -2  0  2,
               -2  1  2,  -1  0  2,  -1  1  2,   0  0  2,   0  1  2,   1  0  2,
                1  1  2,   2  0  2,  -1  1  3,
```

Author Response: These strong reflections were occluded by the beamstop; including them in the data refinement would introduce unphysical observations into the dataset.

[(salNdipp)$_2$(DMAP)Os(NPCl)] (5)

Alert level B

```
PLAT910_ALERT_3_B Missing # of FCF Reflection(s) Below Theta(Min).         21 Note
                2  0  0,   1  1  0,   2  1  0,   0  2  0,   1  2  0,   1  3  0,
               -1  0  1,   1  0  1,  -2  1  1,  -1  1  1,   0  1  1,   1  1  1,
               -1  2  1,   0  2  1,   1  2  1,  -1  3  1,   0  3  1,   0  0  2,
               -1  1  2,   0  1  2,  -1  2  2,
```

Author Response: These strong reflections were occluded by the beamstop; including them in the data refinement would introduce unphysical observations into the dataset.

[(salNdipp)$_2$(DMAP)Os(η^1-N_4P)] (6)

Alert level B

```
RINTA01_ALERT_3_B  The value of Rint is greater than 0.18
            Rint given    0.249
```

Author Response: This heterocyclic compound is highly sensitive to thermal decomposition. We have studied several other crystals of the same compound, and this was by far the best dataset (lowest Rint) we have collected.

```
PLAT910_ALERT_3_B Missing # of FCF Reflection(s) Below Theta(Min).         18 Note
                 2  0  0,   1  1  0,   0  2  0,   1  2  0,  -1  0  1,   1  0  1,
                -1  1  1,   0  1  1,   1  1  1,  -1  2  1,   0  2  1,   0  0  2,
                -1  1  2,   0  1  2,   1  1  2,  -1  0  3,   1  0  3,   0  1  3,
```

Author Response: These strong reflections were occluded by the beamstop; including them in the data refinement would introduce unphysical observations into the dataset.

[(salNdipp)$_2$(DMAP)Os(N_2)] (7)

Alert level B

```
PLAT910_ALERT_3_B Missing # of FCF Reflection(s) Below Theta(Min).         22 Note
                 1  0  0,  -1  1  0,   0  1  0,   1  1  0,  -1 -1  1,   0 -1  1,
                -1  0  1,   0  0  1,   1  0  1,  -1  1  1,   0  1  1,   1  1  1,
                 1  2  1,  -1 -1  2,   0 -1  2,  -1  0  2,   0  0  2,   1  0  2,
                 0  1  2,   1  1  2,   0  0  3,   0  1  3,
```

Author Response: These strong reflections were occluded by the beamstop; including them in the data refinement would introduce unphysical observations into the dataset.

```
PLAT971_ALERT_2_B Check Calcd Resid. Dens.   0.94Ang From Os1             3.08 eA-3
```

Author Response: This alert is due to high values for residual electron density. A maximum is located close to an Os atom in a position, which is not realistic for any atom nor a disordered fragment of the molecule. The feature arises from series termination errors, which are common for heavy atoms.

9 Computational Studies

9.1. Computational methodology

Orca software v.5.0.3 was employed to perform density functional theory (DFT) calculations.[12, 13] Gas-phase geometry optimizations on non-truncated models were carried out using the PBE0 functional[14] combined with the def2-TZVP(-f) basis set[15, 16] and the auxiliary basis set def2-J.[15] The resolution of the identity approximation for Coulomb and chain of spheres approximation for exchange interactions (RIJCOSX) was employed to accelerate geometry optimizations.[17] Grimme's D3 method and the Becke-Johnson (D3BJ) damping scheme were used to consider dispersion effects in all calculations.[18] A tight convergence of the wavefunction was requested on grid quality defgrid2.

Geometry optimizations were followed by analytical frequency calculations at the same level of theory (PBE0-D3/ def2-TZVP(-f)/defgrid2) to confirm that the resulting equilibrium structures were in the minima of the potential energy surface. Subsequent single-point calculations were performed to re-evaluate the electronic energy and wavefunction of the molecules. The meta GGA hybrid TPSSh functional in combination with the def2-TZVP basis set was employed on defgrid2.[19-21] The electronic structure of species was scrutinized, including MOs and Mayer bond orders, Wiberg bond orders and Natural Bond Orbital (NBO) analyses (using NBO3[22]) as implemented in Gaussian16[23]) at the TPSSh-D3/def2-TZVP/defgrid2 level of theory. Besides, quantum theory of atoms in molecules (QTAIM) and effective oxidation state (EOS) analyses were performed using Multiwfn v. 3.8[24] and APOST-3D[25], respectively.

9.2 Computational data

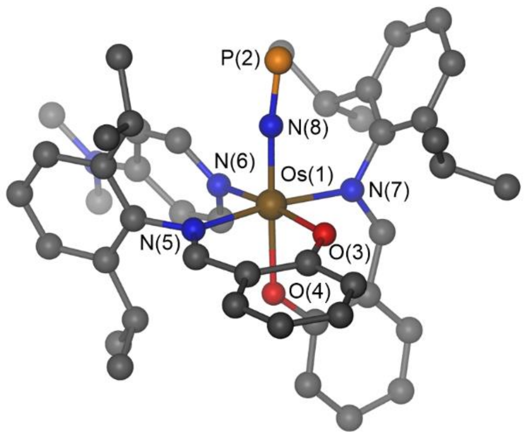

Supplementary Table 5. Calculated distances (Å) and angles (◦) of [(salNdipp)$_2$(DMAP)Os(NP)] (**3**). Hydrogen atoms are omitted for clarity. (PBE0-D3/def2-TZVP(-f))

**Metric**	**XRD**	**Calc.**	**Δ**
D(1-3)	2.042	2.034	0.008
D(1-4)	2.053	2.054	0.001
D(1-5)	2.084	2.100	0.016
D(1-6)	2.106	2.088	0.018
D(1-7)	2.097	2.090	0.007
D(2-8)	1.531	1.519	0.012
D(1-8)	1.851	1.843	0.008
A(3-1-4)	84	83	1
A(3-1-5)	91	91	0
A(3-1-6)	173	172	1
A(3-1-7)	86	85	1
A(3-1-8)	93	94	1
A(4-1-5)	84	85	1
A(4-1-6)	90	89	1
A(4-1-7)	91	89	2
A(4-1-8)	177	177	0
A(5-1-6)	91	90	1
A(5-1-7)	174	173	1
A(5-1-8)	95	95	0
A(6-1-7)	92	93	1
A(6-1-8)	93	93	0
A(7-1-8)	90	91	1
A(2-8-1)	172	173	1

Supplementary Table 6. Calculated Mayer bond orders (MBO) and Wiberg bond orders (WBO) of [(salNdipp)$_2$(DMAP)Os(NP)] (**3**) at the TPSSh-D3/def2-TZVP level of theory

**Metric**	**MBO**	**WBO**
Os(1)-O(3)	0.5427	0.7209
Os(1)-O(4)	0.4500	0.6563
Os(1)-N(5)	0.5732	0.6782
Os(1)-N(6)	0.5811	0.6886
Os(1)-N(7)	0.5874	0.6990
P(2)-N(8)	2.2159	2.7263
Os(1)-N(8)	1.2077	1.5386

Supplementary Table 7. Natural Bond Orbital (NBO) analysis of Alpha Spin Orbitals for [(salNdipp)$_2$(DMAP)Os(NP)] (**3**) at the TPSSh-D3/def2-TZVP level of theory. Occupancy, orbital characterization, atomic contributions and weighting factors that characterize the Os(NP) bonding are provided (Values for Other Atoms Omitted for Clarity).

NBO index	Occupancy	NBO Type	Atoms	Contribution (%) / Weighting factor
1	0.95856	BD* (1)	Os(1)-P(2)	Os(1): (71.87%) / 0.8478<br>P(2): (28.13%) / -0.5303
2	0.92429	BD* (2)	Os(1)-P(2)	Os(1): (75.35%) / 0.8681<br>P(2): (24.65%) / -0.4965
4	0.99072	BD (1)	Os(1)-N(8)	Os(1): (26.07%) / 0.5106<br>N(8): (73.93%) / 0.8598
5	0.98590	BD (1)	P(2)-N(8)	P(2): (25.21%) / 0.5021<br>N(8): (74.79%) / 0.8648
188	0.90926	LP (1)	Os(1)	
189	0.22605	LP* (2)	Os(1)	
190	0.08055	LP* (3)	Os(1)	
191	0.07501	LP* (4)	Os(1)	
192	0.06007	LP* (5)	Os(1)	
193	0.97701	LP (1)	P(2)	
201	0.68714	LP (1)	N(8)	
202	0.68163	LP (2)	N(8)	
1888	0.33382	BD (1)	Os(1)-P(2)	Os(1): (28.13%) / 0.5303<br>P(2): (71.87%) / 0.8478
1889	0.32708	BD (2)	Os(1)-P(2)	Os(1): (24.65%) / 0.4965<br>P(2): (75.35%) / 0.8681
1891	0.16173	BD* (1)	Os(1)-N(8)	Os(1): (73.93%) / 0.8598<br>N(8): (26.07%) / -0.5106
1892	0.00895	BD* (1)	P(2)-N(8)	P(2): (74.79%) / 0.8648<br>N(8): (25.21%) / -0.5021

Supplementary Table 8. Natural Bond Orbital (NBO) analysis of Beta Spin Orbitals for [(salNdipp)$_2$(DMAP)Os(NP)] (**3**) at the TPSSh-D3/def2-TZVP level of theory. Occupancy, orbital characterization, atomic contributions and weighting factors that characterize the Os(NP) bonding are provided (Values for Other Atoms Omitted for Clarity).

NBO index	Occupancy	NBO Type	Atoms	Contribution (%) / Weighting factor
1	0.95856	BD* (1)	Os(1)−P(2)	Os(1): 71.87% / 0.8478<br>P(2): 28.13% / −0.5303
2	0.92429	BD* (2)	Os(1)−P(2)	Os(1): 75.35% / 0.8681<br>P(2): 24.65% / −0.4965
4	0.99072	BD (1)	Os(1)−N(8)	Os(1): 26.07% / 0.5106<br>N(8): 73.93% / 0.8598
5	0.98590	BD (1)	P(2)−N(8)	P(2): 25.21% / 0.5021<br>N(8): 74.79% / 0.8648
188	0.90926	LP (1)	Os(1)	
189	0.22605	LP* (2)	Os(1)	
190	0.08055	LP* (3)	Os(1)	
191	0.07501	LP* (4)	Os(1)	
192	0.06007	LP* (5)	Os(1)	
193	0.97701	LP (1)	P(2)	
201	0.68714	LP (1)	N(8)	
202	0.68163	LP (2)	N(8)	
1888	0.33382	BD (1)	Os(1)−P(2)	Os(1): 28.13% / 0.5303<br>P(2): 71.87% / 0.8478
1889	0.32708	BD (2)	Os(1)−P(2)	Os(1): 24.65% / 0.4965<br>P(2): 75.35% / 0.8681
1891	0.16173	BD* (1)	Os(1)−N(8)	Os(1): 73.93% / 0.8598<br>N(8): 26.07% / −0.5106
1892	0.00895	BD* (1)	P(2)−N(8)	P(2): 74.79% / 0.8648<br>N(8): 25.21% / −0.5021

This NBO analysis supports the cumulenic bonding structure established for the [Os^{IV}=N=P] functionality: It shows two low occupancy lone pairs localized on the nitrogen, two Os–P 'antibonding' interactions that are significantly Os-based (70–75%), the corresponding bonding combinations are also semi filled (0.32–0.33) and P-centered, a P-based lone-pair, a P–N σ bond and an Os–N σ bond – together these local NBOs characterize an [Os=N=P] fragment.

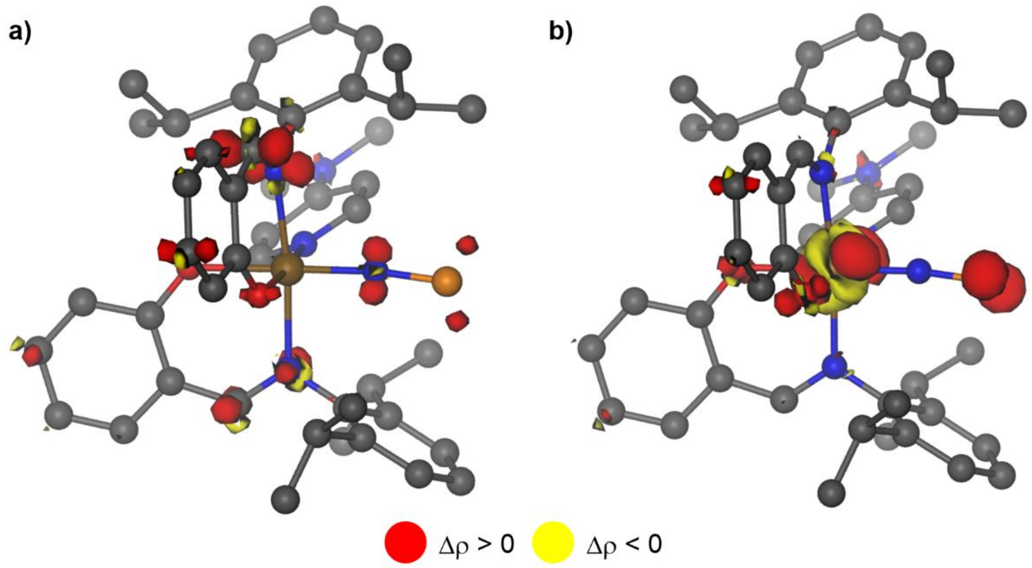

Supplementary Fig. 54. Calculated Fukui functions for a) nucleophilic attack ($f^+(r)$) and b) Electrophilic attack ($f^-(r)$) of [(salNdipp)$_2$(DMAP)Os(NP)] (**3**) generated at the TPSSh-D3/def2-TZVP level of theory. The isosurface is set to 0.004 a.u.

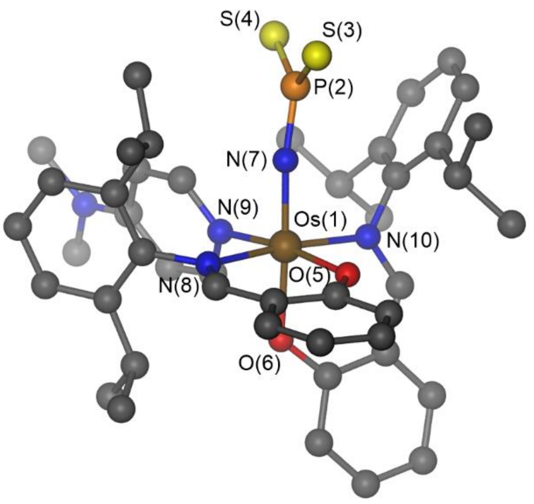

Supplementary Table 9. Calculated distances (Å) and angles (◦) of [(salNdipp)$_2$(DMAP)Os(NPS$_2$)] (**4**). Hydrogen atoms are omitted for clarity. (PBE0-D3/def2-TZVP(-f))

**Metric**	**XRD**	**Calc.**	**Δ**
D(1-5)	2.002	1.977	0.025
D(1-6)	1.992	2.015	0.023
D(1-7)	1.880	1.832	0.048
D(1-8)	2.101	2.075	0.026
D(1-9)	2.124	2.123	0.001
D(1-10)	2.102	2.104	0.002
D(2-7)	1.551	1.566	0.015
D(2-4)	1.935	1.931	0.004
D(2-3)	1.937	1.929	0.008
A(5-1-6)	86	85	1
A(5-1-7)	98	99	1
A(5-1-8)	92	92	0
A(5-1-9)	171	170	1
A(5-1-10)	82	83	1
A(6-1-7)	176	176	0
A(6-1-8)	82	87	5
A(6-1-9)	86	85	0
A(6-1-10)	90	89	0
A(7-1-8)	96	94	3
A(7-1-9)	90	90	0
A(7-1-10)	93	91	2
A(8-1-9)	90	89	1
A(8-1-10)	170	174	4
A(9-1-10)	95	95	0
A(1-7-2)	173	170	3
A(4-2-3)	124	128	4

Supplementary Table 10. Calculated Mayer bond orders (MBO) and Wiberg bond orders (WBO) of [(salNdipp)$_2$(DMAP)Os(NPS$_2$)] (**4**) at the TPSSh-D3/def2-TZVP level of theory

Metric	MBO	WBO
Os(1)−O(5)	0.6629	0.8323
Os(1)−O(6)	0.4855	0.7421
Os(1)−N(7)	1.3038	1.6349
Os(1)−N(8)	0.6328	0.7283
Os(1)−N(9)	0.5560	0.6341
Os(1)−N(10)	0.6071	0.6714
P(2)−N(7)	1.4330	1.8886
P(2)−S(4)	1.6587	2.2126
P(2)−S(3)	1.6688	2.2284

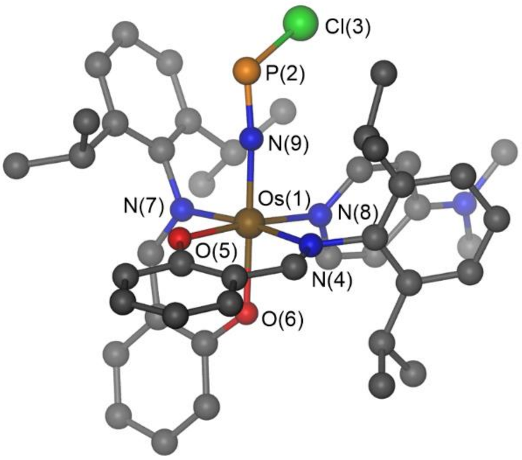

Supplementary Table 11. Calculated distances (Å) and angles (◦) of [(salNdipp)$_2$(DMAP)Os(NPCl)] (**5**). Hydrogen atoms are omitted for clarity. (PBE0-D3/def2-TZVP(-f))

**Metric**	**XRD**	**Calc.**	**Δ**
D(1-4)	2.096	2.085	0.011
D(1-5)	2.020	2.005	0.015
D(1-6)	2.018	2.028	0.010
D(1-7)	2.104	2.091	0.013
D(1-8)	2.106	2.098	0.008
D(1-9)	1.942	1.907	0.035
D(2-9)	1.494	1.519	0.025
D(2-3)	2.299	2.224	0.075
A(4-1-5)	91	92	0
A(4-1-6)	85	85	0
A(4-1-7)	173	173	0
A(4-1-8)	90	90	0
A(4-1-9)	94	94	0
A(5-1-6)	86	85	1
A(5-1-7)	84	84	0
A(5-1-8)	175	174	1
A(5-1-9)	92	94	2
A(6-1-7)	90	90	0
A(6-1-8)	89	89	0
A(6-1-9)	178	179	1
A(7-1-8)	95	94	1
A(7-1-9)	91	91	0
A(8-1-9)	93	92	1
A(1-9-2)	174	174	0
A(3-2-9)	110	110	0

Supplementary Table 12. Calculated Mayer bond orders (MBO) and Wiberg bond orders (WBO) of [(salNdipp)$_2$(DMAP)Os(NPCl)] (**5**) at the TPSSh-D3/def2-TZVP level of theory

Metric	MBO	WBO
Os(1)−N(4)	0.6113	0.7065
Os(1)−O(5)	0.6149	0.7904
Os(1)−O(6)	0.5145	0.7322
Os(1)−N(7)	0.6003	0.7013
Os(1)−N(8)	0.5668	0.6705
Os(1)−N(9)	0.9800	1.3217
P(2)−N(9)	0.8242	2.5748
P(2)−Cl(3)	0.8242	1.3510

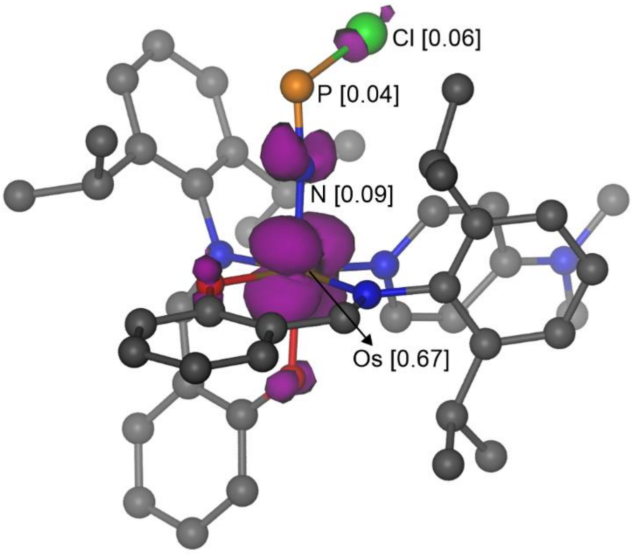

Supplementary Fig. 55. Spin density plot and Löwdin atomic spin densities of doublet species [(salNdipp)$_2$(DMAP)Os(NPCl)] (**5**) at the TPSSh-D3/def2-TZVP level of theory. The isosurface is set to ± 0.006.

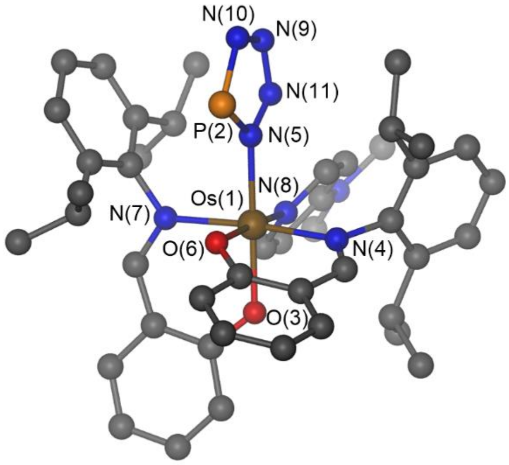

Supplementary Table 13. Calculated distances (Å) and angles (◦) of [(salNdipp)$_2$(DMAP)Os(η^1-N$_4$P)] (**6**). Hydrogen atoms are omitted for clarity. (PBE0-D3/def2-TZVP(-f))

**Metric**	**XRD**	**Calc.**	**Δ**
D(1-3)	2.014	2.011	0.003
D(1-4)	2.079	2.076	0.003
D(1-5)	2.069	2.060	0.009
D(1-6)	2.013	1.990	0.023
D(1-7)	2.065	2.084	0.019
D(1-8)	2.103	2.113	0.010
D(2-5)	1.700	1.681	0.019
D(2-10)	1.614	1.641	0.027
D(9-10)	1.313	1.320	0.007
D(9-11)	1.336	1.299	0.037
D(5-11)	1.326	1.326	0.000
A(3-1-4)	83	84	0
A(3-1-5)	173	177	3
A(3-1-6)	88	88	0
A(3-1-7)	92	92	0
A(3-1-8)	88	87	1
A(4-1-5)	95	95	0
A(4-1-6)	90	91	0
A(4-1-7)	174	174	0
A(4-1-8)	92	93	1
A(5-1-6)	85	89	3
A(5-1-7)	89	89	0
A(5-1-8)	99	96	3
A(6-1-7)	86	85	1
A(6-1-8)	175	174	1
A(7-1-8)	92	92	0

Supplementary Table 14. Calculated Mayer bond orders (MBO) and Wiberg bond orders (WBO) of [(salNdipp)$_2$(DMAP)Os(η^1-N$_4$P)] (**6**) at the TPSSh-D3/def2-TZVP level of theory

Metric	MBO	WBO
Os(1)–O(3)	0.6123	0.7874
Os(1)–N(4)	0.6512	0.7379
Os(1)–N(5)	0.6330	0.8208
Os(1)–O(6)	0.6622	0.8598
Os(1)–N(7)	0.6197	0.7276
Os(1)–N(8)	0.5514	0.6582
P(2)–N(5)	1.2782	1.5574
P(2)–N(10)	1.4923	1.8532
N(9)–N(10)	1.2637	1.8535
N(9)–N(11)	1.3871	1.9472
N(5)–N(11)	1.1186	1.7133

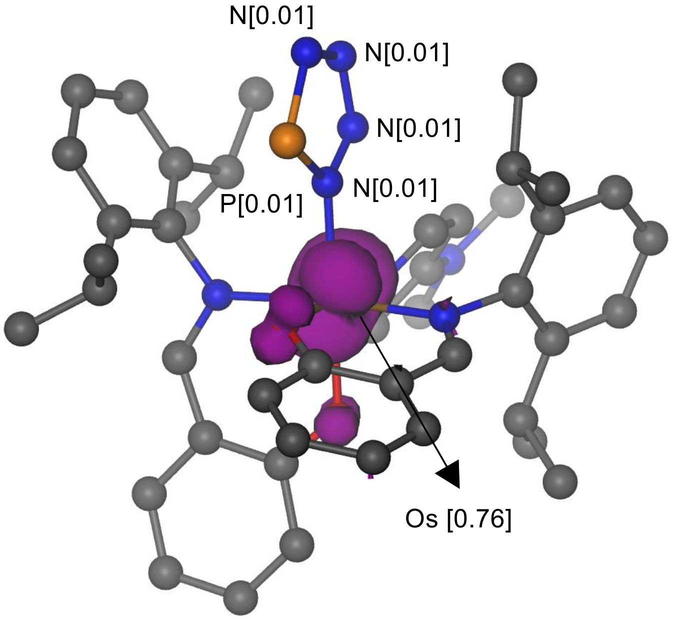

Supplementary Fig. 56. Spin density plot and Löwdin atomic spin densities of doublet species [(salNdipp)$_2$(DMAP)Os(η^1-N$_4$P)] (**6**) at the TPSSh-D3/def2-TZVP level of theory. The isosurface is set to ± 0.006.

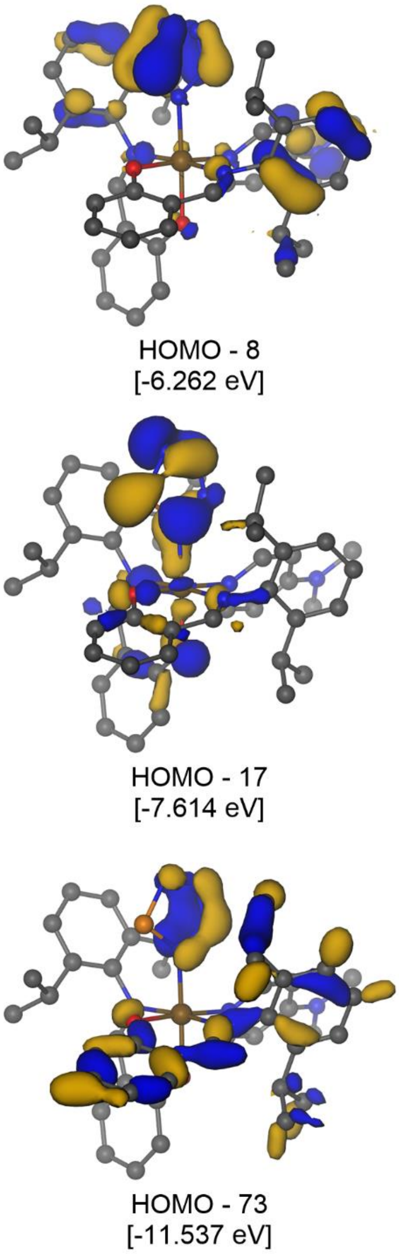

Supplementary Fig. 57. Quasi-restricted orbitals (QROs) showing the Os–N σ interaction (HOMO -17) and the aromatic heterocycle character (HOMO -8, HOMO -17 and HOMO -73) on the $[PN_4]^-$ ligand of doublet species $[(salNdipp)_2(DMAP)Os(\eta^1\text{-}N_4P)]$ (**6**) at the TPSSh-D3/def2-TZVP level of theory. The isosurface is set to ± 0.04.

Supplementary Table 15. Natural Bond Orbital (NBO) analysis of Alpha Spin Orbitals for [(salNdipp)$_2$(DMAP)Os(η^1-N$_4$P)] (**6**) at the TPSSh-D3/def2-TZVP level of theory. Occupancy, orbital characterization, atomic contributions and weighting factors that characterize the Os(η^1-N$_4$P) bonding are provided (Values for Other Atoms Omitted for Clarity).

NBO index	Occupancy	NBO Type	Atoms	Contribution (%) / Weighting factor
1	0.98593	BD (1)	Os(1)-N(5)	Os(1): 23.39% / 0.4836<br>N(5): 76.61% / 0.8753
2	0.98248	BD (1)	P(2)-N(5)	P(2): 25.62% / 0.5062<br>N(5): 74.38% / 0.8624
3	0.91535	BD (2)	P(2)-N(5)	P(2): 31.38% / 0.5602<br>N(5): 68.62% / 0.8284
4	0.98631	BD (1)	P(2)-N(10)	P(2): 29.69% / 0.5449<br>N(19): 70.31% / 0.8385
9	0.99263	BD (1)	N(5)-N(11)	N(5): 52.39% / 0.7238<br>N(35): 47.61% / 0.6900
16	0.99345	BD (1)	N(9)-N(10)	N(9): 50.15% / 0.7082<br>N(19): 49.85% / 0.7060
17	0.91979	BD (2)	N(9)-N(10)	N(9): 44.91% / 0.6701<br>N(19): 55.09% / 0.7423
18	0.99271	BD (1)	N(9)-N(11)	N(9): 49.02% / 0.7002<br>N(35): 50.98% / 0.7140
194	0.96696	LP (1)	Os(1)	
195	0.92011	LP (2)	Os(1)	
196	0.89553	LP (3)	Os(1)	
197	0.41654	LP* (4)	Os(1)	
198	0.17295	LP* (5)	Os(1)	
199	0.08388	LP* (6)	Os(1)	
200	0.06879	LP* (7)	Os(1)	
201	0.06399	LP* (8)	Os(1)	
202	0.96808	LP (1)	P(2)	
212	0.95532	LP (1)	N(9)	
216	0.96679	LP (1)	N(10)	
218	0.94403	LP (1)	N(11)	
219	0.59582	LP (2)	N(11)	

Supplementary Table 16. Natural Bond Orbital (NBO) analysis of Alpha Spin Orbitals for [(salNdipp)$_2$(DMAP)Os(η^1-N$_4$P)] (**6**) at the TPSSh-D3/def2-TZVP level of theory. Occupancy, orbital characterization, atomic contributions and weighting factors that characterize the Os(η^1-N$_4$P) bonding are provided (Values for Other Atoms Omitted for Clarity). (cont.)

NBO index	Occupancy	NBO Type	Atoms	Contribution (%) / Weighting factor
1978	0.18869	BD* (1)	Os(1)-N(5)	Os(1): 76.61% / 0.8753<br>N(5): 23.39% / -0.4836
1979	0.04189	BD* (1)	P(2)-N(5)	P(2): 74.38% / 0.8624<br>N(5): 25.62% / -0.5062
1980	0.28134	BD* (2)	P(2)-N(5)	P(2): 68.62% / 0.8284<br>N(5): 31.38% / -0.5602
1981	0.02630	BD* (1)	P(2)-N(10)	P(2): 70.31% / 0.8385<br>N(19): 29.69% / -0.5449
1986	0.03306	BD* (1)	N(5)-N(11)	N(5): 47.61% / 0.6900<br>N(35): 52.39% / -0.7238
1993	0.02312	BD* (1)	N(9)-N(10)	N(9): 49.85% / 0.7060<br>N(19): 50.15% / -0.7082
1994	0.29278	BD* (2)	N(9)-N(10)	N(9): 55.09% / 0.7423<br>N(19): 44.91% / -0.6701
1995	0.02499	BD* (1)	N(9)-N(11)	N(9): 50.98% / 0.7140<br>N(35): 49.02% / -0.7002

Supplementary Table 17. Natural Bond Orbital (NBO) analysis of Beta Spin Orbitals for [(salNdipp)$_2$(DMAP)Os(η^1-N_4P)] (**6**) at the TPSSh-D3/def2-TZVP level of theory. Occupancy, orbital characterization, atomic contributions and weighting factors that characterize the Os(η^1-N_4P) bonding are provided (Values for Other Atoms Omitted for Clarity).

NBO index	Occupancy	NBO Type	Atoms	Contribution (%) / Weighting factor
2	0.97751	BD (1)	P(2)-N(5)	P(2): 25.29% / 0.5029<br>N(5): 74.71% / 0.8643
3	0.98413	BD (1)	P(2)- N(10)	P(2): 29.60% / 0.5441<br>N(19): 70.40% / 0.8390
4	0.90059	BD (2)	P(2)-N(10)	P(2): 38.05% / 0.6169<br>N(19): 61.95% / 0.7871
9	0.99040	BD (1)	N(5)-N(11)	N(5): 52.58% / 0.7251<br>N(35): 47.42% / 0.6886
10	0.93930	BD (2)	N(5)-N(11)	N(5): 60.31% / 0.7766<br>N(35): 39.69% / 0.6300
17	0.99347	BD (1)	N(9)-N(10)	N(9): 50.14% / 0.7081<br>N(19): 49.86% / 0.7061
18	0.99208	BD (1)	N(9)-N(11)	N(9): 49.04% / 0.7003<br>N(35): 50.96% / 0.7139
194	0.90314	LP (1)	Os(1)	
195	0.86889	LP (2)	Os(1)	
196	0.40741	LP* (3)	Os(1)	
197	0.38892	LP* (4)	Os(1)	
198	0.14037	LP* (5)	Os(1)	
199	0.07696	LP* (6)	Os(1)	
200	0.06588	LP* (7)	Os(1)	
201	0.06299	LP* (8)	Os(1)	
202	0.96804	LP (1)	P(2)	
207	0.80686	LP (1)	N(5)	
212	0.95228	LP (1)	N(9)	
213	0.57040	LP (2)	N(9)	
217	0.96111	LP (1)	N(10)	
220	0.94387	LP (1)	N(11)	

Supplementary Table 18. Natural Bond Orbital (NBO) analysis of Beta Spin Orbitals for [(salNdipp)$_2$(DMAP)Os(η^1-N$_4$P)] (**6**) at the TPSSh-D3/def2-TZVP level of theory. Occupancy, orbital characterization, atomic contributions and weighting factors that characterize the Os(η^1-N$_4$P) bonding are provided (Values for Other Atoms Omitted for Clarity). (cont.)

NBO index	Occupancy	NBO Type	Atoms	Contribution (%) / Weighting factor
1979	0.04036	BD* (1)	P(2)-N(5)	P(2): 74.71% / 0.8643<br>N(5): 25.29% / -0.5029
1980	0.02667	BD* (1)	P(2)-N(10)	P(2): 70.40% / 0.8390<br>N(19): 29.60% / -0.5441
1981	0.21386	BD* (2)	P(2)-N(10)	P(2): 61.95% / 0.7871<br>N(19): 38.05% / -0.6169
1986	0.03370	BD* (1)	N(5)-N(11)	P(5): 47.42% / 0.6886<br>N(19): 52.58% / -0.7251
1987	0.37090	BD* (2)	N(5)-N(11)	N(5): 39.69% / 0.6300<br>N(35): 60.31% / -0.7766
1994	0.02294	BD* (1)	N(9)-N(10)	N(9): 49.86% / 0.7061<br>N(19): 50.14% / -0.7081
1995	0.02541	BD* (1)	N(9)-N(11)	N(9): 50.96% / 0.7139<br>N(35): 49.04% / -0.7003

Supplementary Table 19. Solution state reaction Gibbs free energies for the possible decomposition pathways of [(salNdipp)$_2$(DMAP)Os(η^1-N$_4$P)] (**6**). Calculations are carried out at the TPSSh-D3/def2-TZVP level of theory in benzene. The SMD solvation model was employed to account for solvent effects.

Pathway	ΔG^0(sol) -TPSSh
[(salNdipp)$_2$(DMAP)Os(η^1-N$_4$P)] → [(salNdipp)$_2$(DMAP)Os(NP)] + N$_3^\bullet$	35.87
[(salNdipp)$_2$(DMAP)Os(η^1-N$_4$P)] → [(salNdipp)$_2$(DMAP)Os(N$_2$)] + [PNN]$^\bullet$	-2.34
[(salNdipp)$_2$(DMAP)Os(η^1-N$_4$P)] → [(salNdipp)$_2$(DMAP)Os(N$_2$)] + [NPN]$^\bullet$	57.37

10 References

(1) Cowman, C. D., Trogler, W. C., Mann, K. R., Poon, C. K. & Gray, H. B. Electronic-Structures and Spectra of Nitrido Complexes of Osmium(VI). *Inorg. Chem.* **15**, 1747-1751 (1976).

(2) Chang, S., Jones, L., Wang, C., Henling, L. M. & Grubbs, R. H. Synthesis and Characterization of New Ruthenium-Based Olefin Metathesis Catalysts Coordinated with Bidentate Schiff-Base Ligands. *Organometallics* **17**, 3460-3465 (1998).

(3) Heift, D., Benkő, Z. & Grützmacher, H. Coulomb repulsion versus cycloaddition: formation of anionic four-membered rings from sodium phosphaethynolate, Na(OCP). *Dalton Trans.* **43**, 831-840 (2014).

(4) Ottmers, D. M. & Rase, H. F. Potassium graphites prepared by mixed-reaction technique. *Carbon* **4**, 125-127 (1966).

(5) *CrysAlis Pro*; Agilent Technologies Ltd: (2014).

(6) Sheldrick, G. M. SHELXT – Integrated space-group and crystal-structure determination. *Acta Crystallogr., Sect. A* **71**, 3-8 (2015).

(7) Sheldrick, G. M. Crystal structure refinement with SHELXL. *Acta Crystallogr., Sect. C* **71**, 3-8 (2015).

(8) Dolomanov, O. V., Bourhis, L. J., Gildea, R. J., Howard, J. A. K. & Puschmann, H. OLEX2: a complete structure solution, refinement and analysis program. *J. Appl. Crystallogr.* **42**, 339-341 (2009).

(9) van der Sluis, P. & Spek, A. L. BYPASS: an effective method for the refinement of crystal structures containing disordered solvent regions. *Acta Crystallogr., Sect. A* **46**, 194-201 (1990).

(10) Bain, G. A. & Berry, J. F. Diamagnetic Corrections and Pascal's Constants. *J. Chem. Educ.* **85**, 532-536 (2008).

(11) Pedersen, K. S., et al., Iridates from the molecular side. *Nat. Commun.* **7**, 12195 (2016).

(12) Neese, F. The ORCA program system. *WIREs Comput. Mol. Sci.* **2**, 73-78 (2012).

(13) Neese, F. Software update: The ORCA program system—Version 5.0. *WIREs Comput. Mol. Sci.* **12**, e1606 (2022).

(14) Adamo, C. & Barone, V. Toward reliable density functional methods without adjustable parameters: The PBE0 model. *J. Chem. Phys.* **110**, 6158-6170 (1999).

(15) Weigend, F. Accurate Coulomb-fitting basis sets for H to Rn. *Phys. Chem. Chem. Phys.* **8**, 1057-1065 (2006).

(16) Weigend, F. & Ahlrichs, R. Balanced basis sets of split valence, triple zeta valence and quadruple zeta valence quality for H to Rn: Design and assessment of accuracy. *Phys. Chem. Chem. Phys.* **7**, 3297-3305 (2005).

(17) Neese, F., Wennmohs, F., Hansen, A. & Becker, U. Efficient, approximate and parallel Hartree–Fock and hybrid DFT calculations. A ‘chain-of-spheres’ algorithm for the Hartree–Fock exchange. *Chem. Phys.* **356**, 98-109 (2009).

(18) Grimme, S., Antony, J., Ehrlich, S. & Krieg, H. A consistent and accurate ab initio parametrization of density functional dispersion correction (DFT-D) for the 94 elements H-Pu. *J. Chem. Phys.* **132**, 154104 (2010).

(19) Staroverov, V. N., Scuseria, G. E., Tao, J. & Perdew, J. P. Comparative assessment of a new nonempirical density functional: Molecules and hydrogen-bonded complexes. *J. Chem. Phys.* **119**, 12129-12137 (2003).
(20) Staroverov, V. N., Scuseria, G. E., Tao, J. & Perdew, J. P. Erratum: "Comparative assessment of a new nonempirical density functional: Molecules and hydrogen-bonded complexes". *J. Chem. Phys.* **121**, 11507 (2004).
(21) Tao, J., Perdew, J. P., Staroverov, V. N. & Scuseria, G. E. Climbing the Density Functional Ladder: Nonempirical Meta–Generalized Gradient Approximation Designed for Molecules and Solids. *Phys. Rev. Lett.* **91**, 146401 (2003).
(22) Glendening, E. D., Reed, A. E., Carpenter, J. E. & Weinhold, F. NBO Version 3.1. (2003).
(23) Frisch, M. J. T., et al., Gaussian 16, Revision B.01. (2016).
(24) Lu, T. & Chen, F. Multiwfn: A multifunctional wavefunction analyzer. *J. Comput. Chem.* **33**, 580-592 (2012).
(25) Ramos-Cordoba, E., Postils, V. & Salvador, P. Oxidation States from Wave Function Analysis. *J. Chem. Theory Comput.* **11**, 1501-1508 (2015).
