# Supplementary material for: Unleashing phosphorus mononitride
Source: Nat Commun. 2025 Jul 1;16:5596. doi: 10.1038/s41467-025-60669-6 (PMC12215955; doi:10.1038/s41467-025-60669-6)
Supplement: Supplementary file 2 — Description of Additional Supplementary Files [file 41467_2025_60669_MOESM2_ESM.pdf]

Description of Additional Supplementary Files

File name: Supplementary Data 1

Description: NMR CSV file (exported from MestReNova) containing ^{1}H-^{15}N HMBC NMR data for complex **4-^{15}N** (600/61 MHz, THF-d_8). Column A represents the f1 dimension (δ for ^{15}N, 940–969.99 ppm), and Row 1 represents the f2 dimension (δ for ^{1}H, 0.99–8.01095 ppm). The data spanned between cell B2 and cell AHO769 contain the contour values of the 2D NMR plot.
